# Spatial Heterogeneity of Tick‐Borne Pathogens Outpaces Genetic Structuring in Anatolian *Dermacentor reticulatus* Populations

**DOI:** 10.1155/tbed/5552728

**Published:** 2026-07-22

**Authors:** Ömer Orkun, Maide Nur Gündoğdu, Tuğba Özdemir, Mesut Yiğit, Barış Yıldız, Ahmet Deniz, Zati Vatansever

**Affiliations:** ^1^ Department of Parasitology, Faculty of Veterinary Medicine, Ankara University, Ankara, Türkiye, ankara.edu.tr; ^2^ Department of Parasitology, Graduate School of Health Sciences, Ankara University, Ankara, Türkiye, ankara.edu.tr; ^3^ Department of Parasitology, Faculty of Veterinary Medicine, Kafkas University, Kars, Türkiye, kafkas.edu.tr; ^4^ Life Sciences and Technology Implementation and Research Center, Kafkas University, Kars, Türkiye, kafkas.edu.tr; ^5^ Department of Medical Parasitology, Faculty of Medicine, Kafkas University, Kars, Türkiye, kafkas.edu.tr

**Keywords:** Anatolia, *cox1*, *Dermacentor reticulatus*, ITS2, phylogeography, population genetics, sympatric *Dermacentor* species, tick-borne pathogens

## Abstract

Understanding how vector population structure influences pathogen circulation remains a major challenge in tick‐borne disease ecology, particularly in biogeographically transitional regions. *Dermacentor reticulatus*, a widespread Palearctic tick species and vector of multiple pathogens, provides a useful system to investigate this relationship in a biogeographically complex landscape. Here, we combined mitochondrial (*cox1*) and nuclear (ITS2) population genetic analyses with broad‐spectrum pathogen screening in host‐seeking *D. reticulatus* from Anatolia, a major ecogeographical transition zone of the eastern Palearctic. Population genetic analyses revealed moderate haplotype diversity, low nucleotide divergence, and limited regional structuring, with Anatolian populations clustering within widespread eastern Palearctic lineages. Global haplotype networks indicated extensive haplotype sharing with Eurasian populations, consistent with historical connectivity and possible host‐mediated dispersal. In contrast to the relatively limited genetic structuring observed in tick populations, pathogen detections showed marked spatial heterogeneity. *Rickettsia raoultii* was detected predominantly in northeastern populations, whereas *Coxiella burnetii* detections were concentrated within a single locality. Other detected agents, including piroplasmids and *Anaplasma phagocytophilum*, occurred sporadically and showed no clear spatial pattern. Together, these findings suggest a mismatch between vector genetic structure and pathogen spatial distribution. Comparisons with sympatric *Dermacentor marginatus* populations further suggested species‐specific differences in population structure and pathogen assemblages. The observed spatial heterogeneity in pathogen detections may reflect differences in local ecological conditions, host communities, and transmission dynamics. These findings provide new insight into the eco‐epidemiological dynamics of *D. reticulatus*‐associated pathogens in Anatolia and contribute to improved understanding of tick‐borne pathogen (TBP) circulation at the southeastern margin of the Palearctic region.

## 1. Introduction

*Dermacentor reticulatus* (Fabricius, 1794) is a three‐host ixodid tick species widely distributed across the northern Palearctic region, including large parts of Europe and Asia. The species is primarily associated with open habitats such as grasslands, pastures, and ecotonal landscapes [1–3]. Immature stages feed mainly on small mammals, whereas adults parasitize a broad range of medium‐ and large‐sized mammals, including carnivores, ruminants, horses, and wild boars. Adult ticks exhibit exophilic behavior with seasonal activity peaks in spring and autumn, and occasional human infestations have also been reported [4–6].

The medical importance of *D. reticulatus* is closely associated with its role as a vector of numerous tick‐borne pathogens (TBPs). Confirmed vector competence has been demonstrated for several piroplasmids, including *Babesia canis*, *Babesia caballi*, and *Theileria equi*, as well as bacterial pathogens such as *Rickettsia raoultii*, *Rickettsia slovaca*, and *Anaplasma marginale*. In addition, the species has been implicated in the transmission cycles of viral agents including tick‐borne encephalitis virus (TBEV) and Omsk hemorrhagic fever virus [4, 7–9]. Its increasing epidemiological relevance in Europe has been highlighted particularly in relation to TBEV circulation [10, 11].

Over recent decades, *D. reticulatus* has increasingly been recognized as an expanding tick species in Europe, with newly established populations reported from previously nonendemic areas [8, 12–16]. Its expansion has been associated with environmental change, host availability, and considerable ecological plasticity, allowing the species to persist under diverse climatic and habitat conditions [3, 17, 18]. Parallel to its geographic expansion, increasing attention has been directed toward its role in the circulation of TBPs affecting humans and domestic animals, emphasizing the importance of continued surveillance and monitoring efforts [4, 19–22].

Despite increasing interest in *D. reticulatus*, important gaps remain regarding its population structure and pathogen associations across several parts of its range, including Türkiye. This limitation is particularly relevant in regions where *D. reticulatus* occurs sympatrically with other medically important tick species, such as *Ixodes ricinus* and *Dermacentor marginatus*. Shared hosts, habitats, and overlapping seasonal activity patterns among these species may contribute to heterogeneous pathogen circulation dynamics [8, 23]. Therefore, integrated studies combining vector population genetics with pathogen screening are needed to better understand the eco‐epidemiological structure of such systems.

Understanding the ecology of vector ticks and their associated pathogens is essential for improving surveillance and control strategies for TBDs [24]. In this context, population genetic analyses can provide valuable information on population connectivity and demographic structure that may influence pathogen distribution patterns across different spatial scales [25]. Combining population genetic data with pathogen screening from the same tick populations offers an integrative framework for exploring potential relationships between the vector structure and pathogen occurrence. Although such approaches remain relatively limited in ticks, recent studies have highlighted their value for understanding vector–pathogen systems [26–28].

Several population genetic studies have investigated *D. reticulatus* across Eurasia using different molecular markers [29–33]. However, studies integrating population genetic analyses with broad pathogen screening within the same *D. reticulatus* populations remain limited [34]. In Türkiye, no large‐scale study has yet examined the population genetic structure of *D. reticulatus* together with its associated TBPs. Therefore, the present study aimed to characterize the population genetic structure and TBP profile of *D. reticulatus* collected from natural foci located on both sides of the Anatolian Diagonal. As a secondary objective, the findings were evaluated in relation to previously published data on sympatric *D. marginatus* populations [27, 28] to provide a broader insight into tick population structure and pathogen circulation in Anatolia.

## 2. Materials and Methods

### 2.1. Study Area, Sampling Strategy, Specimen Collection, and Morphological Identification

Tick sampling for *D. reticulatus* was conducted at the same study sites previously established for a population genetic investigation of *D. marginatus* in Anatolia [27]. These sites were originally selected based on the known distribution of *D. marginatus* and stratified across two major ecogeographical regions of Türkiye: Central Anatolia (CN) and Northeastern Anatolia (NE), separated by the Anatolian Diagonal and characterized by distinct environmental features. The selection and ecological characterization of the 31 study sites (19 in CN and 12 in NE), based on the spatial use of domestic and wild animals, are described in detail in Orkun et al. [27].

Because adult *D. reticulatus* and *D. marginatus* are active during similar seasonal periods [8, 35], an opportunistic collection strategy was applied in which both species were collected whenever present. While all study sites had previously been confirmed to harbor *D. marginatus*, the presence and distribution of *D. reticulatus* were largely unknown prior to this study. Consequently, the sympatric occurrence of two species was observed at some sites, whereas *D. reticulatus* was absent from others.

Each study site comprised multiple pinpoint localities (i.e., discrete vegetation patches or microhabitats) where ticks were actively searched. In total, 382 pinpoint localities were surveyed across 31 study sites. From these surveys, adult *D. reticulatus* specimens were collected whenever present, yielding a total of 212 host‐seeking ticks that were used for pathogen screening and a subset of 160 specimens that were selected for population genetic analyses (Table 1). Specimens included in population genetic analyses were selected to maximize geographic representation across positive localities while minimizing the overrepresentation of densely sampled sites. To allow direct comparison with the previously established *D. marginatus* dataset [27], information on site identity, geographic region, and sampling effort was harmonized between the two studies, and the sympatric occurrence of both species at individual localities was recorded for subsequent comparative analyses.

**Table 1:** Summary of sampling localities and *Dermacentor reticulatus* specimens collected across Central and Northeastern Anatolia, including the number of surveyed localities, positive localities, specimens used for population genetic analyses, and the occurrence of mixed *D. reticulatus*–*D. marginatus* populations.

Study region	Study site	Number of localities surveyed	Number of localities positive for *D. reticulatus*	Total number of *D. reticulatus* specimens collected (♂/♀)	Number of specimens included in population genetic analyses (♂/♀)	Number of localities represented in population genetic analyses	Number of localities with mixed *D. reticulatus*–*D. marginatus* populations
CN	L1	14	9	77 (38♂, 39♀)	54 (26♂, 28♀)	9	9
CN	L2	13	5	31 (15♂, 16♀)	14 (8♂, 6♀)	5	3
CN	L3	12	5	13 (5♂, 8♀)	13 (5♂, 8♀)	5	2
CN	L4	12	1	1 (♂)	1 (♂)	1	1
CN	L5	12	0	0	0	0	0
CN	L6	14	0	0	0	0	0
CN	L7	12	5	6 (3♂, 3♀)	6 (3♂, 3♀)	5	5
CN	L8	14	0	0	0	0	0
CN	L9	13	0	0	0	0	0
CN	L10	15	0	0	0	0	0
CN	L11	17	0	0	0	0	0
CN	L12	11	0	0	0	0	0
CN	L13	10	0	0	0	0	0
CN	L14	10	0	0	0	0	0
CN	L15	11	0	0	0	0	0
CN	L15B	13	0	0	0	0	0
CN	LA	14	0	0	0	0	0
CN	LGr	15	4	12 (3♂, 9♀)	10 (3♂, 7♀)	4	2
CN	LM	19	0	0	0	0	0
NE	L16	11	4	9 (3♂, 6♀)	9 (3♂, 6♀)	4	3
NE	L17	10	2	3 (1♂, 2♀)	3 (1♂, 2♀)	2	2
NE	L18	13	4	7 (4♂, 3♀)	7 (4♂, 3♀)	4	2
NE	L19	11	0	0	0	0	0
NE	L20	10	1	3 (1♂, 2♀)	3 (1♂, 2♀)	1	1
NE	L21	13	5	21 (7♂, 14♀)	15 (5♂, 10♀)	5	4
NE	L22	8	2	20 (2♂, 18♀)	16 (72♂, 14♀)	2	2
NE	L23	10	0	0	0	0	0
NE	L24	13	0	0	0	0	0
NE	L25	8	0	0	0	0	0
NE	L26	11	3	9 (3♂, 6♀)	9 (3♂, 6♀)	3	2
NE	L27	13	0	0	0	0	0
TOTAL	31	382	50	212 (86♂, 126♀)	160 (65♂, 95♀)	50	38

Abbreviations: CN, Central Anatolia; NE, Northeast Anatolia.

Host‐seeking adult ticks were collected during the main seasonal activity periods of *Dermacentor* spp. (fall 2021, spring 2022, fall 2022, and spring 2023) using a standardized flagging and visual search protocol. A 1.5 × 1 m white cotton cloth was dragged over vegetation, and ticks were removed from the cloth or directly from the vegetation when observed. Each specimen was placed in an individually labeled air‐permeable tube and transported to the laboratory on the day of collection. Ticks from CN were transported to the Ticks and Tick‐borne Diseases Research Laboratory (TTBDRL) at Ankara University, Faculty of Veterinary Medicine, on the day of collection. Ticks from NE were initially transported to Kafkas University and subsequently transferred alive to the TTBDRL under controlled laboratory conditions.

All specimens were examined under a stereomicroscope (Stemi 2000‐C, Zeiss, Germany) and identified morphologically using standard taxonomic keys for *D. reticulatus* [35, 36]. After identification, ticks were washed in 70% ethanol, rinsed in sterile DNase/RNase‐free water, dried on sterile filter paper, and stored individually at −80 °C until molecular analyses.

**Figure 1:**
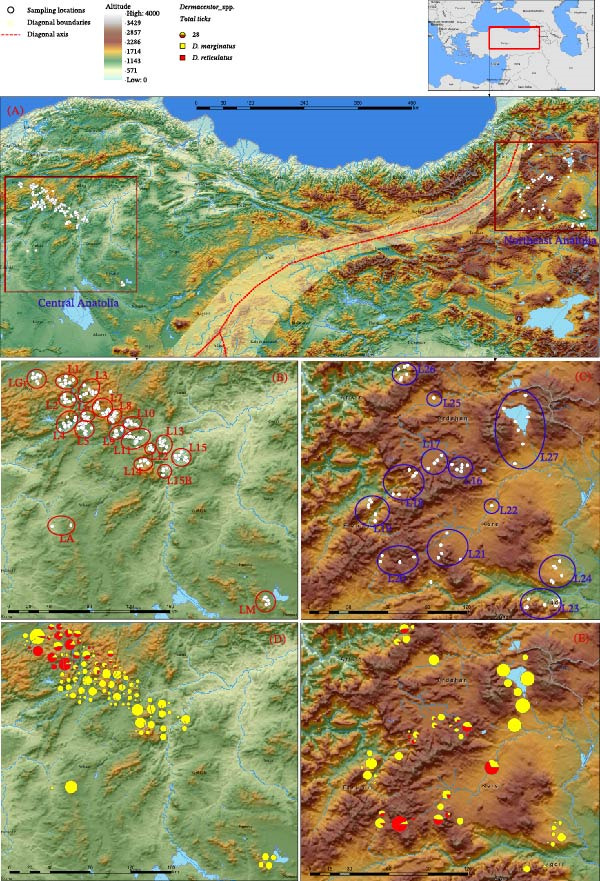
Geographic distribution of sampling sites and species composition of *Dermacentor* ticks in Anatolia. (A) Overview map of Central and Northeastern Anatolia showing all sampling localities included in this study. White circles indicate localities where *D. reticulatus* and/or *D. marginatus* were recorded. The Anatolian Diagonal is shown as a dashed red line (after Kuzguncuoğlu et al., 2019). (B, C) Regional enlargements of panel A displaying the predefined study sites used in the population genetic framework [28]: (B) Central Anatolia and (C) Northeastern Anatolia. Circles denote study sites comprising multiple sampling localities. (D, E) Species composition of collected *Dermacentor* ticks shown as proportional pie charts for each locality: (D) Central Anatolia and (E) Northeastern Anatolia. Red sectors represent *D. reticulatus* and yellow sectors represent *D. marginatus*; pie size corresponds to the number of collected ticks. Both single‐species and sympatric populations are present. *Dermacentor reticulatus* data derive from the present study, whereas the *D. marginatus* distribution and site framework follow Orkun et al. [28].

The geographic distribution of study sites and the occurrence of *D. reticulatus* and *D. marginatus* across Anatolia were mapped using ArcGIS 10.6.1 (Esri, Redlands, CA, USA) and are illustrated in Figure 1.

### 2.2. Molecular Analyses: Nucleic Acid Extraction, cDNA Synthesis, PCR, and Sequencing

Each *D. reticulatus* specimen was processed individually. Ticks were mechanically homogenized using a SpeedMill PLUS cooling homogenizer (Analytik Jena, Jena, Germany), and total genomic DNA and RNA were co‐extracted using the innuPREP Tick DNA/RNA Kit (IST Innuscreen GmbH, Berlin, Germany), following the manufacturer’s instructions. DNA extracts were stored at –20 °C, whereas RNA samples were preserved at –80 °C until further analyses. RNA extracts were reverse‐transcribed into complementary DNA (cDNA) using random hexamer primers and the Transcriptor High Fidelity cDNA Synthesis Kit (Roche, Mannheim, Germany), and cDNA was stored at –20°C.

To characterize tick genetic diversity, two independent PCR assays were performed for each specimen, targeting mitochondrial and nuclear markers. An ~850 bp fragment of the mitochondrial cytochrome c oxidase subunit 1 (*cox1*) gene was amplified using primers HCO2064 and HCO1215 [37], and the complete internal transcribed spacer 2 (ITS2) region (~1100 bp) was amplified using primers 3SA and JB9A [38] (Supporting Information 1: Table S1).

The same DNA and cDNA extracts were subsequently screened for a wide panel of TBPs using conventional, nested, or semi‐nested PCR assays. The screening included piroplasmids, *Rickettsia* spp., *Coxiella burnetii*, *Anaplasma* spp., *Borrelia burgdorferi* sensu lato, *Francisella* spp., *Ehrlichia* spp., *Hepatozoon* spp., and selected tick‐borne viruses including Crimean–Congo hemorrhagic fever virus, Aigai virus, and TBEV. Target genes, primers, cycling conditions, and references for all PCR assays are summarized in Supporting Information 1: Table S1 [39–63].

Each PCR run included sterile water as a negative control and known DNA or cDNA as positive controls, including *Anaplasma phagocytophilum*, *Anaplasma ovis*, *Babesia divergens*, *Borrelia afzelii*, *C. burnetii*, *Ehrlichia* sp., *Francisella tularensis*, *Hepatozoon felis*, *Rickettsia montanensis*, *Theileria annulata*, CCHFV, and TBEV, as appropriate for each target pathogen. Pilot PCR runs using serial dilutions of positive controls were performed to optimize amplification conditions for low‐copy‐number targets.

PCR products were purified using the ExoSAP‐IT PCR Product Cleanup Kit (Thermo Scientific, Santa Clara, CA, USA) and sequenced bidirectionally using the BigDye Terminator v3.1 Cycle Sequencing Kit (Thermo Scientific) on an ABI 3500 Genetic Analyzer. Raw chromatograms were edited and assembled into consensus sequences using AliView v1.26 [64], and sequence identity was confirmed by BLAST searches against the GenBank database.

### 2.3. Genetic Diversity, Population Structure, and Demographic History of *D. reticulatus*

To investigate the genetic diversity, population structure, and demographic history of *D. reticulatus*, three independent datasets were generated: (i) a mitochondrial *cox1* dataset, (ii) a nuclear ITS2 dataset, and (iii) a concatenated multilocus dataset combining *cox1* and ITS2 sequences for each individual. Mitochondrial *cox1* sequences were translated into amino acid sequences in AliView and screened for stop codons and potential nuclear mitochondrial pseudogenes (*numts*) using BLAST searches against the GenBank database. The *cox1* sequences were then aligned as translated amino acids using the MUSCLE algorithm [65] implemented in AliView. Nuclear ITS2 sequences were aligned using the Q‐INS‐i algorithm in MAFFT v7 [66], which incorporates RNA secondary structure information, with a scoring matrix of 200 PAM/k = 2 and a gap opening penalty of 1.53. The concatenated multilocus dataset was constructed by joining the aligned *cox1* and ITS2 sequences of each specimen.

Genetic diversity indices, including nucleotide diversity (*π*), number of haplotypes or genotypes (*h*), haplotype/genotype diversity (*Hd*), number of segregating sites (*S*), and mean number of pairwise nucleotide differences (*k*), were calculated for each dataset using DnaSP v6.12.03 [67]. Because some study sites contained very few individuals and preliminary analyses showed limited statistical power at the site level, populations were grouped at the regional scale into CN and NE. All analyses were therefore performed for CN and NE and for the pooled dataset (ALL). Haplotype and genotype relationships were examined by constructing statistical parsimony networks using TCS v1.21 [68], and the resulting networks were visualized using tcsBU [69]. To place Anatolian haplotypes/genotypes within a broader Palearctic context, additional global networks were constructed by combining sequences generated in this study with all available GenBank records of known geographic origin for *cox1* and ITS2. Alignments were trimmed to a common length to maximize overlap among regions, and statistical parsimony networks were inferred using the TCS algorithm implemented in PopArt v1.7 [70]. These global networks were used to visualize large‐scale haplotype/genotype sharing across Eurasia. To examine genetic similarity among haplotypes and genotypes, pairwise percent identity matrices were generated for the *cox1*, ITS2, and concatenated datasets. Unique haplotypes (*cox1*) and genotypes (ITS2 and concatenated) were aligned, and pairwise nucleotide sequence identities (%) were calculated for all pairs while excluding gap positions. These matrices were computed using custom scripts implemented in Python and visualized as triangular heatmaps to illustrate fine‐scale genetic similarity among regional and shared haplotypes/genotypes.

To quantify population genetic structure, analysis of molecular variance (AMOVA) and ΦST statistics were calculated in Arlequin v3.5.2.2 [71], using pairwise nucleotide differences as the distance measure and 1023 permutations to test for statistical significance. Pairwise ΦST values between CN and NE were also computed. Patterns of isolation by distance (IBD) were assessed using a Mantel test implemented in R v4.3.2 with the geodist, ape, and vegan packages [72, 73]. Pearson’s product–moment correlation coefficient (*r*) was calculated between matrices of pairwise genetic and geographic distances, and statistical significance was evaluated using 999 permutations.

Multivariate clustering was performed using discriminant analysis of principal components (DAPC) implemented in the adegenet package in R [74]. Both unsupervised clustering (using find.clusters) and supervised analyses based on predefined CN and NE population assignments were conducted. To infer the demographic history, mismatch distribution analyses were performed in DnaSP, and neutrality tests were applied to evaluate deviations from the neutral mutation hypothesis, including Tajima’s D, Fu’s Fs, and Fu and Li’s D and F statistics, all computed in DnaSP.

### 2.4. Phylogenetic Analysis

Phylogenetic analyses were conducted to determine the evolutionary relationships and geographic placement of *D. reticulatus* haplotypes/genotypes and to assess the phylogenetic placement of the detected TBPs. Separate phylogenetic datasets were constructed for mitochondrial *cox1*, nuclear ITS2, and pathogen markers (18S rRNA for piroplasmids and *groEL* for *A. phagocytophilum*).

For tick phylogenies, unique *cox1* haplotypes and ITS2 genotypes identified in this study were combined with homologous reference sequences of *D. reticulatus* retrieved from GenBank, representing diverse geographic regions across Eurasia. Two outgroup sequences (*D. marginatus* and *D. raskemensis*) were included to root the trees. For piroplasmids, 18S rRNA haplotypes obtained in this study were combined with published sequences of *Babesia* and *Theileria* species from GenBank using *Cardiosporidium cionae* as an outgroup. For *A. phagocytophilum*, partial *groEL* gene sequences were aligned with a curated dataset representing all major ecotypes and clusters described for this species.

Sequence reliability was assessed using GUIDANCE2 [75], and low‐confidence alignment columns were excluded to improve alignment quality. Non‐coding genes (ITS2 and 18S rRNA) were aligned using the Q‐INS‐i algorithm in MAFFT, whereas protein‐coding genes (*cox1* and *groEL*) were aligned as translated amino acids using the MUSCLE algorithm integrated into AliView. Alignment quality was additionally evaluated using *p*‐distance estimates in MEGA v11.0.13 [76].

Phylogenetic trees were inferred using Bayesian inference implemented in the BEAST package (BEAUti v2.7.6, BEAST v2.7.6, and TreeAnnotator v2.7.4) [77]. Substitution models were selected for each dataset based on model testing using jModelTest v2.1.10 [78]: HKY+I+G for *cox1*, TN93+I+G for 18S rRNA, and HKY+G for ITS2 and *groEL*. Markov Chain Monte Carlo (MCMC) analyses were run for 100 million generations, and convergence and effective sample sizes (ESS > 200) for all parameters were assessed in Tracer v1.7.2 [79]. Trees were sampled at regular intervals, and the first 20% were discarded as burn‐in. Maximum clade credibility trees were summarized using TreeAnnotator and visualized in FigTree v1.4.4 (Rambaut, A.; http://tree.bio.ed.ac.uk/software/figtree). Reference sequence accession numbers and geographic origins were annotated on the trees, and sequences generated in this study were highlighted. Phylogenetic analyses were used to complement BLAST‐based sequence identification and to assess the broader phylogenetic relationships of Anatolian *D. reticulatus* haplotypes and associated pathogens.

### 2.5. Mapping and Database Registration of Haplotypes and Genotypes of Ticks and Associated Pathogens

Spatial visualization of tick haplotypes/genotypes and associated pathogens was performed to illustrate their distribution across Anatolia. Separate distribution maps were generated for *D. reticulatus cox1* haplotypes, ITS2 genotypes, and detected TBPs. Mapping was conducted using ArcGIS based on the geographic coordinates recorded for each sampling locality. Haplotypes, genotypes, and pathogen‐positive samples were overlaid onto regional maps of CN and NE.

All unique tick haplotypes and genotypes identified in this study were deposited in the GenBank database. Accession numbers for *D. reticulatus cox1* haplotypes range from PX789633–PX789648, and those for ITS2 genotypes range from PX789865–PX789891. In addition, all *D. reticulatus cox1* haplotypes were registered in the Barcode of Life Data Systems (BOLD) database together with associated specimen metadata to facilitate future DNA barcoding and comparative studies.

Pathogen‐derived haplotypes were also submitted to GenBank. These included *Babesia occultans* (18S rRNA; PX788778), *B. canis* (18S rRNA; PX788777; mitochondrial *cytb*; PX802885), *Babesia vulpes* (18S rRNA; PX788779), *Theileria ovis* (18S rRNA; PX788780), *Coxiella burnetii* (IS1111‐Tnp; PX802889), *R. raoultii* (*ompA*; PX802887), *R. slovaca* (*ompA*; PX802888), and *A. phagocytophilum* (*groEL*; PX802886).

## 3. Results

### 3.1. Tick Sampling and Geographical Distribution

A total of 382 individual localities grouped into 31 predefined study sites were surveyed across CN and NE. *Dermacentor reticulatus* was detected in 13 study sites, including 6/19 in CN and 7/12 in NE, and was collected from 50 individual localities, yielding a total of 212 adult ticks (Table 1). Sampling success varied among localities, with the number of collected specimens ranging from one to multiple individuals per locality (mean = 4.24 specimens per positive locality). In CN, *D. reticulatus* was detected in approximately one‐third of the surveyed study sites, whereas in NE, it was detected in more than half of the sites examined. Mixed populations of *D. reticulatus* and *D. marginatus* were observed in 38 of the 50 positive localities. For population genetic analyses, 160 adult *D. reticulatus* specimens were selected to ensure representation from all positive localities, whereas all 212 specimens were included in pathogen screening analyses. The geographic distribution of surveyed localities and *D. reticulatus* occurrences is shown in Figure 1, and detailed sampling information is provided in Table 1.

### 3.2. Population Genetic Structure of *D. reticulatus*

The population genetic structure of *D. reticulatus* was investigated using mitochondrial (*cox1*), nuclear (ITS2), and concatenated multilocus datasets. A total of 160 adult specimens representing all positive localities were included in the analyses and assigned to CN or NE according to geographic origin. Genetic diversity, population differentiation, clustering, and isolation‐by‐distance patterns were evaluated separately for each dataset. Because several study site clusters contained small and uneven sample sizes, downstream population genetic analyses were performed using region‐based grouping (CN vs NE), together with analyses of the pooled dataset (ALL).

#### 3.2.1. Mitochondrial cox1 Diversity and Population Structure

Mitochondrial *cox1* variation was analyzed in *D. reticulatus* populations from CN, NE, and the pooled dataset (ALL). A total of 16 haplotypes defined by 15 segregating sites were identified, with moderate haplotype diversity and low nucleotide diversity across Anatolian populations (*Hd* = 0.532; *π* = 0.00078) (Table 2). Haplotype diversity was lower in CN (*Hd* = 0.38) than in NE (*Hd* = 0.633), whereas nucleotide diversity remained low in both regions (*π* = 0.00055 in CN; *π* = 0.00092 in NE). Neutrality test statistics were predominantly negative across datasets. Significant negative values of Tajima’s D and Fu and Li’s statistics were observed for the pooled dataset, whereas neutrality statistics for NE were not statistically significant (Table 2). Mismatch distribution analyses showed unimodal distributions across datasets (Supporting Information 13: Figure S1).

**Table 2:** Mitochondrial *cox1* gene–based genetic diversity and neutrality test results of Anatolian populations of *Dermacentor reticulatus*.

Population	*n*	*h*	*Hd*	*π*	*S*	*k*	Fu’s Fs	Tajima’s D	Fu and Li’s D	Fu and Li’s F
CN	98	11	0.38	0.00055	10	0.456	−10.841	−1.9603 ^∗^	−2.13187	−2.45965 ^∗^
NE	62	7	0.633	0.00092	6	0.766	−2.669	−0.97717	−1.51718	−1.57865
ALL	160	16	0.532	0.00078	15	0.65	−15.494	−1.97121 ^∗^	−2.55361 ^∗^	−2.795442 ^∗^

*Note:* Summary statistics are presented for Central Anatolia (CN), Northeastern Anatolia (NE), and the pooled dataset (ALL), including number of haplotypes, polymorphic sites, haplotype diversity, nucleotide diversity, mean number of pairwise differences, and neutrality test statistics (Tajima’s D, Fu and Li’s D and F, and Fu’s Fs). *π*, nucleotide diversity; *h*, number of haplotypes; *n*, number of individuals; *S*, number of segregation sites; *k*, average number of nucleotide differences.

Abbreviation: *Hd*, haplotype diversity.

^∗^Statistically significant (*p*  < 0.05).

**Figure 2:**
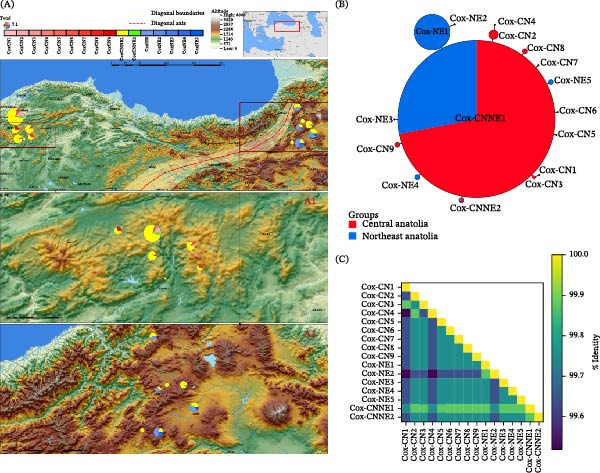
Mitochondrial *cox1* haplotype structure of *Dermacentor reticulatus* (*n* = 160) from Anatolia. (A) Geographic distribution of haplotypes across sampling localities. The upper panel shows the overall study area, with enlargements of (A1) Central Anatolia and (A2) Northeastern Anatolia. Pie charts represent haplotype composition at each site. Colors denote regional affiliation: Central Anatolia (red shades), Northeastern Anatolia (blue shades), and shared haplotypes (yellow–green shades). (B) TCS haplotype network inferred from *cox1* sequences. Circle size is proportional to haplotype frequency, and each connecting line represents a single mutational step unless otherwise indicated. Colors correspond to geographic origin (Central Anatolia = red; Northeastern Anatolia = blue). (C) Pairwise nucleotide identity matrix among haplotypes based on *cox1* sequences, shown as a color‐coded heatmap.

The *cox1* haplotype network showed a compact star‐like topology centered around two regionally shared haplotypes (Cox‐CNNE1 and Cox‐CNNE2), which together comprised 110/160 specimens (68.8%) (Figure 2). Several low‐frequency haplotypes were restricted to either CN or NE and differed from the central haplotypes by one or a few mutational steps. No deeply divergent haplogroups were observed.

AMOVA based on *cox1* sequences showed that 78.7% of the total genetic variation occurred within populations, whereas 21.3% was attributable to differences between CN and NE populations (ΦST = 0.213; *p*  < 0.001). Pairwise ΦST values between CN and NE were also significant (ΦST = 0.213; *p*  < 0.001) (Supporting Information 2: Table S2). Mantel tests revealed a weak but significant positive correlation between genetic and geographic distances (*r* = 0.162, *p* = 0.001; 999 permutations).

**Figure 3:**
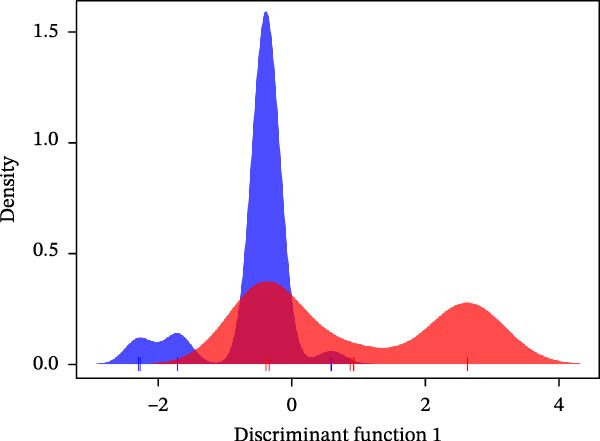
Supervised discriminant analysis of principal components (DAPC) based on mitochondrial *cox1* sequences of *Dermacentor reticulatus*. Individuals were assigned a priori to Central Anatolia (CN) and Northeastern Anatolia (NE) populations. The density plot along the first discriminant function shows partial separation between regions with substantial overlap, indicating moderate mitochondrial population structure.

DAPC analyses based on *cox1* variation identified shallow population structuring. Unsupervised analyses recovered two partially overlapping genetic clusters, whereas supervised analyses based on predefined CN and NE assignments showed partial regional separation (Figure 3; Supporting Information 14: Figure S2).

#### 3.2.2. Nuclear ITS2 Diversity and Population Structure

Nuclear ITS2 variation was analyzed in the same *D. reticulatus* specimens used for mitochondrial analyses. Overall, ITS2 data showed low genetic variability, with limited genotype diversity and low nucleotide diversity across CN, NE, and the pooled dataset (Supporting Information 3: Table S3). Neutrality test statistics were generally nonsignificant across datasets. Mismatch distribution analyses showed unimodal distributions (Supporting Information 15: Figure S3).

The ITS2 genotype network showed extensive sharing of common genotypes between the CN and NE populations and no deeply divergent lineages. Common genotypes were shared between CN and NE populations and together accounted for 79.4% (127/160) of the analyzed specimens (Supporting Information 16: Figure S4). AMOVA indicated that most genetic variation occurred within populations, whereas variation between regions was low and nonsignificant (Supporting Information 4: Table S4). Mantel tests did not reveal a significant correlation between genetic and geographic distances (*r* = 0.01382, *p* = 0.139; 999 permutations). Similarly, supervised and unsupervised DAPC analyses did not recover distinct geographic clusters (Supporting Information 17: Figure S5 and Supporting Information 18: Figure S6).

#### 3.2.3. Multilocus (*cox1*+ITS2) Population Genetic Structure

Multilocus analyses based on concatenated *cox1* and ITS2 sequences were performed to evaluate combined genetic patterns in Anatolian *D. reticulatus* populations. The concatenated dataset contained 58 multilocus genotypes and showed high genotype diversity together with low nucleotide diversity across regional and pooled datasets (Supporting Information 5: Table S5). Neutrality test statistics were predominantly negative, with Fu’s Fs showing significant deviations from neutrality in the pooled dataset and CN (Supporting Information 5: Table S5). Mismatch distribution analyses showed unimodal patterns across datasets (Supporting Information 19: Figure S7).

Multilocus genotype networks showed sharing of common genotypes between CN and NE populations, with no deeply divergent lineages observed (Supporting Information 20: Figure S8). AMOVA indicated that most genetic variation occurred within populations, whereas a smaller but significant proportion was attributable to regional differences (ΦST = 0.07268; *p*  < 0.001; Supporting Information 6: Table S6). Mantel tests revealed a weak but significant correlation between genetic and geographic distances (*r* = 0.08337, *p*  < 0.01; 999 permutations). Supervised and unsupervised DAPC analyses showed partial overlap among inferred groups (Supporting Information 21: Figure S9 and Supporting Information 22: Figure S10).

#### 3.2.4. Sequence Similarity, Phylogenetic Placement, and Global Network Context of *cox1* and ITS2 Haplotypes/Genotypes

Sequence similarity and phylogenetic placement of mitochondrial *cox1* haplotypes and nuclear ITS2 genotypes from Anatolian *D. reticulatus* were evaluated using BLAST analyses and Bayesian phylogenetic inference with representative GenBank sequences.

For the *cox1* gene, 16 haplotypes were identified, including nine restricted to CN, five restricted to NE, and two shared between regions (Supporting Information 7: Table S7). Except for Cox‐CNNE1 and Cox‐CN8, none of the haplotypes showed identical matches in GenBank, indicating that most represent previously unreported variants. The highest sequence similarity (99.76%–100%) was observed with *D. reticulatus* sequences from the Stavropol region of Russia. Bayesian phylogenetic analysis based on *cox1* sequences included the 16 haplotypes identified in this study, together with representative GenBank sequences. Anatolian haplotypes clustered within a major clade containing *D. reticulatus* sequences from multiple European and Eurasian countries, whereas a second smaller clade consisted exclusively of Croatian sequences (Supporting Information 23: Figure S11a and Supporting Information 8: Table S8).

A global *cox1* haplotype network was constructed by combining 160 Anatolian *D. reticulatus* sequences with GenBank records of known geographic origin (total *n* = 260). After trimming sequences to a common length of 606 bp, the dataset contained 32 haplotypes overall, including 13 from Anatolia. The most common haplotype (HP1) was shared between Anatolia and Russia, whereas another widespread haplotype (HP2) linked CN with Russia and Kazakhstan. A third common haplotype (HP3) included sequences from several European countries and Russia. In addition, several haplotypes were restricted to CN or NE populations within Türkiye (Supporting Information 24: Figure S12; Supporting Information 9: Table S9). Overall, Anatolian haplotypes clustered within widely distributed Palearctic *cox1* lineages.

For the nuclear ITS2 marker, 27 genotypes were identified, including nine restricted to CN, seven restricted to NE, and 11 shared between regions (Supporting Information 10: Table S10). None of the ITS2 genotypes showed identical matches in GenBank, indicating that all represent previously unreported variants. The highest sequence similarity (99.54%–99.91%) was observed with *D. reticulatus* sequences from Poland. Bayesian phylogenetic analyses based on ITS2 sequences included the 27 genotypes identified in this study, together with representative GenBank reference sequences. Anatolian genotypes were distributed across two major clades together with sequences from several European and Eurasian countries, including Poland, Germany, the Czech Republic, Kazakhstan, and Russia (Supporting Information 23: Figure S11b and Supporting Information 11: Table S11).

A global ITS2 genotype network was constructed by combining 160 Anatolian *D. reticulatus* sequences with GenBank ITS2 records of known geographic origin (total *n* = 177). After trimming sequences to a common length of 591 bp, the dataset contained nine genotypes overall, including seven from Anatolia. The most frequent genotype (GN1) was shared across CN and NE and included reference sequences from Russia, Kazakhstan, Poland, the Czech Republic, and Portugal. Several genotypes were restricted to CN or NE populations within Türkiye, whereas others were limited to specific European regions (Supporting Information 25: Figure S13; Supporting Information 12: Table S12). Overall, Anatolian ITS2 genotypes clustered together with previously reported Eurasian genotypes.

Taken together, analyses based on both *cox1* and ITS2 markers identified multiple novel haplotypes/genotypes in Anatolian *D. reticulatus* populations while showing broad similarity to previously reported Eurasian sequences.

### 3.3. Detection and Distribution of TBPs

#### 3.3.1. Overall PCR Screening Results

**Figure 4:**
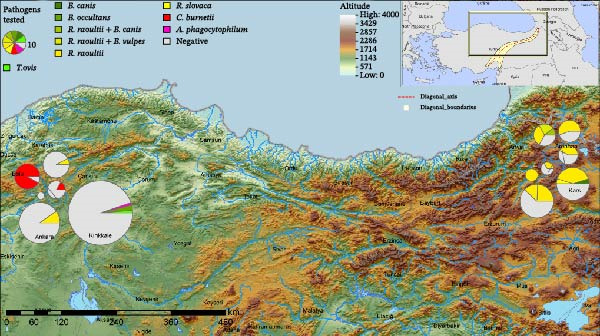
Spatial distribution of tick‐borne pathogens detected in *Dermacentor reticulatus* (*n* = 212) across Anatolia. Sampling localities are shown as proportional pie charts, where pie size reflects the number of examined ticks and colored sectors represent pathogen composition (see legend). Gray sectors indicate negative samples. A reference pie illustrates the numerical scale (one segment = 10 ticks). The inset map shows the position of the study area and the Anatolian Diagonal. Scale bar (km) and altitude color ramp are shown on the main map.

A total of 212 adult *D. reticulatus* specimens (86♂, 126♀) were screened for TBPs using PCR‐based assays. Overall, 56 ticks (26.4%) were positive for at least one pathogen, whereas three individuals (1.4%) harbored mixed infections involving two pathogens (Table 3; Figure 4). Five pathogen groups were detected, including *Rickettsia* spp. (*n* = 39), *Coxiella* spp. (*n* = 12), *Babesia* spp. (*n* = 6), *Theileria* sp. (*n* = 1), and *Anaplasma* sp. (*n* = 1). Mixed infections were observed only in three *Babesia*‐positive ticks, all co‐infected with *Rickettsia* spp. (Table 3). No PCR products were obtained for *Cytauxzoon* spp., *Theileria equi*, *Hepatozoon* spp., *Borrelia burgdorferi* sensu lato, *Borrelia miyamotoi*, *Anaplasma marginale/ovis*, *Ehrlichia* spp., *Francisella* spp., CCHFV, Aigai virus, or TBEV.

**Table 3:** Pathogens detected in *Dermacentor reticulatus* specimens in Anatolia.

Pathogens	Sample code	Gender	Region	Location code	Location (district/province)	Detected pathogen	Sequenced gene region	Haplotype	GenBank accession number
*Babesia* spp.	Dr141	Female	CN	L1.4	Kızılcahamam/Ankara	*B. occultans*	18S rRNA	BoTR‐DrHp1	PX788778
	Dr143	Male	CN	L1.4	Kızılcahamam/Ankara	*B. occultans*	18S rRNA	BoTR‐DrHp1	PX788778
	Dr2221	Female	NE	L26.2	Selim/Kars	*B. canis*	18S rRNA + *cytb*	BcTR‐DrHp1	PX788777/PX802885
	Dr2651	Male	NE	L26.5	Şavşat/Artvin	*B. canis* ^a^	18S rRNA + *cytb*	BcTR‐DrHp1	PX788777/PX802885
	Dr2653	Female	NE	L26.5	Şavşat/Artvin	*B. vulpes* ^a^	18S rRNA	BvTR‐DrHp1	PX788779
	Dr2653	Male	NE	L26.5	Şavşat/Artvin	*B. vulpes* ^a^	18S rRNA	BvTR‐DrHp1	PX788779
*Theileria* spp.	Dr185	Male	CN	L1.8	Kızılcahamam/Ankara	*T. ovis*	18S rRNA	ToTR‐DrHp1	PX788780
*Coxiella* spp.	Dr7121	Female	CN	L7.12	Çubuk/Ankara	*C. burnetii*	IS1111‐Tnp	CbTR‐DrHp1	PX802889
	DmGr‐H12	Male	CN	LGr‐H1	Gerede/Bolu	*C. burnetii*	IS1111‐Tnp	CbTR‐DrHp1	PX802889
	DmGr‐M11	Female	CN	LGr‐M1	Gerede/Bolu	*C. burnetii*	IS1111‐Tnp	CbTR‐DrHp1	PX802889
	DmGr‐M12	Female	CN	LGr‐M1	Gerede/Bolu	*C. burnetii*	IS1111‐Tnp	CbTR‐DrHp1	PX802889
	DmGr‐T16	Male	CN	LGr‐T1	Gerede/Bolu	*C. burnetii*	IS1111‐Tnp	CbTR‐DrHp1	PX802889
	DmGr‐T18	Female	CN	LGr‐T1	Gerede/Bolu	*C. burnetii*	IS1111‐Tnp	CbTR‐DrHp1	PX802889
	DmGr‐T19	Female	CN	LGr‐T1	Gerede/Bolu	*C. burnetii*	IS1111‐Tnp	CbTR‐DrHp1	PX802889
	DmGr‐T110	Male	CN	LGr‐T1	Gerede/Bolu	*C. burnetii*	IS1111‐Tnp	CbTR‐DrHp1	PX802889
	DmGr‐Th1	Female	CN	LGr‐T1	Gerede/Bolu	*C. burnetii*	IS1111‐Tnp	CbTR‐DrHp1	PX802889
	DmGr‐Th2	Female	CN	LGr‐T1	Gerede/Bolu	*C. burnetii*	IS1111‐Tnp	CbTR‐DrHp1	PX802889
	DmGr‐Y11	Female	CN	LGr‐Y1	Gerede/Bolu	*C. burnetii*	IS1111‐Tnp	CbTR‐DrHp1	PX802889
	DmGr‐Y12	Female	CN	LGr‐Y1	Gerede/Bolu	*C. burnetii*	IS1111‐Tnp	CbTR‐DrHp1	PX802889
*Rickettsia* spp.	Dr251	Male	CN	L2.5	Çamlıdere/Ankara	*R. raoultii*	ompA	RrTR‐DrHp1	PX802887
	Dr341	Female	CN	L3.5	Kızılcahamam/Ankara	*R. raoultii*	ompA	RrTR‐DrHp1	PX802887
	Dr2722	Male	CN	L2.7	Çamlıdere/Ankara	*R. raoultii*	ompA	RrTR‐DrHp1	PX802887
	Dr27h1	Male	CN	L2.7	Çamlıdere/Ankara	*R. raoultii*	ompA	RrTR‐DrHp1	PX802887
	Dr1611	Female	NE	L16.1	Central/Kars	*R. raoultii*	ompA	RrTR‐DrHp1	PX802887
	Dr1612	Male	NE	L16.1	Central/Kars	*R. raoultii*	ompA	RrTR‐DrHp1	PX802887
	Dr1616	Male	NE	L16.1	Central/Kars	*R. raoultii*	ompA	RrTR‐DrHp1	PX802887
	Dr1631	Female	NE	L16.3	Göle/Ardahan	*R. raoultii*	ompA	RrTR‐DrHp1	PX802887
	Dr1661	Female	NE	L16.6	Göle/Ardahan	*R. raoultii*	ompA	RrTR‐DrHp1	PX802887
	Dr1721	Female	NE	L17.2	Göle/Ardahan	*R. raoultii*	ompA	RrTR‐DrHp1	PX802887
	Dr1821	Male	NE	L18.2	Şenkaya/Erzurum	*R. raoultii*	ompA	RrTR‐DrHp1	PX802887
	Dr1831	Male	NE	L18.3	Şenkaya/Erzurum	*R. raoultii*	ompA	RrTR‐DrHp1	PX802887
	Dr1853	Male	NE	L18.4	Şenkaya/Erzurum	*R. raoultii*	ompA	RrTR‐DrHp1	PX802887
	Dr2041	Female	NE	L20.4	Şenkaya/Erzurum	*R. raoultii*	ompA	RrTR‐DrHp1	PX802887
	Dr2042	Female	NE	L20.4	Şenkaya/Erzurum	*R. raoultii*	ompA	RrTR‐DrHp1	PX802887
	Dr2043	Male	NE	L20.4	Şenkaya/Erzurum	*R. raoultii*	ompA	RrTR‐DrHp1	PX802887
	Dr2121	Female	NE	L21.2	Sarıkamış/Kars	*R. raoultii*	ompA	RrTR‐DrHp1	PX802887
	Dr21211	Female	NE	L21.2	Sarıkamış/Kars	*R. raoultii*	ompA	RrTR‐DrHp1	PX802887
	Dr21212	Female	NE	L21.2	Sarıkamış/Kars	*R. raoultii*	ompA	RrTR‐DrHp1	PX802887
	Dr21h3	Male	NE	L21.2	Sarıkamış/Kars	*R. raoultii*	ompA	RrTR‐DrHp1	PX802887
	Dr21h4	Male	NE	L21.2	Sarıkamış/Kars	*R. raoultii*	ompA	RrTR‐DrHp1	PX802887
	Dr2131	Female	NE	L21.3	Sarıkamış/Kars	*R. raoultii*	ompA	RrTR‐DrHp1	PX802887
	Dr2162	Female	NE	L21.6	Sarıkamış/Kars	*R. raoultii*	ompA	RrTR‐DrHp1	PX802887
	Dr21h5	Female	NE	L21.6	Sarıkamış/Kars	*R. raoultii*	ompA	RrTR‐DrHp1	PX802887
	Dr2211	Female	NE	L22.1	Sarıkamış/Kars	*R. raoultii*	ompA	RrTR‐DrHp1	PX802887
	Dr2214	Female	NE	L22.1	Central/Kars	*R. raoultii*	ompA	RrTR‐DrHp1	PX802887
	Dr2215	Female	NE	L22.1	Central/Kars	*R. raoultii*	ompA	RrTR‐DrHp1	PX802887
	Dr2217	Female	NE	L22.1	Central/Kars	*R. raoultii*	ompA	RrTR‐DrHp1	PX802887
	Dr2219	Female	NE	L22.1	Central/Kars	*R. raoultii*	ompA	RrTR‐DrHp1	PX802887
	Dr22111	Female	NE	L22.1	Central/Kars	*R. raoultii*	ompA	RrTR‐DrHp1	PX802887
	Dr22112	Female	NE	L22.1	Central/Kars	*R. raoultii*	ompA	RrTR‐DrHp1	PX802887
	Dr22116	Female	NE	L22.1	Central/Kars	*R. raoultii*	ompA	RrTR‐DrHp1	PX802887
	Dr22h1	Female	NE	L22.1	Central/Kars	*R. slovaca*	ompA	RsTR‐DrHp1	PX802888
	Dr2621	Male	NE	L26.2	Şavşat/Artvin	*R. raoultii*	ompA	RrTR‐DrHp1	PX802887
	Dr2651	Male	NE	L26.5	Şavşat/Artvin	*R. raoultii* ^b^	ompA	RrTR‐DrHp1	PX802887
	Dr2653	Female	NE	L26.5	Şavşat/Artvin	*R. raoultii* ^c^	ompA	RrTR‐DrHp1	PX802887
	Dr2654	Male	NE	L26.5	Şavşat/Artvin	*R. raoultii* ^c^	ompA	RrTR‐DrHp1	PX802887
	Dr2655	Female	NE	L26.5	Şavşat/Artvin	*R. raoultii*	ompA	RrTR‐DrHp1	PX802887
	Dr2657	Female	NE	L26.5	Şavşat/Artvin	*R. raoultii*	ompA	RrTR‐DrHp1	PX802887
*Anaplasma* spp.	Dr138	Female	CN	L1.3	Kızılcahamam/Ankara	*A. phagocytophilum*	*groEL*	ApTR‐DrHp1	PX802886

Abbreviations: CN, Central Anatolia; NE, Northeastern Anatolia.

^a^This sample was found as mixed with *R. raoultii*.

^b^This sample was found as mixed with *B. canis*.

^c^This sample was found as mixed with *B. vulpes*.

#### 3.3.2. Piroplasmids (*Babesia and Theileria* spp.)

PCR screening targeting the 18S rRNA gene detected piroplasmid infections in seven *D. reticulatus* specimens, including six *Babesia*‐positive and one *Theileria*‐positive tick. *Babesia* infections were detected in both CN and NE, whereas *T. ovis* was identified only in CN (Table 3; Figure 4).

*Babesia occultans* was detected in two ticks collected from the same study site (L3) in CN. The corresponding 18S rRNA sequences were identical and formed a single haplotype (BoTR‐DrHp1), showing complete identity with previously reported sequences from Türkiye derived from *Hyalomma* spp. and *D. marginatus* (e.g., OM066159 and PP463880). *Babesia canis* was identified in two ticks from NE (L22 and L26). The corresponding 18S rRNA sequences were identical and formed a single haplotype (BcTR‐DrHp1), showing complete identity with previously reported sequences from dogs in Türkiye and Europe (e.g., KF499115 and KT008057). Additional mitochondrial sequencing generated identical sequences that matched a previously reported Croatian dog‐derived isolate (KC207822). One *B. canis*‐positive tick was co‐infected with *R. raoultii*. *Babesia vulpes* was detected in two ticks collected from the same study site (L26) in NE. The corresponding sequences formed a single haplotype (BvTR‐DrHp1), which matched a previously reported sequence from host‐seeking *D. marginatus* in Türkiye (PP463884). Both *B. vulpes*‐positive ticks were co‐infected with *R. raoultii*.

A single tick collected from L3 in CN tested positive for *T. ovis*. The obtained 18S rRNA sequence showed 99.6% similarity to previously reported sequences from wild sheep in Türkiye (KT851429 and KT851427) and domestic sheep in China (FJ603460).

Bayesian phylogenetic analyses based on 18S rRNA sequences showed that all detected piroplasmid sequences clustered within their respective species‐specific clades (Supporting Information 26: Figure S14). *Babesia canis*, *B. occultans*, *B. vulpes*, and *T. ovis* each formed well‐supported monophyletic groups together with previously reported reference sequences from Europe, Asia, and Africa. Within these clades, the sequences identified in this study grouped most closely with previously reported isolates from Türkiye and neighboring Eurasian regions.

Except for the single *T. ovis*‐positive specimen, all piroplasmid‐positive ticks were included in population genetic analyses. Positive ticks were distributed among both shared and region‐specific *cox1* haplotypes and ITS2 genotypes without an obvious association with particular genetic groups.

#### 3.3.3. *Rickettsia* spp.

PCR screening targeting the *gltA* gene detected *Rickettsia*‐positive amplicons in 39 *D. reticulatus* specimens. Subsequent *ompA* sequencing identified 38 samples as *R. raoultii* and one sample as *R. slovaca* (Table 3). *Rickettsia raoultii* was detected in both CN and NE but occurred predominantly in NE, where 34 of 38 positive ticks were identified. In contrast, only four positive ticks were detected in CN. The single *R. slovaca*‐positive tick was collected from L22 in NE (Table 3; Figure 4).

Sequence analysis of the *ompA* gene showed that all *R. raoultii*‐positive samples shared a single haplotype (RrTR‐DrHp1), which was identical to previously reported sequences from *D. marginatus* and *D. reticulatus* in Türkiye and Europe (e.g., PP465040 and MF166729). Similarly, the *R. slovaca* haplotype (RsTR‐DrHp1) showed complete identity with previously reported sequences from *D. marginatus* in Türkiye and Italy (e.g., MF379303 and HM161793). Mixed infections involving *R. raoultii* together with *B. canis* or *B. vulpes* were detected in three ticks (see Section 3.3.2). Because no sequence variation was detected, additional phylogenetic analyses were not performed.

Among the *R. raoultii*‐positive ticks, 32 of 38 samples were included in population genetic analyses, whereas the single *R. slovaca*‐positive tick was not included. *Rickettsia raoultii*‐positive ticks were detected in both shared and NE‐specific *cox1* haplotypes, whereas CN‐specific haplotypes were represented by only two infected specimens. At the ITS2 level, infected ticks were associated only with shared or NE‐specific genotypes. Overall, *R. raoultii*‐positive ticks were detected predominantly in the NE populations of *D. reticulatus*.

#### 3.3.4. *C. burnetii*

PCR screening targeting the IS1111 transposase gene detected *Coxiella*‐positive amplicons in 12 *D. reticulatus* specimens. Sequence analyses identified all positive samples as *C. burnetii*, sharing a single haplotype designated as CbTR‐DrHp1. All positive ticks originated from CN and were collected from two study sites, including LGr (*n* = 11) and L7 (*n* = 1) (Table 3; Figure 4).

BLAST analysis of the IS1111‐Tnp sequence showed complete identity with previously reported *C. burnetii* sequences from diverse hosts and geographic regions, including *D. marginatus* in Türkiye, livestock in Asia, humans, and *Dermacentor andersoni* from North America (e.g., PP465023, MT920360, and CP040059). Because no sequence variation was detected among the obtained sequences, additional phylogenetic analyses were not performed.

Of the 12 *C. burnetii*‐positive ticks, 10 were included in population genetic analyses. At the *cox1* level, eight ticks belonged to the interregional haplotype Cox‐CNNE1, whereas two carried the CN‐specific haplotype Cox‐CN9. At the ITS2 level, most positive ticks were associated with interregional genotypes, whereas one specimen carried a CN‐specific genotype (Its‐CN8). All *C. burnetii*‐positive ticks were detected in CN populations, with 11 of the 12 positives originating from the LGr study site.

#### 3.3.5. *Anaplasma* spp.

PCR screening targeting the *msp4* gene detected a single *A. phagocytophilum*‐positive tick among the 212 examined *D. reticulatus* specimens. The positive specimen originated from CN and was collected at study site L1 (Table 3; Figure 4).

To further characterize the detected *A. phagocytophilum* variant, a partial *groEL* fragment was amplified and sequenced. BLAST analysis showed no identical match in GenBank, with the closest sequences displaying 99.8% identity to isolates previously reported from *I. ricinus* in Türkiye and Europe (e.g., OM127910 and HM057230).

Bayesian phylogenetic analysis based on *groEL* sequences placed the detected *A. phagocytophilum* variant within Ecotype I, specifically grouping in Cluster 1 together with previously reported sequences from different hosts and geographic regions (Supporting Information 27: Figure S15).

The *A. phagocytophilum*‐positive tick belonged to the interregional mitochondrial haplotype Cox‐CNNE2 and the CN‐specific ITS2 genotype Its‐CN4.

## 4. Discussion

### 4.1. *D. reticulatus* in Anatolia: Current Knowledge, Sympatry, and Expansion Potential

*D. reticulatus* is recognized as an epidemiologically important tick species in the Palearctic region; however, its ecology, population structure, and pathogen associations remain poorly characterized in the eastern part of its range, including Anatolia [1, 4, 8, 23]. This gap is particularly relevant because Anatolia represents a major biogeographic transition zone shaped by strong climatic gradients and geographic barriers such as the Anatolian Diagonal. The present study provides the first integrated assessment of *D. reticulatus* population genetic structure and TBP diversity in Anatolia while also documenting its regional distribution and sympatric associations.

In much of Europe, *D. reticulatus* commonly occurs sympatrically with *I. ricinus* and shares overlapping habitats and hosts [4, 8, 18, 23]. In contrast, *I. ricinus* was absent from most Anatolian sites where *D. reticulatus* was detected. Instead, *D. reticulatus* occurred predominantly together with *D. marginatus* and several *Haemaphysalis* species (*H. parva*, *H. punctata*, and *H. sulcata*), indicating a distinct tick assemblage compared with that in many European regions.

Across most study sites, *D. marginatus* was more prevalent than *D. reticulatus*, although several localities showed the opposite pattern. In the semi‐arid regions of CN, *D. reticulatus* was absent from multiple sites where only *D. marginatus* occurred, whereas in the cooler and more humid NE region, both species frequently co‐occurred. These findings indicate a heterogeneous distribution pattern of *D. reticulatus* across Anatolia. Although the current distribution limits of *D. reticulatus* in Türkiye remain incompletely defined, long‐term observations from CN ([80], this study) may suggest a gradual southward expansion into drier regions. Similar expansion trends have been reported from several parts of Europe [19, 81]. Continued monitoring will therefore be important for evaluating future changes in the distribution of the species in Anatolia.

In addition to habitats commonly reported from Europe [1–3], *D. reticulatus* in Anatolia was frequently detected along the margins of open agricultural landscapes dominated by dry farming practices, particularly wheat and barley cultivation. These habitats were especially common in transitional and semi‐arid parts of CN and were generally not associated with forested environments. The observed habitat use suggests that *D. reticulatus* can persist in open agroecosystems under relatively dry conditions in Anatolia.

### 4.2. Population Genetic Structure of Anatolian *D. reticulatus* in a Palearctic Context

Although *D. reticulatus* is recognized as an epidemiologically important tick across the Palearctic region [1, 4, 19], its population genetic structure has remained poorly characterized in the eastern part of its range, including Anatolia. Until now, population‐level data from Türkiye were limited to a small number of specimens from a single northeastern locality included in a continent‐wide microsatellite study [29]. The present study addresses this gap by providing the first regionally structured multi‐marker population genetic dataset for *D. reticulatus* in Türkiye based on mitochondrial (*cox1*), nuclear (ITS2), and concatenated multilocus data.

Across all three datasets, Anatolian *D. reticulatus* populations showed moderate haplotype/genotype diversity, together with low nucleotide diversity. Overall, the observed genetic patterns suggest limited long‐term isolation and relatively weak regional structuring between CN and NE. Similar patterns of shallow population structure and haplotype sharing have previously been reported from Central and Eastern Europe using different genetic markers [30–32, 34].

At the broader Palearctic scale, the observed patterns are generally consistent with previous microsatellite‐based studies showing moderate continental structuring but limited differentiation within major regional groups [29, 33]. Global haplotype and genotype network analyses placed Anatolian variants within widely distributed Eurasian lineages shared with eastern Europe, Russia, and Kazakhstan rather than as a clearly distinct regional cluster. In addition, several low‐frequency haplotypes and genotypes were restricted to either CN or NE populations, indicating the presence of regional sequence variants within a broader Eurasian genetic background.

A notable finding of this study is the contrast with the sympatric *D. marginatus* populations analyzed from the same regions using comparable approaches. Compared with *D. reticulatus*, *D. marginatus* showed stronger regional structuring in Anatolia [27]. These differences suggest that closely related *Dermacentor* species may respond differently to the same geographic and ecological conditions. In this context, the Anatolian Diagonal may have a stronger structuring effect on *D. marginatus* populations than on *D. reticulatus*.

Taken together, the results indicate that Anatolian *D. reticulatus* populations show limited genetic structuring across the studied regions and share broadly similar mitochondrial and nuclear lineages with other eastern Palearctic populations. The observed patterns may reflect historical connectivity and potential host‐mediated dispersal across Anatolia. The contrasting population structure observed in sympatric *D. marginatus* further highlights species‐specific differences in the response of *Dermacentor* ticks to the same biogeographic setting. These findings provide a useful framework for future population genetic and eco‐epidemiological studies of *D. reticulatus* in Anatolia and adjacent regions.

### 4.3. TBP Spectrum in *D. reticulatus* and Comparison With Other *Dermacentor* Species

*Dermacentor reticulatus* is recognized as a vector and carrier of numerous TBPs across the Palearctic region [4, 8–11], although most available data originate from western and central Europe. In Anatolia, information on pathogens associated with *D. reticulatus* has remained very limited despite the region’s ecological heterogeneity and biogeographic importance. By screening host‐seeking ticks collected from multiple localities on both sides of the Anatolian Diagonal, the present study provides the first broad overview of the TBP spectrum associated with *D. reticulatus* in Türkiye. The dataset also allows comparison with sympatric *D. marginatus* populations previously analyzed using the same sampling framework [27].

Screening of 212 host‐seeking *D. reticulatus* revealed that more than one‐quarter of examined ticks carried at least one TBP. Most infections involved a single pathogen, whereas mixed infections were detected only in a small number of ticks and always included *Rickettsia* spp. together with piroplasmids. *Rickettsia* spp. represented the most frequently detected pathogen group, whereas piroplasmids and *C. burnetii* occurred at lower frequencies. In addition, pathogen detections showed marked regional differences, with some agents occurring predominantly in either the CN or NE populations.

#### 4.3.1. *Rickettsia* spp.

*Rickettsia* spp. represented the most frequently detected pathogen group detected in *D. reticulatus* populations in Anatolia, consistent with previous studies identifying this tick as an important carrier of SFG rickettsiae across the Palearctic region [4, 8, 82]. In addition to the high prevalence of rickettsial infections, the most notable finding was their uneven regional distribution across Anatolia.

*Rickettsia* positivity was concentrated mainly in NE, where *R. raoultii* was detected at most positive study sites, whereas only a small number of positive ticks were identified in CN despite the presence of *D. reticulatus* populations in both regions. These findings indicate marked regional differences in the distribution of *R. raoultii* within Anatolian *D. reticulatus* populations.

*Rickettsia slovaca* was detected only sporadically and was restricted to a single northeastern locality, whereas *R. raoultii* clearly predominated in *D. reticulatus*. This pattern is consistent with reports from several European countries in which *R. raoultii* is commonly associated with *D. reticulatus* [12, 83–86]. Comparisons with sympatric *D. marginatus* populations from Anatolia reveal notable species‐specific differences. In *D. marginatus*, both *R. raoultii* and *R. slovaca* have been documented, with regional shifts in predominance between northeastern and CN [28, 87–89]. In contrast, *D. reticulatus* in the present study was associated almost exclusively with *R. raoultii*, particularly in northeastern populations. Together, these observations suggest that sympatric *Dermacentor* species may contribute differently to the circulation of SFG rickettsiae in Anatolia.

From a public health perspective, the frequent detection of *R. raoultii* in host‐seeking *D. reticulatus* is noteworthy because this pathogen is associated with SENLAT syndrome in humans and is primarily linked to *Dermacentor* ticks [82]. Although confirmed human cases have not yet been reported from Türkiye, repeated detections of *R. raoultii* in both *D. marginatus* and *D. reticulatus* indicate that the pathogen is established in several Anatolian tick populations. Increased surveillance and clinical awareness may therefore be warranted, particularly in northeastern regions where *R. raoultii* detections were most common.

Mixed infections involving *Rickettsia* spp. were uncommon and were observed only in combination with piroplasmids. All mixed infections included *R. raoultii* together with either *B. canis* or *B. vulpes*, whereas no co‐infections involving *R. slovaca* were detected. Compared with sympatric *D. marginatus* populations sampled from the same regions [28], *D. reticulatus* showed a lower frequency and diversity of mixed infections. These findings further support differences in pathogen assemblages between the two *Dermacentor* species in Anatolia.

Overall, this study demonstrates that *D. reticulatus* populations in Anatolia are frequently associated with SFG rickettsiae, particularly *R. raoultii*, and that the distribution of these pathogens differs between regions. When considered together with previous findings from sympatric *D. marginatus* populations, the results highlight substantial variation in rickettsial associations among *Dermacentor* species and regions within Anatolia.

#### 4.3.2. *C. burnetii*

*Coxiella burnetii*, the causative agent of Q fever, is a globally distributed zoonotic pathogen associated with both human diseases and reproductive disorders in domestic ruminants [90–92]. Although domestic animals are regarded as the principal reservoirs, ticks are increasingly recognized as potential components of enzootic transmission cycles despite uncertainties regarding their precise epidemiological role under natural conditions [93, 94].

In the present study, *C. burnetii* was detected at a low overall prevalence in *D. reticulatus*; however, 11 of 12 positive ticks originated from a single study site (LGr) in Gerede district (Bolu Province). Positive ticks were collected from four different micro‐localities within this site, indicating a localized spatial distribution of detections. A similar pattern was previously observed in sympatric *D. marginatus* populations sampled from the same region [28], suggesting that *C. burnetii* circulates in multiple *Dermacentor* species in this area.

Previous studies have reported PCR‐based detections of *C. burnetii* in *D. reticulatus* from several European countries [95–98], although the epidemiological significance of these findings remains uncertain. In the present study, all detected sequences belonged to a single IS1111 haplotype that matched previously reported sequences from multiple hosts and geographic regions.

From a One Health perspective, the localized concentration of *C. burnetii*‐positive ticks in the Gerede region is noteworthy, particularly because human Q fever cases and seropositivity have previously been reported from Bolu Province [99, 100]. However, information on *C. burnetii* occurrence in domestic ruminants from the region remains limited. Additional studies integrating tick surveillance, livestock screening, and human seroepidemiology would therefore be useful for clarifying the local epidemiology of *C. burnetii* in this area.

#### 4.3.3. Piroplasmids (*Babesia and Theileria* spp.)

Piroplasmids are an ecologically diverse group of tick‐borne protozoa of major veterinary importance. *Dermacentor reticulatus* has been implicated as a competent and/or suspected vector for several *Babesia* and *Theileria* species in the Palearctic region, although its role appears to vary among pathogens and ecological settings [4, 7, 8, 101]. Until now, information on piroplasmids associated with *D. reticulatus* in Anatolia has been lacking.

*Babesia canis* is widely regarded as the piroplasmid most closely associated with *D. reticulatus* in Europe [7, 101]. Despite this association, previous studies have often reported relatively low prevalences in host‐seeking *D. reticulatus* populations, even in endemic regions [102–106]. The low prevalence detected in the present study is therefore consistent with the patterns observed elsewhere in Europe. Given the pathogenic importance of *B. canis* for domestic dogs [7], continued surveillance of canine babesiosis in Anatolia remains warranted.

The ecology of *B. vulpes* remains incompletely understood, and vector competence has not yet been conclusively demonstrated [107, 108]. Red foxes are considered the principal reservoir hosts [101, 107], and only limited studies have reported *B. vulpes* DNA in host‐seeking ticks, including occasional detections in *D. reticulatus* [109]. In Türkiye, *B. vulpes* has previously been reported from red foxes [110], whereas the present study documents its detection in *D. reticulatus*. A previous study also detected *B. vulpes* in sympatric *D. marginatus* populations from NE [28], indicating that multiple *Dermacentor* species may encounter this parasite within shared ecological systems.

*Babesia occultans* is primarily associated with cattle and is classically linked to *Hyalomma* spp., particularly *H. marginatum* and *H. excavatum* [88, 111–113]. Its detection in *D. reticulatus* in CN is therefore noteworthy because this parasite has not previously been reported from this tick species. A similar finding was reported previously in sympatric *D. marginatus* populations from the same region [28]. Although the epidemiological significance of this finding remains unclear, it expands the currently known host and tick associations of *B. occultans* in Anatolia.

The detection of *T. ovis* in *D. reticulatus* represents, to the best of our knowledge, the first report of this parasite in this tick species. *Theileria ovis* is primarily associated with small ruminants and is generally transmitted by *Rhipicephalus* and *Hyalomma* ticks [101]. A previous study detected *T. ovis* in sympatric *D. marginatus* populations from both CN and NE [28], indicating that *Dermacentor* ticks may occasionally encounter this parasite within shared ecological settings.

Overall, piroplasmids were detected at low prevalence in Anatolian *D. reticulatus* populations but included several taxonomically distinct *Babesia* and *Theileria* species. Together with previous findings from sympatric *D. marginatus* populations, these results indicate that multiple *Dermacentor* species in Anatolia may participate in the circulation of diverse piroplasmids under different ecological conditions.

#### 4.3.4. *A. phagocytophilum*

A single host‐seeking *D. reticulatus* specimen from CN (study site L1) tested positive for *A. phagocytophilum*. Although the low prevalence precludes broader epidemiological interpretation, phylogenetic analysis based on the *groEL* gene placed the detected variant within Ecotype I‐Cluster 1, which includes strains widely distributed across the Palearctic region and commonly associated with *I. ricinus* [114, 115].

Importantly, *I. ricinus* has not been recorded at the L1 study site during long‐term field observations in the region ([80]; ongoing studies). In contrast to many European regions where *D. reticulatus* and *I. ricinus* commonly occur sympatrically [34, 86, 96, 103, 116–119], the Anatolian study sites investigated here were characterized mainly by the co‐occurrence of *D. reticulatus* with *D. marginatus* and several *Haemaphysalis* species.

Previous studies from Europe have also occasionally reported *A. phagocytophilum* DNA in *D. reticulatus* [34, 86, 96, 103, 117, 119]. In Türkiye, data on *A. phagocytophilum* in ticks remain limited [87], and the ecotype‐based analysis applied here provides additional information from a *Dermacentor*‐dominated tick assemblage in Anatolia. Further studies including expanded tick and host sampling will be necessary to clarify the ecological and epidemiological significance of this observation.

### 4.4. Spatial Heterogeneity and Focal Transmission Patterns of TBPs

The present study revealed marked spatial heterogeneity in the distribution of TBPs within Anatolian *D. reticulatus* populations. Similar geographically uneven patterns have been reported in several European studies, where *D. reticulatus*‐associated pathogens clustered within specific landscapes or localities despite broader tick occurrence [12, 34, 86, 96]. In Anatolia, some pathogens were restricted to particular regions or study sites despite sampling across multiple ecologically suitable areas. Comparable spatial heterogeneity has also been reported previously for sympatric *D. marginatus* populations in the same regions [28].

Within Anatolia, rickettsial infections in *D. reticulatus* were detected predominantly in NE, whereas only a few positive ticks were identified in CN. Most infected ticks belonged to shared or NE‐associated *cox1* haplotypes and ITS2 genotypes, whereas CN‐specific variants were only rarely represented among positive specimens. A similar northeastern concentration of rickettsial infections has previously been observed in sympatric *D. marginatus* populations from the same regions [28]. From an evolutionary perspective, such focal persistence is consistent with the biology of SFG rickettsiae, which are shaped by long‐term host–vector associations, genome reduction, and fine‐scale ecological adaptation rather than rapid geographic expansion [120, 121]. Together, these findings indicate that the distribution of *R. raoultii* in Anatolian *Dermacentor* populations is geographically uneven and concentrated mainly in northeastern regions.

The distribution of *C. burnetii* in *D. reticulatus* was even more spatially restricted, with nearly all positive detections originating from a single study site (LGr, Gerede District). Positive ticks were associated mainly with interregional haplotypes and genotypes that also occurred in NE, where *C. burnetii* was not detected. This pattern suggests that local ecological conditions at the LGr site may contribute to the observed concentration of positive ticks. A similar localized pattern was previously reported in sympatric *D. marginatus* populations from the same locality [28].

Taken together, the results indicate that pathogen occurrence in Anatolian *D. reticulatus* populations is spatially heterogeneous rather than uniformly distributed across regions. *Rickettsia raoultii* was detected predominantly in northeastern populations, whereas *C. burnetii* showed a highly localized distribution restricted mainly to a single study site. In contrast, piroplasmids and *A. phagocytophilum* were detected only sporadically and without clear regional patterns. The combined population genetic and pathogen data therefore suggest that pathogen distribution patterns in *D. reticulatus* do not directly mirror the broad genetic structure of tick populations across Anatolia.

### 4.5. Epidemiological Implications and Future Perspectives

The findings of this study have several epidemiological implications for understanding the TBD risk in Anatolia. The observed spatial heterogeneity of pathogen detections indicates that the pathogen occurrence in *D. reticulatus* is unevenly distributed across regions rather than uniformly present throughout the study area. In particular, the concentrated distribution patterns observed for *R. raoultii* and *C. burnetii* highlight the importance of regionally targeted surveillance approaches.

The repeated detection of *R. raoultii* in northeastern *Dermacentor* populations, together with the localized concentration of *C. burnetii* in the Gerede region, suggests that ecological conditions may influence pathogen occurrence at fine spatial scales. These observations further indicate that the TBD risk in Anatolia is likely shaped by local ecological and host‐related factors in addition to tick distribution alone.

From a broader perspective, the contrasting pathogen profiles and population genetic patterns observed in *D. reticulatus* (current study) and sympatric *D. marginatus* populations [27, 28] highlight the importance of species‐specific approaches to TBD ecology. Although these species frequently occur sympatrically and share hosts and habitats, their pathogen associations differ across regions. These findings indicate that surveillance strategies should consider multiple tick species rather than treating *Dermacentor* ticks as a single epidemiological unit.

Several research priorities emerge from this study. Longitudinal monitoring will be important for evaluating the temporal stability of the pathogen patterns identified here and for assessing potential changes under ongoing environmental and land‐use changes. In addition, integrated One Health approaches combining tick surveillance, livestock and wildlife screening, and human seroepidemiological studies would help clarify the ecological and epidemiological significance of the detected pathogen patterns in Anatolia. Future studies integrating tick population structure, vertebrate host data, and environmental variables may further improve understanding of the factors influencing localized pathogen occurrence in tick populations in Türkiye.

### 4.6. Study Limitations

Several limitations of the present study should be considered when interpreting the results. First, the study was based on a cross‐sectional sampling design, which does not allow the assessment of the temporal stability of the detected pathogen patterns or transmission dynamics. Second, pathogen screening was performed exclusively on host‐seeking ticks without concurrent sampling of vertebrate hosts or environmental variables, limiting the interpretation of the ecological drivers underlying localized pathogen occurrence. Third, although the population genetic analyses revealed broad regional patterns, uneven sample sizes among localities prevented robust fine‐scale population subdivision analyses. In addition, detection of *C. burnetii* relied on the multicopy IS1111‐Tnp marker, which provides high analytical sensitivity but may also increase the likelihood of detecting low‐level or environmentally derived DNA; therefore, positive detections should be interpreted cautiously. Finally, several pathogens were detected only sporadically, precluding a detailed statistical assessment of associations between pathogen occurrence and tick genetic background. Despite these limitations, the study provides the first integrated overview of *D. reticulatus* population genetics and associated TBPs in Anatolia and establishes a baseline framework for future eco‐epidemiological investigations in the region.

## 5. Conclusion

This study provides the first integrated assessment of the population genetic structure and associated TBPs of *D. reticulatus* in Anatolia using combined mitochondrial, nuclear, and pathogen screening data. The results indicate that Anatolian *D. reticulatus* populations show limited genetic structuring and share broadly similar lineages with other eastern Palearctic populations. In contrast, pathogen detections displayed marked spatial heterogeneity, with *R. raoultii* occurring predominantly in northeastern populations and *C. burnetii* showing a highly localized distribution. These findings suggest that the broad‐scale tick population structure does not necessarily correspond to pathogen distribution patterns across Anatolia. Comparisons with sympatric *D. marginatus* populations further highlight species‐specific differences in pathogen associations within the same geographic regions. Together, the results emphasize the importance of integrating vector population genetics with spatially explicit pathogen surveillance for improving the understanding of TBD ecology in Anatolia. This study also provides baseline genetic and pathogen data for future eco‐epidemiological investigations and surveillance programs targeting *Dermacentor*‐associated pathogens in Türkiye.

## Author Contributions

**Ömer Orkun:** writing – review & editing, writing – original draft, software, resources, project administration, methodology, investigation, funding acquisition, formal analysis, data curation, conceptualization. **Maide Nur Gündoğdu:** methodology, investigation. **Tuğba Özdemir:** methodology, investigation. **Mesut Yiğit:** methodology, investigation. **Barış Yıldız:** methodology. **Ahmet Deniz:** investigation. **Zati Vatansever:** writing – review & editing, supervision, software, methodology, investigation, formal analysis, data curation, conceptualization.

## Funding

Field work of this study was supported by the Scientific and Technological Research Council of Türkiye (TUBITAK) ARDEB 1001 Programme, Grant No. 121O615. The funding agency had no role in the study design, data collection and analysis, decision to publish, or preparation of the manuscript.

## Ethics Statement

This study did not involve the use of live animals or human participants. All tick specimens were collected as host‐seeking individuals from the environment without direct interaction with vertebrate hosts. Therefore, ethical approval was not required.

## Conflicts of Interest

The authors declare no conflicts of interest.

## Supporting Information

Additional supporting information can be found online in the Supporting Information section.

## Supporting information


**Supporting Information 1** Table S1: Primer sets and PCR cycling conditions used for molecular detection of target organisms in *Dermacentor reticulatus*.

**Supporting Information 2** Table S2: Analysis of molecular variance (AMOVA) and pairwise ΦST estimates based on mitochondrial *cox1* sequences of *Dermacentor reticulatus*. AMOVA results show the distribution of genetic variation within and between Central Anatolia (CN) and Northeastern Anatolia (NE) populations. Pairwise ΦST values were calculated using pairwise nucleotide differences, and statistical significance was assessed by permutation tests.

**Supporting Information 3** Table S3: Genetic diversity indices and neutrality test results based on nuclear ITS2 sequences of *Dermacentor reticulatus* from Anatolia. Summary statistics are shown for Central Anatolia (CN), Northeastern Anatolia (NE), and the pooled dataset (ALL), including number of genotypes, polymorphic sites, haplotype (genotype) diversity, nucleotide diversity, mean number of pairwise differences, and neutrality test statistics (Tajima’s D, Fu and Li’s D and F, and Fu’s Fs).

**Supporting Information 4** Table S4: Analysis of molecular variance (AMOVA) and pairwise ΦST based on nuclear ITS2 sequences of *Dermacentor reticulatus*. AMOVA results show the distribution of genetic variation within and between Central Anatolia (CN) and Northeastern Anatolia (NE) populations. Pairwise ΦST values were calculated using pairwise nucleotide differences, and statistical significance was assessed by permutation tests.

**Supporting Information 5** Table S5: Genetic diversity indices and neutrality test results based on concatenated mitochondrial *cox1* and nuclear ITS2 sequences of *Dermacentor reticulatus*. Summary statistics are presented for Central Anatolia (CN), Northeastern Anatolia (NE), and the pooled dataset (ALL), including number of haplotypes, polymorphic sites, haplotype diversity, nucleotide diversity, mean number of pairwise differences, and neutrality test statistics (Tajima’s D, Fu and Li’s D and F, and Fu’s Fs).

**Supporting Information 6** Table S6: Analysis of molecular variance (AMOVA) and pairwise ΦST based on concatenated mitochondrial *cox1* and nuclear ITS2 sequences of *Dermacentor reticulatus*. AMOVA results show the distribution of genetic variation within and between Central Anatolia (CN) and Northeastern Anatolia (NE) populations. Pairwise ΦST values were calculated using pairwise nucleotide differences, and statistical significance was assessed by permutation tests.

**Supporting Information 7** Table S7: Geographic distribution and accession numbers of mitochondrial *cox1* haplotypes identified in *Dermacentor reticulatus* from Anatolia.

**Supporting Information 8** Table S8: GenBank reference sequences used in Bayesian phylogenetic analysis based on mitochondrial *cox1* haplotypes of *Dermacentor reticulatus*. The table lists accession numbers, country of origin, and corresponding sequence information for *D. reticulatus* reference sequences retrieved from GenBank and included in the *cox1*‐based Bayesian phylogenetic analysis.

**Supporting Information 9** Table S9: List of mitochondrial *cox1* sequences of *Dermacentor reticulatus* used for the global haplotype network analysis. The table includes haplotype codes (HP1–HP32), sample origin (country and region), GenBank accession numbers, sequence length after trimming (606 bp), and inclusion status (this study vs. GenBank‐derived sequences).

**Supporting Information 10** Table S10: Geographic distribution and accession numbers of nuclear ITS2 genotypes identified in *Dermacentor reticulatus* from Anatolia.

**Supporting Information 11** Table S11: GenBank reference sequences used in Bayesian phylogenetic analysis based on nuclear ITS2 genotypes of *Dermacentor reticulatus*. Accession numbers, country of origin, and sequence metadata of *D. reticulatus* reference sequences retrieved from GenBank and included in the ITS2‐based Bayesian phylogenetic analysis are provided.

**Supporting Information 12** Table S12: List of nuclear ITS2 sequences of *Dermacentor reticulatus* used for the global genotype network analysis. The table includes haplotype codes (GN1–GN9), sample origin (country and region), GenBank accession numbers, sequence length after trimming (591 bp), and inclusion status (this study vs. GenBank‐derived sequences).

**Supporting Information 13** Figure S1: Mismatch distribution based on mitochondrial *cox1* sequences of *Dermacentor reticulatus* from Anatolia. Observed (solid line) and expected (dashed line) distributions of pairwise nucleotide differences are shown separately for Central Anatolia (CN), Northeastern Anatolia (NE), and the pooled dataset (ALL). All datasets exhibit unimodal distributions with low raggedness, indicating limited mitochondrial sequence divergence among haplotypes.

**Supporting Information 14** Figure S2: Unsupervised discriminant analysis of principal components (DAPC) based on mitochondrial *cox1* sequences of *Dermacentor reticulatus*. Clustering was performed without a priori population assignment (*k* = 2). The scatter plot shows extensive overlap between inferred clusters, indicating weak genetic structure in the absence of predefined populations.

**Supporting Information 15** Figure S3: Mismatch distribution based on nuclear ITS2 sequences of *Dermacentor reticulatus*. Observed and expected mismatch distributions are shown for the pooled dataset and regional populations, indicating a unimodal pattern consistent with limited nuclear genetic differentiation.

**Supporting Information 16** Figure S4: Nuclear ITS2 genotype structure of *Dermacentor reticulatus* (*n* = 160) from Anatolia. (A) Geographic distribution of ITS2 genotypes across sampling localities. The upper panel shows the overall study area, with enlargements of (A1) Central Anatolia and (A2) Northeastern Anatolia. Pie charts represent genotype composition at each site. Colors denote regional affiliation: Central Anatolia (red shades), Northeastern Anatolia (blue shades), and shared genotypes (yellow–green shades). (B) TCS genotype network inferred from ITS2 data. Circle size is proportional to genotype frequency, and each connection represents a single mutational step unless otherwise indicated. Colors correspond to geographic origin (Central Anatolia = red; Northeastern Anatolia = blue). (C) Color‐coded matrix of pairwise nucleotide identity among ITS2 haplotypes.

**Supporting Information 17** Figure S5: Supervised discriminant analysis of principal components (DAPC) based on nuclear ITS2 sequences of *Dermacentor reticulatus*. Individuals were assigned a priori to Central Anatolia (CN) and Northeastern Anatolia (NE) populations. The analysis shows extensive overlap between regions, indicating weak nuclear population structure.

**Supporting Information 18** Figure S6: Unsupervised discriminant analysis of principal components (DAPC) based on nuclear ITS2 sequences of *Dermacentor reticulatus*. Clustering was performed without a priori population assignment (*k* = 2). The scatter plot shows extensive overlap between inferred clusters, indicating weak nuclear genetic structure in the absence of predefined populations.

**Supporting Information 19** Figure S7: Mismatch distribution based on concatenated mitochondrial *cox1* and nuclear ITS2 sequences of *Dermacentor reticulatus*. Observed and expected mismatch distributions are shown for the pooled dataset and regional populations, indicating unimodal patterns with low raggedness.

**Supporting Information 20** Figure S8: Multilocus genotype structure of *Dermacentor reticulatus* (*n* = 160) from Anatolia based on concatenated mitochondrial *cox1* and nuclear ITS2 sequences. (A) Geographic distribution of multilocus genotypes across sampling localities. The upper panel shows the overall study area, with enlargements of (A1) Central Anatolia and (A2) Northeastern Anatolia. Pie charts represent genotype composition at each site. Colors denote regional affiliation: Central Anatolia (red shades), Northeastern Anatolia (blue shades), and shared genotypes (yellow–green shades). (B) TCS genotype network inferred from concatenated dataset. Circle size is proportional to genotype frequency, and each connection represents a single mutational step unless otherwise indicated. Colors correspond to geographic origin (Central Anatolia = red; Northeastern Anatolia = blue). (C) Color‐coded matrix of pairwise nucleotide identity among multilocus haplotypes.

**Supporting Information 21** Figure S9: Supervised discriminant analysis of principal components (DAPC) based on concatenated mitochondrial *cox1* and nuclear ITS2 sequences of *Dermacentor reticulatus*. Individuals were assigned a priori to Central Anatolia (CN) and Northeastern Anatolia (NE) populations. The analysis shows partial overlap between regions, indicating weak to moderate multilocus population structure.

**Supporting Information 22** Figure S10: Unsupervised discriminant analysis of principal components (DAPC) based on concatenated mitochondrial *cox1* and nuclear ITS2 sequences of *Dermacentor reticulatus*. Clustering was performed without a priori population assignment (*k* = 2). The scatter plot shows extensive overlap between inferred clusters, supporting weak multilocus genetic structure.

**Supporting Information 23** Figure S11: Bayesian phylogenetic placement of mitochondrial *cox1* haplotypes and nuclear ITS2 genotypes of *Dermacentor reticulatus*. Bayesian phylogenetic trees were inferred using BEAST2 based on (a) mitochondrial *cox1* haplotypes and (b) nuclear ITS2 genotypes identified in this study together with reference sequences retrieved from GenBank. Branches with posterior probabilities below 0.8 were collapsed. Posterior probability values are shown at major nodes. *Dermacentor marginatus* and *Dermacentor raskemensis* sequences were used as outgroups.

**Supporting Information 24** Figure S12: Global haplotype network of *Dermacentor reticulatus* based on mitochondrial *cox1* sequences. The network was constructed using the TCS algorithm and includes 260 sequences (160 from this study and 100 GenBank‐derived sequences with known geographic origin), trimmed to a common length of 606 bp. A total of 32 haplotypes (HP1–HP32) were identified. Circle sizes are proportional to haplotype frequencies and each connecting line represents a single mutational step. Haplotypes detected in this study are highlighted, and their distribution across Central Anatolia (CN) and Northeastern Anatolia (NE) is indicated. The list of countries corresponding to each haplotype is provided alongside the network.

**Supporting Information 25** Figure S13: Global genotype network of *Dermacentor reticulatus* based on nuclear ITS2 sequences. The network was constructed using the TCS algorithm and includes 177 sequences (160 from this study and 17 GenBank‐derived sequences with known geographic origin), trimmed to a common length of 591 bp. A total of 9 genotypes (GN1–GN9) were identified. Circle sizes are proportional to genotype frequencies and each connecting line represents a single mutational step. Genotypes detected in this study are highlighted, and their distribution across Central Anatolia (CN) and Northeastern Anatolia (NE) is indicated. The list of countries corresponding to each genotype is provided alongside the network.

**Supporting Information 26** Figure S14: Phylogenetic tree constructed using Bayesian inference based on aligned nucleotide sequences of the 18S rRNA gene of piroplasms, with *Cardiosporidium cionae* (EU052685) as the outgroup, under the TN93+I+G substitution model. The analysis included 53 sequences and 1,670 positions. Node labels indicate posterior probabilities, with values below 0.75 omitted. Haplotype sequences obtained in this study are highlighted in colors. GenBank® accession numbers are provided before species names. The scale bar represents nucleotide substitutions per site.

**Supporting Information 27** Figure S15: Phylogenetic tree constructed using Bayesian inference based on aligned nucleotide sequences of the *groEL* gene of *Anaplasma phagocytophilum* under the HKY+G substitution model. The analysis included 61 sequences and 530 positions. Node labels indicate posterior probabilities, with values below 0.7 omitted. The haplotype sequence obtained in this study and its corresponding clade (Ecotype 1/Cluster 1) are highlighted in red. GenBank® accession numbers are provided before species names. The scale bar represents nucleotide substitutions per site.

## Data Availability

The sequence data that support the findings of this study are openly available in GenBank (https://www.ncbi.nlm.nih.gov/genbank/) and BOLD (http://www.boldsystems.org) databases under accession numbers provided in Table 3, Supporting Information 7: Table S7, and Supporting Information 10: Table S10. All the data involved in this study are available from the corresponding author upon request.
