# Supplementary material for: Spatial Heterogeneity of Tick‐Borne Pathogens Outpaces Genetic Structuring in Anatolian Dermacentor reticulatus Populations
Source: Transbound Emerg Dis. 2026 Jul 22;2026:5552728. doi: 10.1155/tbed/5552728 (PMC13390018; doi:10.1155/tbed/5552728)
Supplement: Supplementary file 3 — Supporting Information 3 Table S3: Genetic diversity indices and neutrality test results based on nuclear ITS2 sequences of Dermacentor reticulatus from Anatolia. Summary statistics are shown for Central Anatolia (CN), Northeastern Anatolia (NE), and the pooled dataset (ALL), including number of genotypes, polymorphic sites, haplotype (genotype) diversity, nucleotide diversity, mean number of pairwise differences, and neutrality test statistics (Tajima’s D, Fu and Li’s D and F, and Fu’s Fs). [file TBED-2026-5552728-s027.docx]

**Supplementary Table 3.** **Genetic diversity indices and neutrality test results based on nuclear ITS2 sequences of Dermacentor reticulatus from Anatolia.** Summary statistics are shown for Central Anatolia (CN), Northeastern Anatolia (NE), and the pooled dataset (ALL), including number of genotypes, polymorphic sites, haplotype (genotype) diversity, nucleotide diversity, mean number of pairwise differences, and neutrality test statistics (Tajima’s D, Fu and Li’s D and F, and Fu’s Fs).

| Population | *n* | *h* | *Hd* | *π* | *S* | *k* | Fu’s Fs | Tajima’s D | Fu and Li’s D | Fu and Li’s F |
| --- | --- | --- | --- | --- | --- | --- | --- | --- | --- | --- |
| CN | 98 | 20 | 0.858 | 0.00203 | 11 | 2.202 | -9,299 | 0,08436 | -0,55788 | -0,3941 |
| NE | 62 | 18 | 0.852 | 0.00201 | 9 | 2.182 | -9,209 | 0,37316 | -0,72562 | -0,42485 |
| ALL | 160 | 27 | 0.858 | 0.00204 | 15 | 2,214 | -15,815 | -0,43392 | -0,78358 | -0,78071 |

*n* = number of individuals, *h* = number of haplotypes, *Hd* = haplotype diversity, *π* = nucleotide diversity, *S* = number of segregation sites, and *k* = average number of nucleotide differences, * Statistically significant (P<0.05)
