# Supplementary material for: Spatial Heterogeneity of Tick‐Borne Pathogens Outpaces Genetic Structuring in Anatolian Dermacentor reticulatus Populations
Source: Transbound Emerg Dis. 2026 Jul 22;2026:5552728. doi: 10.1155/tbed/5552728 (PMC13390018; doi:10.1155/tbed/5552728)
Supplement: Supplementary file 4 — Supporting Information 4 Table S4: Analysis of molecular variance (AMOVA) and pairwise ΦST based on nuclear ITS2 sequences of Dermacentor reticulatus. AMOVA results show the distribution of genetic variation within and between Central Anatolia (CN) and Northeastern Anatolia (NE) populations. Pairwise ΦST values were calculated using pairwise nucleotide differences, and statistical significance was assessed by permutation tests. [file TBED-2026-5552728-s023.docx]

**Supplementary Table 4.** **Analysis of molecular variance (AMOVA) and pairwise ΦST based on nuclear ITS2 sequences of Dermacentor reticulatus.** AMOVA results show the distribution of genetic variation within and between Central Anatolia (CN) and Northeastern Anatolia (NE) populations. Pairwise ΦST values were calculated using pairwise nucleotide differences, and statistical significance was assessed by permutation tests.

1. **AMOVA results:**

| Source of variation | d.f. | Sum of squares | Variance components | Percentage variation |
| --- | --- | --- | --- | --- |
| Among populations (CN vs NE) | 1 | 5.344 | 0.02801 | 2.50 |
| Within populations | 318 | 346.668 | 1.09015 | 97.50 |
| Total | **319** | **352.012** | **1.11816** | **-** |

**Fixation index (ΦST) = 0.02505**

Permutation test (1023 permutations): P =0.071

1. **Pairwise population differentiation (ΦST)**

| Population | CN | NE |
| --- | --- | --- |
| CN | - | 0.02505 |
| NE | 0.02505 | - |

Permutation test (110 permutations): CN vs NE, P =0.054
