# Supplementary material for: Spatial Heterogeneity of Tick‐Borne Pathogens Outpaces Genetic Structuring in Anatolian Dermacentor reticulatus Populations
Source: Transbound Emerg Dis. 2026 Jul 22;2026:5552728. doi: 10.1155/tbed/5552728 (PMC13390018; doi:10.1155/tbed/5552728)
Supplement: Supplementary file 7 — Supporting Information 7 Table S7: Geographic distribution and accession numbers of mitochondrial cox1 haplotypes identified in Dermacentor reticulatus from Anatolia. [file TBED-2026-5552728-s020.docx]

**Supplementary Table 7.** Geographic distribution and accession numbers of mitochondrial *cox1* haplotypes identified in *Dermacentor reticulatus* from Anatolia.

| **Haplotype name** | **Study region** | **n** | **L1** | **L2** | **L3** | **L4** | **L7** | **LGr** | **L16** | **L17** | **L18** | **L20** | **L21** | **L22** | **L26** | **GenBank Accession no.** | **BOLD ID** |
| --- | --- | --- | --- | --- | --- | --- | --- | --- | --- | --- | --- | --- | --- | --- | --- | --- | --- |
| Cox-CN1 | CN | 1 | 1 |  |  |  |  |  |  |  |  |  |  |  |  | PX789633 | DRE001-26 |
| Cox-CN2 | CN | 6 | 6 |  |  |  |  |  |  |  |  |  |  |  |  | PX789636 | DRE002-26 |
| Cox-CN3 | CN | 2 | 1 |  | 1 |  |  |  |  |  |  |  |  |  |  | PX789637 | DRE003-26 |
| Cox-CN4 | CN | 1 | 1 |  |  |  |  |  |  |  |  |  |  |  |  | PX789638 | DRE004-26 |
| Cox-CN5 | CN | 1 | 1 |  |  |  |  |  |  |  |  |  |  |  |  | PX789639 | DRE005-26 |
| Cox-CN6 | CN | 1 | 1 |  |  |  |  |  |  |  |  |  |  |  |  | PX789640 | DRE006-26 |
| Cox-CN7 | CN | 1 |  | 1 |  |  |  |  |  |  |  |  |  |  |  | PX789641 | DRE007-26 |
| Cox-CN8 | CN | 3 |  |  | 1 |  | 2 |  |  |  |  |  |  |  |  | PX789642 | DRE008-26 |
| Cox-CN9 | CN | 3 |  |  |  |  |  | 3 |  |  |  |  |  |  |  | PX789643 | DRE009-26 |
| Cox-NE1 | NE | 23 |  |  |  |  |  |  | 1 |  | 3 |  | 8 | 7 | 4 | PX789644 | DRE010-26 |
| Cox-NE2 | NE | 1 |  |  |  |  |  |  |  |  | 1 |  |  |  |  | PX789645 | DRE011-26 |
| Cox-NE3 | NE | 1 |  |  |  |  |  |  |  |  | 1 |  |  |  |  | PX789646 | DRE012-26 |
| Cox-NE4 | NE | 3 |  |  |  |  |  |  |  |  |  |  | 3 |  |  | PX789647 | DRE013-26 |
| Cox-NE5 | NE | 3 |  |  |  |  |  |  |  |  |  |  | 1 | 2 |  | PX789648 | DRE014-26 |
| Cox-CNNE1 | CN+NE | 107 | 41 | 13 | 11 | 1 | 4 | 7 | 8 | 3 | 2 | 3 | 3 | 6 | 5 | PX789634 | DRE015-26 |
| Cox-CNNE2 | CN+NE | 3 | 2 |  |  |  |  |  |  |  |  |  |  | 1 |  | PX789635 | DRE016-26 |

L1–L26 correspond to study sites as listed in Table 1.

CN: Central Anatolia

NE: Northeast Anatolia
