# Supplementary material for: Spatial Heterogeneity of Tick‐Borne Pathogens Outpaces Genetic Structuring in Anatolian Dermacentor reticulatus Populations
Source: Transbound Emerg Dis. 2026 Jul 22;2026:5552728. doi: 10.1155/tbed/5552728 (PMC13390018; doi:10.1155/tbed/5552728)
Supplement: Supplementary file 8 — Supporting Information 8 Table S8: GenBank reference sequences used in Bayesian phylogenetic analysis based on mitochondrial cox1 haplotypes of Dermacentor reticulatus. The table lists accession numbers, country of origin, and corresponding sequence information for D. reticulatus reference sequences retrieved from GenBank and included in the cox1‐based Bayesian phylogenetic analysis. [file TBED-2026-5552728-s019.docx]

**Supplementary Table 8.** **GenBank reference sequences used in Bayesian phylogenetic analysis based on mitochondrial cox1 haplotypes of Dermacentor reticulatus.** The table lists accession numbers, country of origin, and corresponding sequence information for D. reticulatus reference sequences retrieved from GenBank and included in the cox1-based Bayesian phylogenetic analysis.

| **Haplogroup** | **GenBank accession number** | **Organism name** | **Country** |
| --- | --- | --- | --- |
| **Clade1** | AF132829 | Dermacentor_reticulatus | Slovakia |
| **Clade1** | PP048520 | Dermacentor_reticulatus_voucher_PHE022017E11 | UK |
| **Clade1** | OM142140 | Dermacentor_reticulatus_voucher_9022_haplotype_3 | Czech Republic |
| **Clade1** | OR162321 | Dermacentor_reticulatus_strain_Derm_ret1_Bashkortostan | Republic of Bashkortostan (Russia) |
| **Clade1** | OR162322 | Dermacentor_reticulatus_strain_Derm_ret2_Bashkortostan | Republic of Bashkortostan (Russia) |
| **Clade1** | OR162333 | Dermacentor_reticulatus_strain_Derm_ret1_Kaliningrad | Russia |
| **Clade1** | PP048578 | Dermacentor_reticulatus_voucher_CCDB_04692_G03 | Germany |
| **Clade1** | OM142144 | Dermacentor_reticulatus_voucher_33_haplotype_7 | Belarus |
| **Clade1** | PP048239 | Dermacentor_reticulatus_voucher_APHA_14_2016B05 | UK |
| **Clade1** | PP047945 | Dermacentor_reticulatus_voucher_APHA_14_2016F12 | UK |
| **Clade1** | PP048087 | Dermacentor_reticulatus_voucher_PHE022017G09 | UK |
| **Clade1** | PP048253 | Dermacentor_reticulatus_voucher_APHA_14_2016A05 | UK |
| **Clade1** | PX513587 | Dermacentor_reticulatus | UK |
| **Clade1** | OM867318 | Dermacentor_reticulatus_strain_Derm_ret13_Stavropol | Russia |
| **Clade1** | MZ305512 | Dermacentor_reticulatus_voucher_CROBB795 | Croatia |
| **Clade1** | MN308063 | Dermacentor_reticulatus | France |
| **Clade1** | OL639113 | Dermacentor_reticulatus_voucher_T9_8 | Germany |
| **Clade1** | OQ947121 | Dermacentor_reticulatus_isolate_N439 | Poland |
| **Clade1** | PP047811 | Dermacentor_reticulatus_voucher_CCDB_04692_G12 | Czech Republic |
| **Clade1** | PP048044 | Dermacentor_reticulatus_voucher_CCDB_04692_H01 | Czech Republic |
| **Clade1** | PP047785 | Dermacentor_reticulatus_voucher_PHE022017D07 | UK |
| **Clade1** | PP048180 | Dermacentor_reticulatus_voucher_PHE022017B07 | UK |
| **Clade1** | OM867309 | Dermacentor_reticulatus_strain_Derm_ret2_Stavropol | Russia |
| **Clade1** | OM867328 | Dermacentor_reticulatus_strain_Derm_ret21_Novosibirsk | Russia |
| **Clade1** | MT506455 | Dermacentor_reticulatus_isolate_H_4C_2 | Kazakhstan |
| **Clade1** | OR936107 | Dermacentor_reticulatus_strain_ret1_Mordovia | Republic of Mordovia (Russia) |
| **Clade1** | PP048090 | Dermacentor_reticulatus_voucher_APHA_14_2016G04 | UK |
| **Clade1** | OM142145 | Dermacentor_reticulatus_voucher_11623_haplotype_8 | Russia |
| **Clade1** | PQ682399 | Dermacentor_reticulatus_isolate_Nov21_164_Dret | Russia |
| **Clade1** | PQ682400 | Dermacentor_reticulatus_isolate_Nov21_171_Dret | Russia |
| **Clade1** | OM142146 | Dermacentor_reticulatus_voucher_11633_haplotype_9 | Russia |
| **Clade1** | PX672340 | Dermacentor_reticulatus_voucher_TO24002 | Russia |
| **Clade1** | PX672353 | Dermacentor_reticulatus_voucher_KZ25876_2 | Kazakhstan |
| **Clade1** | OR533790 | Dermacentor_reticulatus_isolate_South_Kazakhstan1_89_5 | Kazakhstan |
| **Clade1** | PX672348 | Dermacentor_reticulatus_voucher_GR24676_3B | Russia |
| **Clade2** | MZ305511 | Dermacentor_reticulatus_voucher_CROBB792 | Croatia |
| **Clade2** | MZ305510 | Dermacentor_reticulatus_voucher_CROBB798 | Croatia |
| **Clade2** | MZ305515 | Dermacentor_reticulatus_voucher_CROBB796 | Croatia |
| **Clade2** | MZ305513 | Dermacentor_reticulatus_voucher_CROBB800 | Croatia |
| **outgroup** | MT308586 | Dermacentor_raskemensis_isolate_B_1109 | Türkiye |
| **outgroup** | PP455525 | Dermacentor_marginatus_isolate_CX_CN13 | Türkiye |
