# Supplementary material for: Spatial Heterogeneity of Tick‐Borne Pathogens Outpaces Genetic Structuring in Anatolian Dermacentor reticulatus Populations
Source: Transbound Emerg Dis. 2026 Jul 22;2026:5552728. doi: 10.1155/tbed/5552728 (PMC13390018; doi:10.1155/tbed/5552728)
Supplement: Supplementary file 9 — Supporting Information 9 Table S9: List of mitochondrial cox1 sequences of Dermacentor reticulatus used for the global haplotype network analysis. The table includes haplotype codes (HP1–HP32), sample origin (country and region), GenBank accession numbers, sequence length after trimming (606 bp), and inclusion status (this study vs. GenBank‐derived sequences). [file TBED-2026-5552728-s018.docx]

**Supplementary Table 9. List of mitochondrial *cox1* sequences of *Dermacentor reticulatus* used for the global haplotype network analysis.** The table includes haplotype codes (HP1–HP32), sample origin (country and region), GenBank accession numbers, sequence length after trimming (606 bp), and inclusion status (this study vs. GenBank-derived sequences).

| **Haplotype label** | **Sequence name** | **Country** |
| --- | --- | --- |
| HP6 | OM867315_Dermacentor_reticulatus_strain_Derm_ret8 | Russia |
|  | OM867311_Dermacentor_reticulatus_strain_Derm_ret4 | Russia |
|  | OM867319_Dermacentor_reticulatus_strain_Derm_ret15 | Russia |
| HP7 | Cr_2111 | Türkiye (NE) |
|  | Cr_2112 | Türkiye (NE) |
|  | Cr_2171 | Türkiye (NE) |
| HP8 | OR936107_Dermacentor_reticulatus_strain_ret1 | Russia |
|  | OR936108_Dermacentor_reticulatus_strain_ret1 | Russia |
| HP9 | Cr_2131 | Türkiye (NE) |
|  | Cr_2216 | Türkiye (NE) |
|  | Cr_22112 | Türkiye (NE) |
| HP1 | Cr_342 | Türkiye (CN) |
|  | Cr_7101 | Türkiye (CN) |
|  | Cr_752 | Türkiye (CN) |
|  | Cr_197 | Türkiye (CN) |
|  | Cr_191 | Türkiye (CN) |
|  | Cr_182 | Türkiye (CN) |
|  | Cr_181 | Türkiye (CN) |
|  | Cr_174 | Türkiye (CN) |
|  | Cr_172 | Türkiye (CN) |
|  | Cr_161 | Türkiye (CN) |
|  | Cr_153 | Türkiye (CN) |
|  | Cr_143 | Türkiye (CN) |
|  | Cr_142 | Türkiye (CN) |
|  | Cr_139 | Türkiye (CN) |
|  | Cr_136 | Türkiye (CN) |
|  | Cr_135 | Türkiye (CN) |
|  | Cr_126 | Türkiye (CN) |
|  | Cr_124 | Türkiye (CN) |
|  | Cr_123 | Türkiye (CN) |
|  | Cr_115 | Türkiye (CN) |
|  | Cr_111 | Türkiye (CN) |
|  | Cr_1319 | Türkiye (CN) |
|  | Cr_1316 | Türkiye (CN) |
|  | Cr_1313 | Türkiye (CN) |
|  | Cr_1312 | Türkiye (CN) |
|  | Cr_1311 | Türkiye (CN) |
|  | Cr_1315 | Türkiye (CN) |
|  | Cr_1318 | Türkiye (CN) |
|  | Cr_114 | Türkiye (CN) |
|  | Cr_116 | Türkiye (CN) |
|  | Cr_121 | Türkiye (CN) |
|  | Cr_122 | Türkiye (CN) |
|  | Cr_125 | Türkiye (CN) |
|  | Cr_128 | Türkiye (CN) |
|  | Cr_129 | Türkiye (CN) |
|  | Cr_133 | Türkiye (CN) |
|  | Cr_151 | Türkiye (CN) |
|  | Cr_131 | Türkiye (CN) |
|  | Cr_132 | Türkiye (CN) |
|  | Cr_137 | Türkiye (CN) |
|  | Cr_141 | Türkiye (CN) |
|  | Cr_163 | Türkiye (CN) |
|  | Cr_1310 | Türkiye (CN) |
|  | Cr_1111 | Türkiye (CN) |
|  | Cr_2716 | Türkiye (CN) |
|  | Cr_2712 | Türkiye (CN) |
|  | Cr_271 | Türkiye (CN) |
|  | Cr_265 | Türkiye (CN) |
|  | Cr_264 | Türkiye (CN) |
|  | Cr_263 | Türkiye (CN) |
|  | Cr_262 | Türkiye (CN) |
|  | Cr_261 | Türkiye (CN) |
|  | Cr_255 | Türkiye (CN) |
|  | Cr_253 | Türkiye (CN) |
|  | Cr_211 | Türkiye (CN) |
|  | Cr_2711 | Türkiye (CN) |
|  | Cr_241 | Türkiye (CN) |
|  | Cr_3102 | Türkiye (CN) |
|  | Cr_391 | Türkiye (CN) |
|  | Cr_325 | Türkiye (CN) |
|  | Cr_324 | Türkiye (CN) |
|  | Cr_323 | Türkiye (CN) |
|  | Cr_321 | Türkiye (CN) |
|  | Cr_322 | Türkiye (CN) |
|  | Cr_331 | Türkiye (CN) |
|  | Cr_3101 | Türkiye (CN) |
|  | Cr_3103 | Türkiye (CN) |
|  | Cr_3104 | Türkiye (CN) |
|  | Cr_411 | Türkiye (CN) |
|  | Cr_7121 | Türkiye (CN) |
|  | Cr_751 | Türkiye (CN) |
|  | Cr_721 | Türkiye (CN) |
|  | Cr_761 | Türkiye (CN) |
|  | Cr_Gr_Y11 | Türkiye (CN) |
|  | Cr_Gr_T110 | Türkiye (CN) |
|  | Cr_Gr_T19 | Türkiye (CN) |
|  | Cr_Gr_T16 | Türkiye (CN) |
|  | Cr_Gr_M11 | Türkiye (CN) |
|  | Cr_Gr_T18 | Türkiye (CN) |
|  | Cr_Gr_Y12 | Türkiye (CN) |
|  | Cr_1621 | Türkiye (NE) |
|  | Cr_1616 | Türkiye (NE) |
|  | Cr_1615 | Türkiye (NE) |
|  | Cr_1614 | Türkiye (NE) |
|  | Cr_1611 | Türkiye (NE) |
|  | Cr_1612 | Türkiye (NE) |
|  | Cr_1613 | Türkiye (NE) |
|  | Cr_1631 | Türkiye (NE) |
|  | Cr_1712 | Türkiye (NE) |
|  | Cr_1711 | Türkiye (NE) |
|  | Cr_1721 | Türkiye (NE) |
|  | Cr_1832 | Türkiye (NE) |
|  | Cr_1821 | Türkiye (NE) |
|  | Cr_2042 | Türkiye (NE) |
|  | Cr_2041 | Türkiye (NE) |
|  | Cr_2043 | Türkiye (NE) |
|  | Cr_21232 | Türkiye (NE) |
|  | Cr_2164 | Türkiye (NE) |
|  | Cr_2132 | Türkiye (NE) |
|  | Cr_2218 | Türkiye (NE) |
|  | Cr_22110 | Türkiye (NE) |
|  | Cr_2222 | Türkiye (NE) |
|  | Cr_2211 | Türkiye (NE) |
|  | Cr_2213 | Türkiye (NE) |
|  | Cr_22114 | Türkiye (NE) |
|  | Cr_2655 | Türkiye (NE) |
|  | Cr_2654 | Türkiye (NE) |
|  | Cr_2653 | Türkiye (NE) |
|  | Cr_2621 | Türkiye (NE) |
|  | Cr_2652 | Türkiye (NE) |
|  | OM867309_Dermacentor_reticulatus_strain_Derm_ret2 | Russia |
|  | OM867325_Dermacentor_reticulatus_strain_Derm_ret18 | Russia |
|  | OM867322_Dermacentor_reticulatus_strain_Derm_ret12 | Russia |
|  | OM867312_Dermacentor_reticulatus_strain_Derm_ret5 | Russia |
|  | OM867323_Dermacentor_reticulatus_strain_Derm_ret14 | Russia |
|  | OM867313_Dermacentor_reticulatus_strain_Derm_ret6 | Russia |
|  | OM867314_Dermacentor_reticulatus_strain_Derm_ret7 | Russia |
|  | OM867310_Dermacentor_reticulatus_strain_Derm_ret3 | Russia |
|  | OM867324_Dermacentor_reticulatus_strain_Derm_ret17 | Russia |
|  | OM867316_Dermacentor_reticulatus_strain_Derm_ret9 | Russia |
|  | OM867317_Dermacentor_reticulatus_strain_Derm_ret10 | Russia |
|  | OM867320_Dermacentor_reticulatus_strain_Derm_ret16 | Russia |
|  | NC_068757_Dermacentor_reticulatus_strain_Derm_ret1 | Russia |
|  | OR936113_Dermacentor_reticulatus_strain_ret5 | Russia |
|  | OM867321_Dermacentor_reticulatus_strain_Derm_ret11 | Russia |
|  | OR162331_Dermacentor_reticulatus_strain_Derm_ret1 | Russia |
|  | OR936110_Dermacentor_reticulatus_strain_ret2 | Russia |
|  | OR936112_Dermacentor_reticulatus_strain_ret4 | Russia |
|  | OM867326_Dermacentor_reticulatus_strain_Derm_ret19 | Russia |
|  | OR936111_Dermacentor_reticulatus_strain_ret3 | Russia |
|  | OR936109_Dermacentor_reticulatus_strain_ret1 | Russia |
|  | Cr_127 | Türkiye (CN) |
|  | Cr_138 | Türkiye (CN) |
|  | Cr_2217 | Türkiye (NE) |
|  | OM867327_Dermacentor_reticulatus_strain_Derm_ret20 | Russia |
|  | OM867328_Dermacentor_reticulatus_strain_Derm_ret21 | Russia |
| HP10 | Cr_1314 | Türkiye (CN) |
|  | Cr_341 | Türkiye (CN) |
| HP4 | Cr_1661 | Türkiye (NE) |
|  | Cr_1841 | Türkiye (NE) |
|  | Cr_1851 | Türkiye (NE) |
|  | Cr_1853 | Türkiye (NE) |
|  | Cr_21231 | Türkiye (NE) |
|  | Cr_21212 | Türkiye (NE) |
|  | Cr_2177 | Türkiye (NE) |
|  | Cr_2173 | Türkiye (NE) |
|  | Cr_2121 | Türkiye (NE) |
|  | Cr_2162 | Türkiye (NE) |
|  | Cr_2175 | Türkiye (NE) |
|  | Cr_21211 | Türkiye (NE) |
|  | Cr_2212 | Türkiye (NE) |
|  | Cr_2223 | Türkiye (NE) |
|  | Cr_2219 | Türkiye (NE) |
|  | Cr_2214 | Türkiye (NE) |
|  | Cr_2215 | Türkiye (NE) |
|  | Cr_2221 | Türkiye (NE) |
|  | Cr_22111 | Türkiye (NE) |
|  | Cr_2611 | Türkiye (NE) |
|  | Cr_2651 | Türkiye (NE) |
|  | Cr_2656 | Türkiye (NE) |
|  | Cr_2657 | Türkiye (NE) |
| HP3 | PP047945_Dermacentor_reticulatus_voucher_APHA_14_2016F12 | UK |
|  | PP048100_Dermacentor_reticulatus_voucher_APHA_14_2016B08 | UK |
|  | PP048253_Dermacentor_reticulatus_voucher_APHA_14_2016A05 | UK |
|  | MZ305512_Dermacentor_reticulatus_voucher_CROBB795 | Croatia |
|  | AF132829_Dermacentor_reticulatus | Slovaca |
|  | OR162333_Dermacentor_reticulatus_strain_Derm_ret1 | Russia |
|  | OR162337_Dermacentor_reticulatus_strain_Derm_ret5 | Russia |
|  | OR162336_Dermacentor_reticulatus_strain_Derm_ret4 | Russia |
|  | PX513587_Dermacentor_reticulatus | UK |
|  | OM142138_Dermacentor_reticulatus_voucher_9052_haplotype_1 | Czech Republic |
|  | OQ947121_Dermacentor_reticulatus_isolate_N439 | Poland |
|  | OL639109_Dermacentor_reticulatus_voucher_P10_3 | Germany |
|  | OL639107_Dermacentor_reticulatus_voucher_P10_1 | Germany |
|  | OL639115_Dermacentor_reticulatus_voucher_T9_10 | Germany |
|  | OL639106_Dermacentor_reticulatus_voucher_H5_163 | Germany |
|  | OL639111_Dermacentor_reticulatus_voucher_P11_39 | Germany |
|  | OL639113_Dermacentor_reticulatus_voucher_T9_8 | Germany |
|  | OL639105_Dermacentor_reticulatus_voucher_H5_162 | Germany |
|  | OL639110_Dermacentor_reticulatus_voucher_P10_143 | Germany |
|  | OL639104_Dermacentor_reticulatus_voucher_H5_161 | Germany |
|  | OL639108_Dermacentor_reticulatus_voucher_P10_2 | Germany |
|  | PP048387_Dermacentor_reticulatus_voucher_APHA_14_2016A06 | UK |
|  | OR162335_Dermacentor_reticulatus_strain_Derm_ret3 | Russia |
|  | OR162334_Dermacentor_reticulatus_strain_Derm_ret2 | Russia |
|  | OR162332_Dermacentor_reticulatus_strain_Derm_ret2 | Russia |
| HP11 | OM142139_Dermacentor_reticulatus_voucher_9037_haplotype_2 | Czech Republic |
|  | PP048578_Dermacentor_reticulatus_voucher_CCDB_04692_G03 | Germany |
| HP12 | OM142141_Dermacentor_reticulatus_voucher_9041_haplotype_4 | Czech Republic |
|  | PP047811_Dermacentor_reticulatus_voucher_CCDB_04692_G12 | Czech Republic |
|  | PP048044_Dermacentor_reticulatus_voucher_CCDB_04692_H01 | Czech Republic |
| HP13 | Cr_Gr_M12 | Türkiye (CN) |
|  | Cr_Gr_H11 | Türkiye (CN) |
|  | Cr_Gr_H12 | Türkiye (CN) |
| HP5 | Cr_1113 | Türkiye (CN) |
|  | Cr_113 | Türkiye (CN) |
|  | Cr_117 | Türkiye (CN) |
|  | Cr_118 | Türkiye (CN) |
|  | Cr_119 | Türkiye (CN) |
|  | Cr_1110 | Türkiye (CN) |
|  | Cr_1112 | Türkiye (CN) |
| HP2 | MT478096_Dermacentor_reticulatus | Russia |
|  | OR936099_Dermacentor_reticulatus_strain_ret3 | Russia |
|  | OR936098_Dermacentor_reticulatus_strain_ret2 | Russia |
|  | OM867329_Dermacentor_reticulatus_strain_Derm_ret22 | Russia |
|  | OM867333_Dermacentor_reticulatus_strain_Derm_ret26 | Russia |
|  | OR936097_Dermacentor_reticulatus_strain_ret1 | Russia |
|  | OR936101_Dermacentor_reticulatus_strain_ret5 | Russia |
|  | OM867331_Dermacentor_reticulatus_strain_Derm_ret24 | Russia |
|  | OM867335_Dermacentor_reticulatus_strain_Derm_ret28 | Russia |
|  | OR162327_Dermacentor_reticulatus_strain_Derm_ret2 | Russia |
|  | OM867334_Dermacentor_reticulatus_strain_Derm_ret27 | Russia |
|  | OR162329_Dermacentor_reticulatus_strain_Derm_ret4 | Russia |
|  | OR936103_Dermacentor_reticulatus_strain_ret2 | Russia |
|  | OM867332_Dermacentor_reticulatus_strain_Derm_ret25 | Russia |
|  | OR936106_Dermacentor_reticulatus_strain_ret5 | Russia |
|  | Cr_112 | Türkiye (CN) |
|  | OR533790_Dermacentor_reticulatus_isolate_South_Kazakhstan1_89_5 | Kazakhstan |
|  | OR936117_Dermacentor_reticulatus_strain_ret4 | Russia |
|  | OR936116_Dermacentor_reticulatus_strain_ret3 | Russia |
|  | OR936118_Dermacentor_reticulatus_strain_ret5 | Russia |
|  | OR162330_Dermacentor_reticulatus_strain_Derm_ret5 | Russia |
|  | OR162323_Dermacentor_reticulatus_strain_Derm_ret3 | Russia |
|  | OR936114_Dermacentor_reticulatus_strain_ret1 | Russia |
|  | OR162328_Dermacentor_reticulatus_strain_Derm_ret3 | Russia |
|  | OR162326_Dermacentor_reticulatus_strain_Derm_ret1 | Russia |
|  | PX672353_Dermacentor_reticulatus_voucher_KZ25876_2 | Kazakhstan |
| HP14 | OR162322_Dermacentor_reticulatus_strain_Derm_ret2 | Russia |
|  | OR162324_Dermacentor_reticulatus_strain_Derm_ret4 | Russia |
|  | OR162321_Dermacentor_reticulatus_strain_Derm_ret1 | Russia |
| HP15 | OR936105_Dermacentor_reticulatus_strain_ret4 | Russia |
|  | OR936104_Dermacentor_reticulatus_strain_ret3 | Russia |
| HP16 | Cr_1317 | Türkiye (CN) |
| HP17 | Cr_1852 | Türkiye (NE) |
| HP18 | Cr_1831 | Türkiye (NE) |
| HP19 | Cr_134 | Türkiye (CN) |
| HP20 | Cr_251 | Türkiye (CN) |
| HP21 | OR936102_Dermacentor_reticulatus_strain_ret1 | Russia |
| HP22 | OR162325_Dermacentor_reticulatus_strain_Derm_ret5 | Russia |
| HP23 | OM867330_Dermacentor_reticulatus_strain_Derm_ret23 | Russia |
| HP24 | OM867318_Dermacentor_reticulatus_strain_Derm_ret13 | Russia |
| HP25 | MT506455_Dermacentor_reticulatus_isolate_H_4C_2 | Kazakhstan |
| HP26 | OM142140_Dermacentor_reticulatus_voucher_9022_haplotype_3 | Czech Republic |
| HP27 | PP048180_Dermacentor_reticulatus_voucher_PHE022017B07 | UK |
| HP28 | OM142147_Dermacentor_reticulatus_voucher_11593_haplotype_10 | Russia |
| HP29 | OM142144_Dermacentor_reticulatus_voucher_33_haplotype_7 | Belarus |
| HP30 | OM142142_Dermacentor_reticulatus_voucher_9787_haplotype_5 | Belarus |
| HP31 | OM142143_Dermacentor_reticulatus_voucher_9746_haplotype_6 | Belarus |
| HP32 | MZ305510_Dermacentor_reticulatus_voucher_CROBB798 | Croatia |
