# Supplementary material for: Spatial Heterogeneity of Tick‐Borne Pathogens Outpaces Genetic Structuring in Anatolian Dermacentor reticulatus Populations
Source: Transbound Emerg Dis. 2026 Jul 22;2026:5552728. doi: 10.1155/tbed/5552728 (PMC13390018; doi:10.1155/tbed/5552728)
Supplement: Supplementary file 10 — Supporting Information 10 Table S10: Geographic distribution and accession numbers of nuclear ITS2 genotypes identified in Dermacentor reticulatus from Anatolia. [file TBED-2026-5552728-s017.docx]

**Supplementary Table 10.** Geographic distribution and accession numbers of nuclear ITS2 genotypes identified in *Dermacentor reticulatus* from Anatolia.

| **Genotype name** | **Study region** | **n** | **L1** | **L2** | **L3** | **L4** | **L7** | **LGr** | **L16** | **L17** | **L18** | **L20** | **L21** | **L22** | **L26** | **GenBank Accession no.** |
| --- | --- | --- | --- | --- | --- | --- | --- | --- | --- | --- | --- | --- | --- | --- | --- | --- |
| Its-CN1 | CN | 2 | 2 |  |  |  |  |  |  |  |  |  |  |  |  | PX789865 |
| Its-CN2 | CN | 1 | 1 |  |  |  |  |  |  |  |  |  |  |  |  | PX789866 |
| Its-CN3 | CN | 4 | 3 |  | 1 |  |  |  |  |  |  |  |  |  |  | PX789867 |
| Its-CN4 | CN | 9 | 9 |  |  |  |  |  |  |  |  |  |  |  |  | PX789871 |
| Its-CN5 | CN | 2 | 2 |  |  |  |  |  |  |  |  |  |  |  |  | PX789875 |
| Its-CN6 | CN | 1 | 1 |  |  |  |  |  |  |  |  |  |  |  |  | PX789876 |
| Its-CN7 | CN | 3 | 1 | 2 |  |  |  |  |  |  |  |  |  |  |  | PX789877 |
| Its-CN8 | CN | 2 |  | 1 |  |  |  | 1 |  |  |  |  |  |  |  | PX789879 |
| Its-CN9 | CN | 1 |  |  |  |  | 1 |  |  |  |  |  |  |  |  | PX789884 |
| Its-NE1 | NE | 1 |  |  |  |  |  |  | 1 |  |  |  |  |  |  | PX789885 |
| Its-NE2 | NE | 1 |  |  |  |  |  |  |  |  | 1 |  |  |  |  | PX789886 |
| Its-NE3 | NE | 1 |  |  |  |  |  |  |  |  |  |  | 1 |  |  | PX789887 |
| Its-NE4 | NE | 2 |  |  |  |  |  |  |  |  |  |  |  | 2 |  | PX789888 |
| Its-NE5 | NE | 1 |  |  |  |  |  |  |  |  |  |  |  | 1 |  | PX789889 |
| Its-NE6 | NE | 1 |  |  |  |  |  |  |  |  |  |  |  |  | 1 | PX789890 |
| Its-NE7 | NE | 1 |  |  |  |  |  |  |  |  |  |  |  |  | 1 | PX789891 |
| Its-CNNE1 | CN+NE | 37 | 15 | 2 | 4 |  | 1 | 2 | 1 | 1 | 1 |  | 4 | 4 | 2 | PX789868 |
| Its-CNNE2 | CN+NE | 5 | 1 | 1 |  |  |  |  |  |  |  |  |  | 2 | 1 | PX789869 |
| Its-CNNE3 | CN+NE | 43 | 13 | 4 | 2 |  | 2 | 3 | 3 | 2 | 2 | 2 | 4 | 3 | 3 | PX789870 |
| Its-CNNE4 | CN+NE | 17 | 2 |  | 4 |  |  | 4 | 1 |  | 1 |  | 2 | 3 |  | PX789872 |
| Its-CNNE5 | CN+NE | 6 | 3 |  |  |  | 1 |  |  |  |  |  | 2 |  |  | PX789873 |
| Its-CNNE6 | CN+NE | 7 | 1 | 2 |  |  | 1 |  | 2 |  | 1 |  |  |  |  | PX789874 |
| Its-CNNE7 | CN+NE | 2 |  | 1 |  |  |  |  |  |  |  |  |  |  | 1 | PX789878 |
| Its-CNNE8 | CN+NE | 2 |  | 1 |  |  |  |  |  |  |  | 1 |  |  |  | PX789880 |
| Its-CNNE9 | CN+NE | 2 |  |  | 1 |  |  |  |  |  |  |  | 1 |  |  | PX789881 |
| Its-CNNE10 | CN+NE | 3 |  |  | 1 |  |  |  |  |  |  |  | 1 | 1 |  | PX789882 |
| Its-CNNE11 | CN+NE | 3 |  |  |  | 1 |  |  | 1 |  | 1 |  |  |  |  | PX789883 |

L1–L26 correspond to study sites as listed in Table 1.

CN: Central Anatolia

NE: Northeast Anatolia
