# Supplementary material for: Spatial Heterogeneity of Tick‐Borne Pathogens Outpaces Genetic Structuring in Anatolian Dermacentor reticulatus Populations
Source: Transbound Emerg Dis. 2026 Jul 22;2026:5552728. doi: 10.1155/tbed/5552728 (PMC13390018; doi:10.1155/tbed/5552728)
Supplement: Supplementary file 11 — Supporting Information 11 Table S11: GenBank reference sequences used in Bayesian phylogenetic analysis based on nuclear ITS2 genotypes of Dermacentor reticulatus. Accession numbers, country of origin, and sequence metadata of D. reticulatus reference sequences retrieved from GenBank and included in the ITS2‐based Bayesian phylogenetic analysis are provided. [file TBED-2026-5552728-s016.docx]

**Supplementary Table 11.** **GenBank reference sequences used in Bayesian phylogenetic analysis based on nuclear ITS2 genotypes of Dermacentor reticulatus.** Accession numbers, country of origin, and sequence metadata of D. reticulatus reference sequences retrieved from GenBank and included in the ITS2-based Bayesian phylogenetic analysis are provided.

| **Genogroup** | **GenBank accession number** | **Organism name** | **Country** |
| --- | --- | --- | --- |
| **Clade1** | OM142151 | Dermacentor_reticulatus_voucher_12054_haplotype_4 | Portugal |
| **Clade1** | KY075899 | Dermacentor_reticulatus_haplotype_2 | Poland |
| **Clade1** | KY075901 | Dermacentor_reticulatus_haplotype_4 | Germany |
| **Clade1** | KY075906 | Dermacentor_reticulatus_haplotype_9 | Poland |
| **Clade1** | KY075902 | Dermacentor_reticulatus_haplotype_5 | Poland |
| **Clade2** | OM142149 | Dermacentor_reticulatus_voucher_9054_haplotype_2 | Czech Republic |
| **Clade2** | KY075900 | Dermacentor_reticulatus_haplotype_3 | Poland |
| **Clade2** | OM142148 | Dermacentor_reticulatus_voucher_12056_haplotype_1 | Portugal |
| **Clade2** | KY075905 | Dermacentor_reticulatus_haplotype_8 | Poland |
| **Clade2** | KY075898 | Dermacentor_reticulatus_haplotype_1 | Poland |
| **Clade2** | OM142152 | Dermacentor_reticulatus_voucher_11904_haplotype_5 | Kazakhstan |
| **Clade2** | KY075904 | Dermacentor_reticulatus_haplotype_7 | Poland |
| **Clade2** | OM142150 | Dermacentor_reticulatus_voucher_9056_haplotype_3 | Czech Republic |
| **Clade2** | KY075903 | Dermacentor_reticulatus_haplotype_6 | Poland |
| **Clade2** | OR428530 | Dermacentor_reticulatus | Poland |
| **Clade2** | S83080 | Dermacentor_reticulatus | N.A. |
| **Clade2** | KY075907 | Dermacentor_reticulatus_haplotype_10 | Poland |
| **Clade2** | PQ685972 | Dermacentor_reticulatus_isolate_Nov21_56_Dret | Russia |
| **Outgroup** | PP456863 | Dermacentor_marginatus_strain_IT_CNNE28 | Türkiye |
| **Outgroup** | PP618825 | Dermacentor_raskemensis_isolate_IT_D1109 | Türkiye |
