# Supplementary material for: Spatial Heterogeneity of Tick‐Borne Pathogens Outpaces Genetic Structuring in Anatolian Dermacentor reticulatus Populations
Source: Transbound Emerg Dis. 2026 Jul 22;2026:5552728. doi: 10.1155/tbed/5552728 (PMC13390018; doi:10.1155/tbed/5552728)
Supplement: Supplementary file 12 — Supporting Information 12 Table S12: List of nuclear ITS2 sequences of Dermacentor reticulatus used for the global genotype network analysis. The table includes haplotype codes (GN1–GN9), sample origin (country and region), GenBank accession numbers, sequence length after trimming (591 bp), and inclusion status (this study vs. GenBank‐derived sequences). [file TBED-2026-5552728-s015.docx]

**Supplementary Table 12. List of nuclear ITS2 sequences of *Dermacentor reticulatus* used for the global genotype network analysis.** The table includes haplotype codes (GN1–GN9), sample origin (country and region), GenBank accession numbers, sequence length after trimming (591 bp), and inclusion status (this study vs. GenBank-derived sequences).

| **Genotype label** | **Sequence name** | **Country** |
| --- | --- | --- |
| GN2 | Ir_211 | Türkiye(CN) |
|  | Ir_Gr_T19 | Türkiye(CN) |
| GN1 | PQ685972_1_Dermacentor_reticulatus_isolate_Nov21_56_Dret | Russia |
|  | OM142149_1_Dermacentor_reticulatus_voucher_9054_haplotype_2 | Czech Republic |
|  | Ir_143 | Türkiye(CN) |
|  | Ir_112 | Türkiye(CN) |
|  | OM142148_1_Dermacentor_reticulatus_voucher_12056_haplotype_1 | Portugal |
|  | Ir_2162 | Türkiye(NE) |
|  | Ir_2177 | Türkiye(NE) |
|  | Ir_721 | Türkiye(CN) |
|  | Ir_117 | Türkiye(CN) |
|  | Ir_142 | Türkiye(CN) |
|  | Ir_1110 | Türkiye(CN) |
|  | KY075900_1_Dermacentor_reticulatus_haplotype_3_Poland | Poland |
|  | Ir_1821 | Türkiye(NE) |
|  | Ir_1832 | Türkiye(NE) |
|  | Ir_1661 | Türkiye(NE) |
|  | Ir_1612 | Türkiye(CN) |
|  | Ir_7101 | Türkiye(CN) |
|  | Ir_2711 | Türkiye(CN) |
|  | Ir_255 | Türkiye(CN) |
|  | Ir_1112 | Türkiye(CN) |
|  | OR428530_1_Dermacentor_reticulatus | Poland |
|  | KY075903_1_Dermacentor_reticulatus_haplotype_6 | Poland |
|  | Ir_324 | Türkiye(CN) |
|  | Ir_2111 | Türkiye(NE) |
|  | OM142150_1_Dermacentor_reticulatus_voucher_9056_haplotype_3 | Czech Republic |
|  | Ir_2211 | Türkiye(NE) |
|  | Ir_22112 | Türkiye(NE) |
|  | Ir_2219 | Türkiye(NE) |
|  | Ir_21232 | Türkiye(NE) |
|  | Ir_21231 | Türkiye(NE) |
|  | Ir_21212 | Türkiye(NE) |
|  | Ir_2112 | Türkiye(NE) |
|  | Ir_1853 | Türkiye(NE) |
|  | Ir_1711 | Türkiye(NE) |
|  | Ir_1616 | Türkiye(NE) |
|  | Ir_Gr_T18 | Türkiye(CN) |
|  | Ir_Gr_M12 | Türkiye(CN) |
|  | Ir_752 | Türkiye(CN) |
|  | Ir_342 | Türkiye(CN) |
|  | Ir_322 | Türkiye(CN) |
|  | Ir_323 | Türkiye(CN) |
|  | Ir_321 | Türkiye(CN) |
|  | Ir_265 | Türkiye(CN) |
|  | Ir_264 | Türkiye(CN) |
|  | Ir_1319 | Türkiye(CN) |
|  | Ir_1316 | Türkiye(CN) |
|  | Ir_1315 | Türkiye(CN) |
|  | Ir_1314 | Türkiye(CN) |
|  | Ir_1313 | Türkiye(CN) |
|  | Ir_172 | Türkiye(CN) |
|  | Ir_1312 | Türkiye(CN) |
|  | Ir_139 | Türkiye(CN) |
|  | Ir_131 | Türkiye(CN) |
|  | Ir_128 | Türkiye(CN) |
|  | Ir_123 | Türkiye(CN) |
|  | Ir_122 | Türkiye(CN) |
|  | Ir_1317 | Türkiye(CN) |
|  | Ir_114 | Türkiye(CN) |
|  | Ir_2222 | Türkiye(NE) |
|  | Ir_22111 | Türkiye(NE) |
|  | Ir_22110 | Türkiye(NE) |
|  | Ir_2173 | Türkiye(NE) |
|  | Ir_2131 | Türkiye(NE) |
|  | Ir_1841 | Türkiye(NE) |
|  | Ir_1613 | Türkiye(NE) |
|  | Ir_Gr_T16 | Türkiye(CN) |
|  | Ir_Gr_Y12 | Türkiye(CN) |
|  | Ir_Gr_H12 | Türkiye(CN) |
|  | Ir_Gr_H11 | Türkiye(CN) |
|  | Ir_3101 | Türkiye(CN) |
|  | Ir_341 | Türkiye(CN) |
|  | Ir_331 | Türkiye(CN) |
|  | Ir_325 | Türkiye(CN) |
|  | Ir_1310 | Türkiye(CN) |
|  | Ir_181 | Türkiye(CN) |
|  | Ir_2214 | Türkiye(NE) |
|  | Ir_2212 | Türkiye(NE) |
|  | KY075899_1_Dermacentor_reticulatus_haplotype_2 | Poland |
|  | OM142151_1_Dermacentor_reticulatus_voucher_12054_haplotype_4 | Portugal |
|  | Ir_2213 | Türkiye(NE) |
|  | Ir_2652 | Türkiye(NE) |
|  | Ir_2657 | Türkiye(NE) |
|  | Ir_126 | Türkiye(CN) |
|  | Ir_111 | Türkiye(CN) |
|  | Ir_2655 | Türkiye(NE) |
|  | Ir_2221 | Türkiye(NE) |
|  | Ir_2217 | Türkiye(NE) |
|  | Ir_253 | Türkiye(CN) |
|  | Ir_174 | Türkiye(CN) |
|  | KY075898_1_Dermacentor_reticulatus_haplotype_1_Poland | Poland |
|  | Ir_1318 | Türkiye(CN) |
|  | Ir_151 | Türkiye(CN) |
|  | Ir_153 | Türkiye(CN) |
|  | Ir_161 | Türkiye(CN) |
|  | Ir_163 | Türkiye(CN) |
|  | Ir_118 | Türkiye(CN) |
|  | Ir_115 | Türkiye(CN) |
|  | Ir_116 | Türkiye(CN) |
|  | Ir_119 | Türkiye(CN) |
|  | Ir_132 | Türkiye(CN) |
|  | Ir_136 | Türkiye(CN) |
|  | Ir_1311 | Türkiye(CN) |
|  | Ir_141 | Türkiye(CN) |
|  | Ir_251 | Türkiye(CN) |
|  | Ir_2716 | Türkiye(CN) |
|  | Ir_2712 | Türkiye(CN) |
|  | Ir_261 | Türkiye(CN) |
|  | Ir_391 | Türkiye(CN) |
|  | Ir_3104 | Türkiye(CN) |
|  | Ir_761 | Türkiye(CN) |
|  | Ir_7121 | Türkiye(CN) |
|  | Ir_Gr_M11 | Türkiye(CN) |
|  | Ir_Gr_Y11 | Türkiye(CN) |
|  | Ir_Gr_T110 | Türkiye(CN) |
|  | Ir_1614 | Türkiye(NE) |
|  | Ir_1615 | Türkiye(NE) |
|  | Ir_1621 | Türkiye(NE) |
|  | Ir_1721 | Türkiye(NE) |
|  | Ir_1712 | Türkiye(NE) |
|  | Ir_1831 | Türkiye(NE) |
|  | Ir_1851 | Türkiye(NE) |
|  | Ir_2043 | Türkiye(NE) |
|  | Ir_2042 | Türkiye(NE) |
|  | Ir_2132 | Türkiye(NE) |
|  | Ir_2164 | Türkiye(NE) |
|  | Ir_2121 | Türkiye(NE) |
|  | Ir_21211 | Türkiye(NE) |
|  | Ir_2215 | Türkiye(NE) |
|  | Ir_2216 | Türkiye(NE) |
|  | Ir_2223 | Türkiye(NE) |
|  | Ir_2621 | Türkiye(NE) |
|  | Ir_2651 | Türkiye(NE) |
|  | Ir_2653 | Türkiye(NE) |
|  | OM142152_1_Dermacentor_reticulatus_voucher_11904_haplotype_5 | Kazakhstan |
|  | Ir_22114 | Türkiye(NE) |
|  | Ir_2171 | Türkiye(CN) |
|  | Ir_3103 | Türkiye(CN) |
|  | KY075904_1_Dermacentor_reticulatus_haplotype_7 | Poland |
|  | Ir_262 | Türkiye(CN) |
|  | Ir_2656 | Türkiye(NE) |
|  | Ir_2654 | Türkiye(NE) |
|  | Ir_138 | Türkiye(CN) |
|  | Ir_135 | Türkiye(CN) |
|  | Ir_134 | Türkiye(CN) |
|  | Ir_133 | Türkiye(CN) |
|  | Ir_129 | Türkiye(CN) |
|  | Ir_127 | Türkiye(CN) |
|  | Ir_125 | Türkiye(CN) |
|  | Ir_124 | Türkiye(CN) |
|  | Ir_121 | Türkiye(CN) |
|  | KY075906_1_Dermacentor_reticulatus_haplotype_9 | Poland |
|  | Ir_241 | Türkiye(CN) |
|  | Ir_2041 | Türkiye(NE) |
|  | Ir_1111 | Türkiye(CN) |
|  | Ir_263 | Türkiye(CN) |
|  | Ir_271 | Türkiye(CN) |
|  | Ir_3102 | Türkiye(CN) |
|  | Ir_182 | Türkiye(CN) |
|  | Ir_137 | Türkiye(CN) |
|  | Ir_113 | Türkiye(CN) |
|  | KY075902_1_Dermacentor_reticulatus_haplotype_5 | Poland |
|  | Ir_411 | Türkiye(CN) |
|  | Ir_1631 | Türkiye(NE) |
|  | Ir_1852 | Türkiye(NE) |
| GN3 | Ir_2218 | Türkiye(NE) |
|  | Ir_751 | Türkiye(CN) |
| GN4 | Ir_191 | Türkiye(CN) |
|  | Ir_197 | Türkiye(CN) |
| GN5 | Ir_1611 | Türkiye(NE) |
|  | Ir_2611 | Türkiye(NE) |
| GN6 | KY075905_1_Dermacentor_reticulatus_haplotype_8 | Poland |
|  | KY075907_1_Dermacentor_reticulatus_haplotype_10 | Poland |
| GN7 | Ir_1113 | Türkiye(CN) |
| GN8 | Ir_2175 | Türkiye(NE) |
| GN9 | KY075901_1_Dermacentor_reticulatus_haplotype_4 | Germany |
