# Supplementary figures and images for: Spatial Heterogeneity of Tick‐Borne Pathogens Outpaces Genetic Structuring in Anatolian Dermacentor reticulatus Populations
Source: Transbound Emerg Dis. 2026 Jul 22;2026:5552728. doi: 10.1155/tbed/5552728 (PMC13390018; doi:10.1155/tbed/5552728)

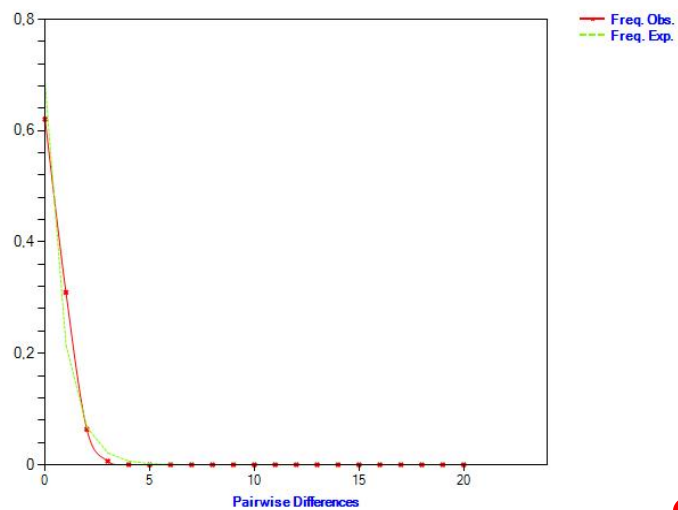

CN

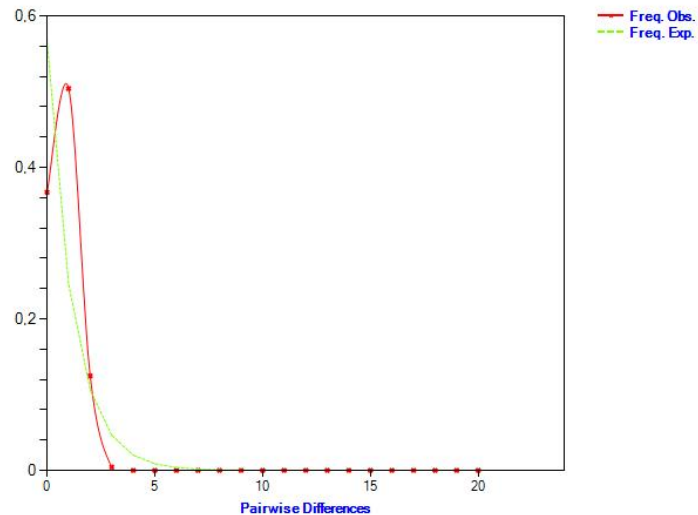

NE

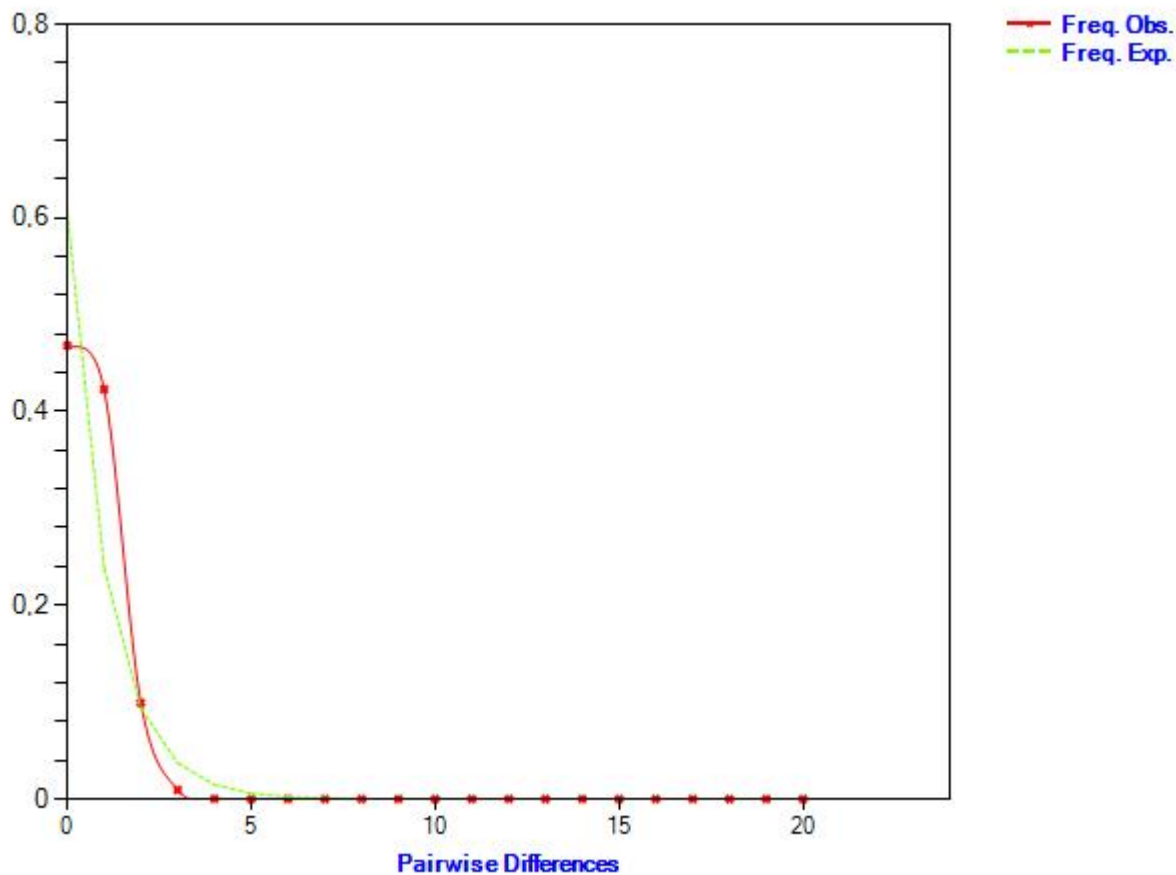

ALL

Supplement: Supplementary file 13 — Supporting Information 13 Figure S1: Mismatch distribution based on mitochondrial cox1 sequences of Dermacentor reticulatus from Anatolia. Observed (solid line) and expected (dashed line) distributions of pairwise nucleotide differences are shown separately for Central Anatolia (CN), Northeastern Anatolia (NE), and the pooled dataset (ALL). All datasets exhibit unimodal distributions with low raggedness, indicating limited mitochondrial sequence divergence among haplotypes. [file TBED-2026-5552728-s014.pdf]

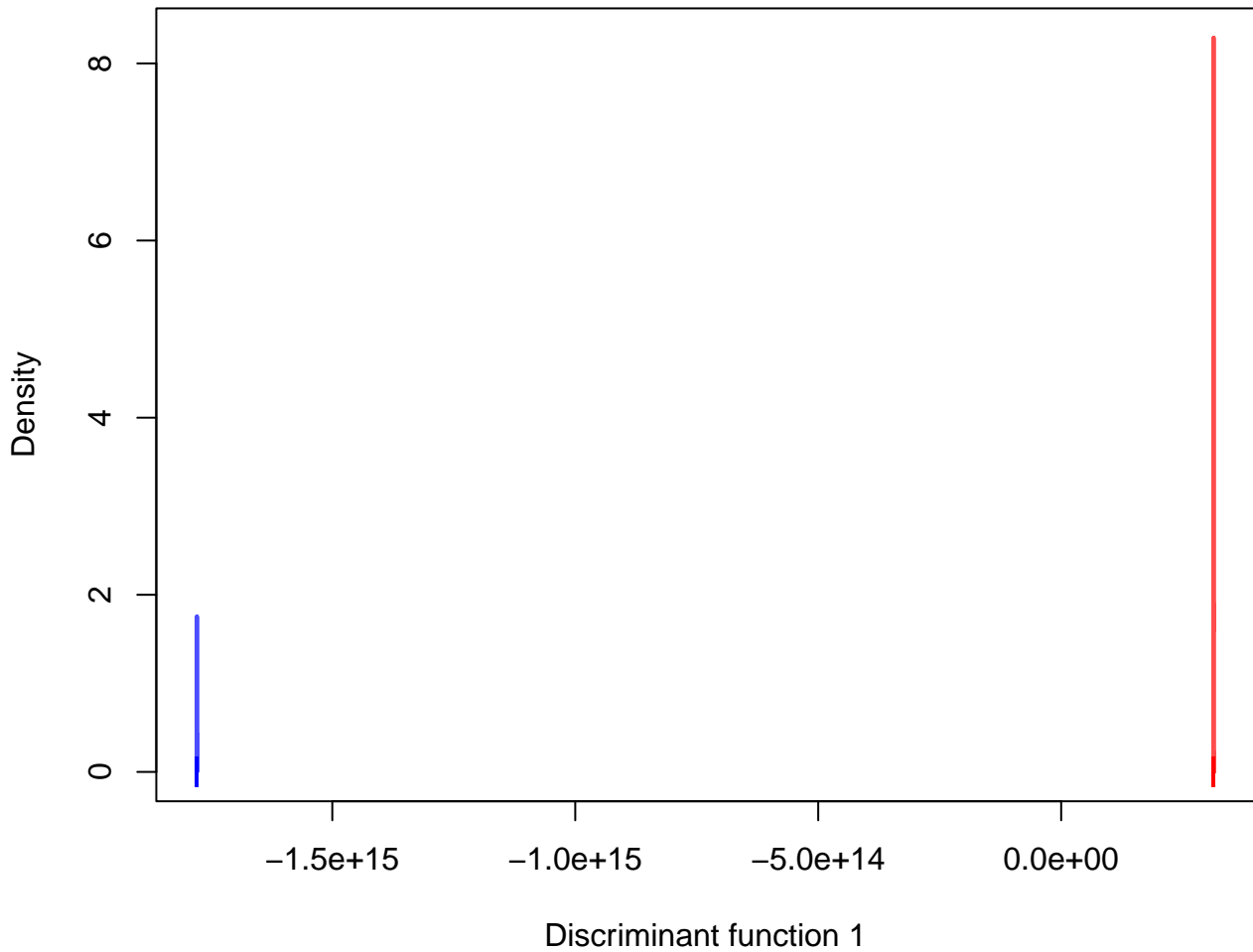

Supplement: Supplementary file 14 — Supporting Information 14 Figure S2: Unsupervised discriminant analysis of principal components (DAPC) based on mitochondrial cox1 sequences of Dermacentor reticulatus. Clustering was performed without a priori population assignment (k = 2). The scatter plot shows extensive overlap between inferred clusters, indicating weak genetic structure in the absence of predefined populations. [file TBED-2026-5552728-s013.pdf]

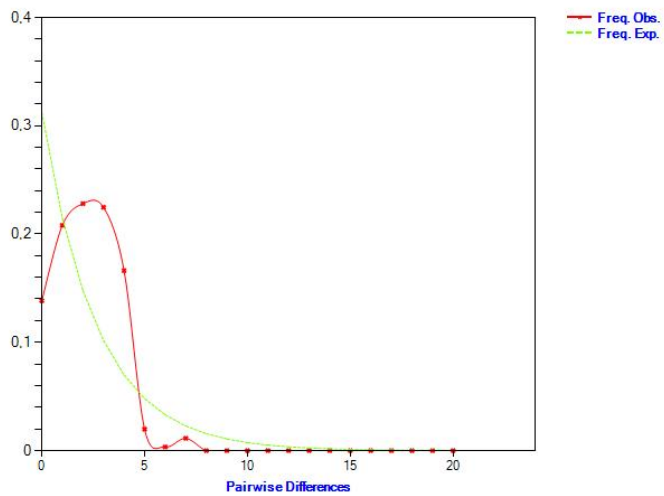

CN

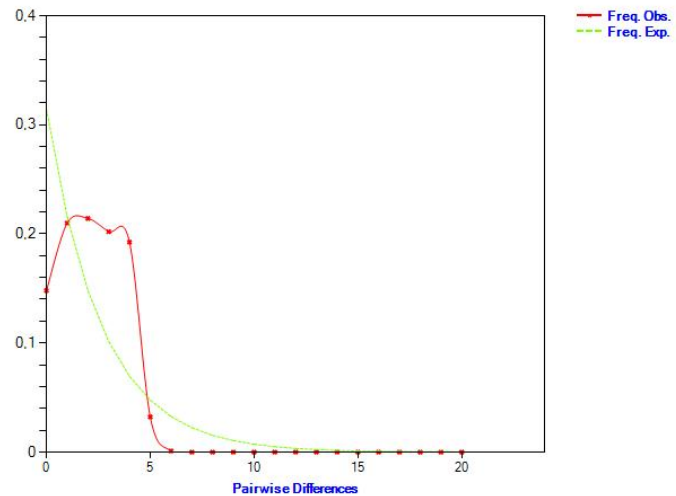

NE

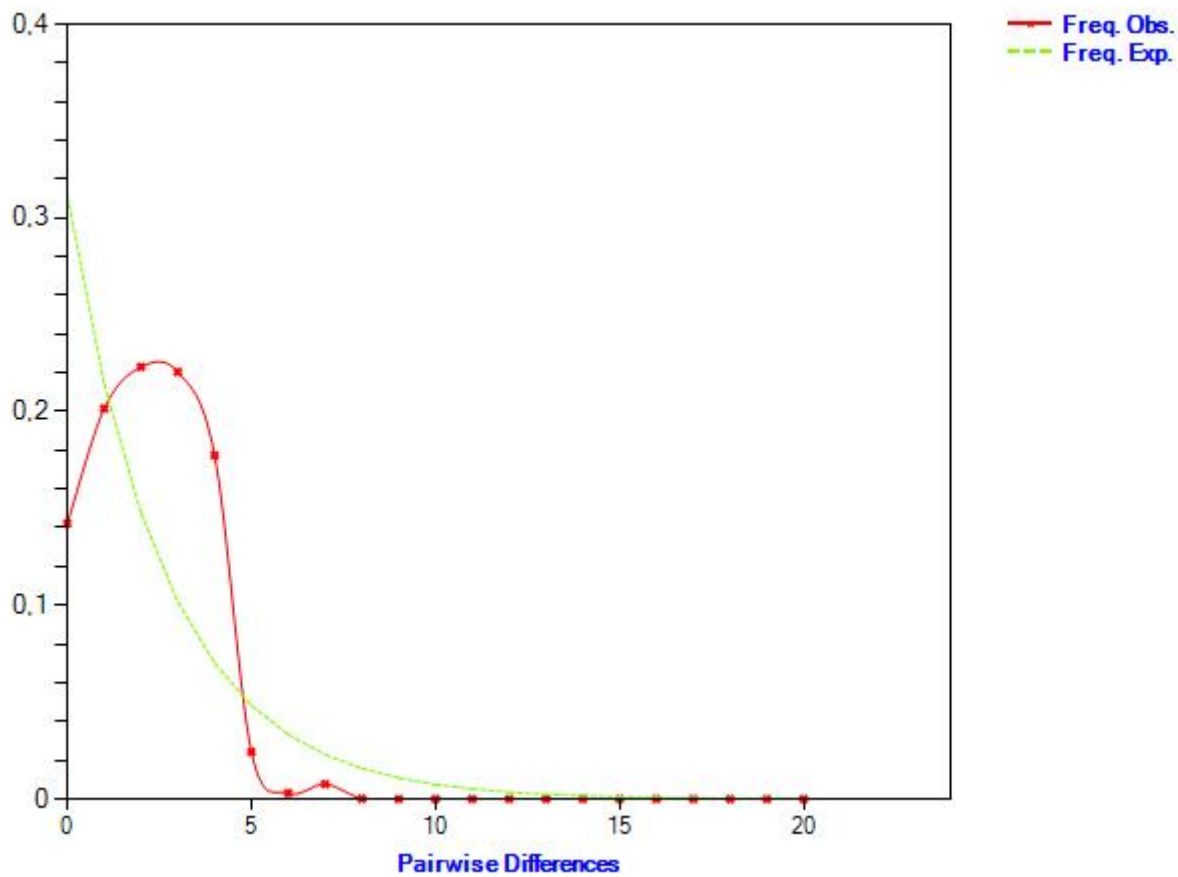

ALL

Supplement: Supplementary file 15 — Supporting Information 15 Figure S3: Mismatch distribution based on nuclear ITS2 sequences of Dermacentor reticulatus. Observed and expected mismatch distributions are shown for the pooled dataset and regional populations, indicating a unimodal pattern consistent with limited nuclear genetic differentiation. [file TBED-2026-5552728-s012.pdf]

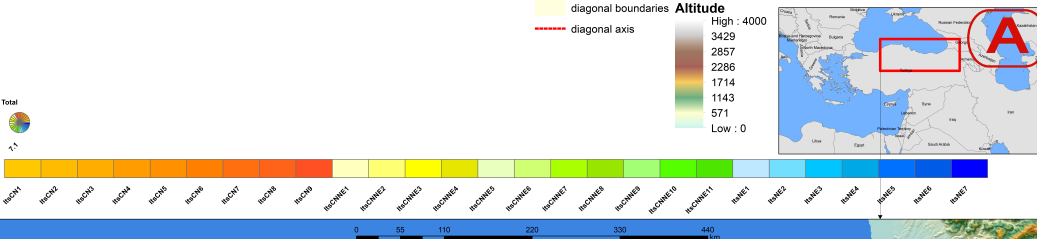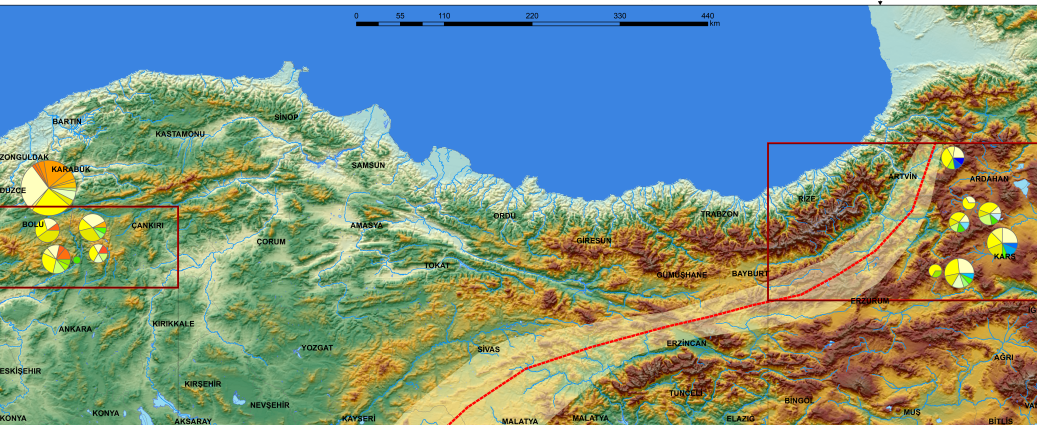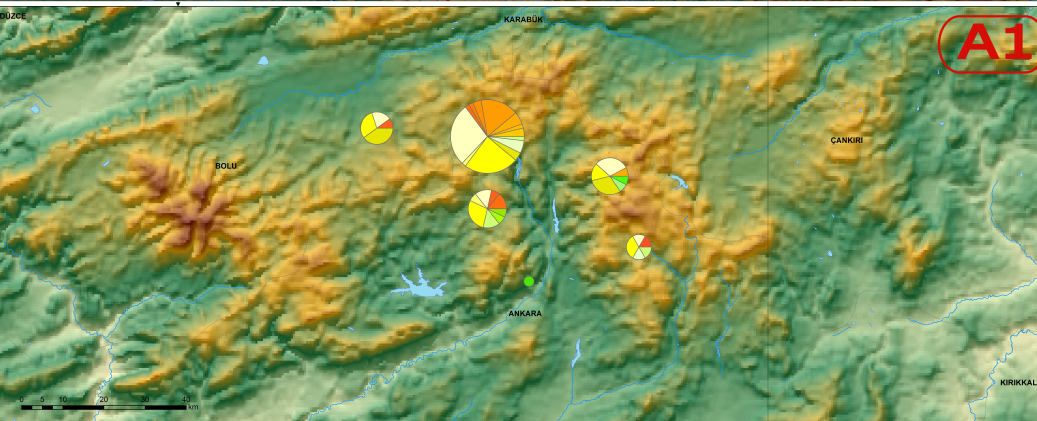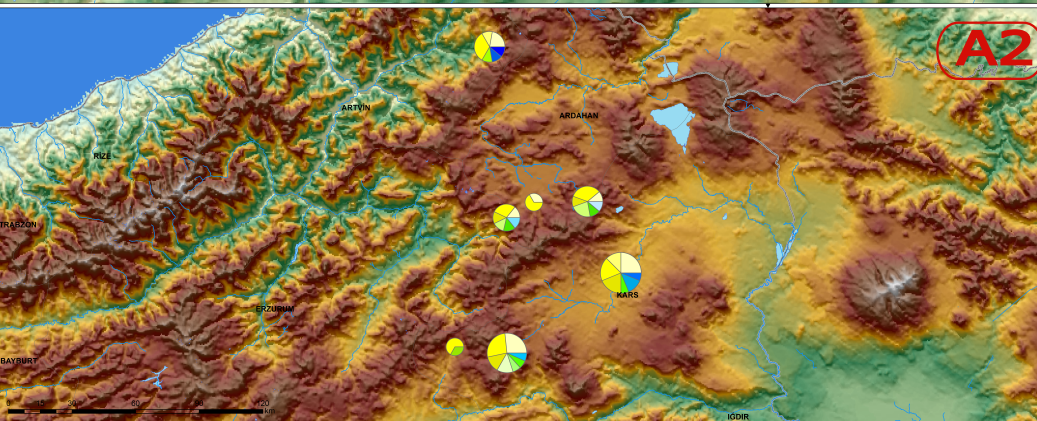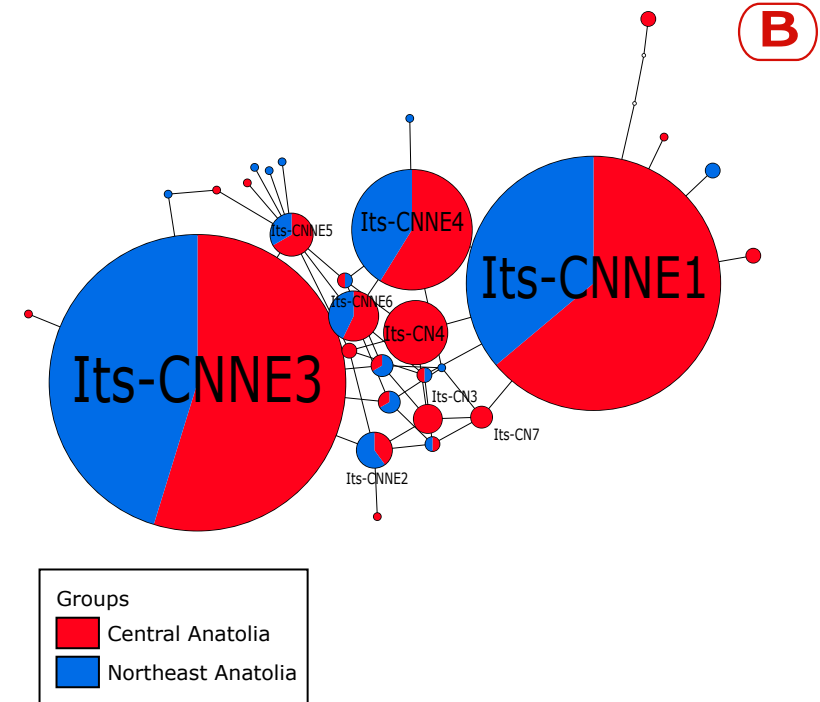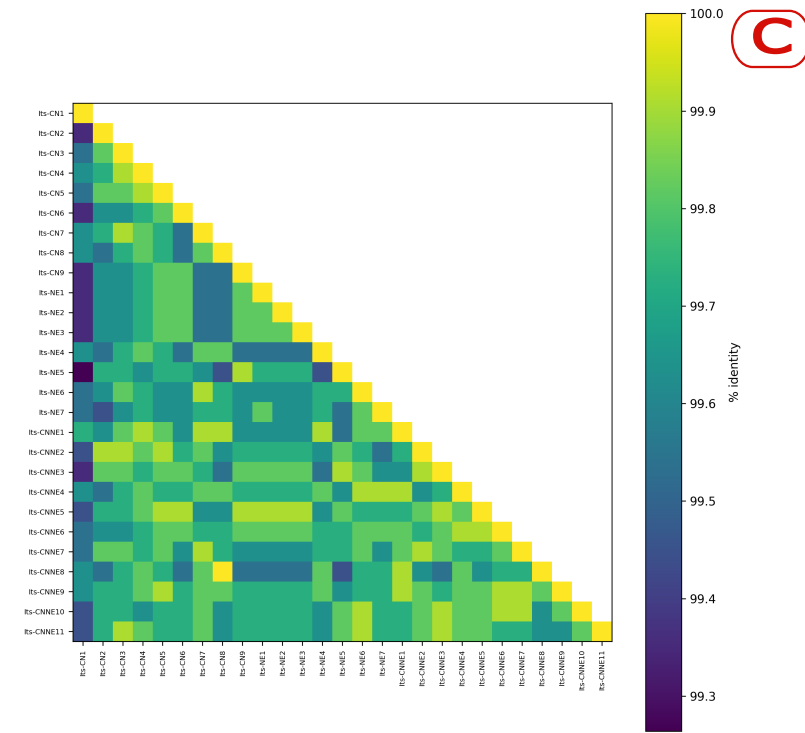

Supplement: Supplementary file 16 — Supporting Information 16 Figure S4: Nuclear ITS2 genotype structure of Dermacentor reticulatus (n = 160) from Anatolia. (A) Geographic distribution of ITS2 genotypes across sampling localities. The upper panel shows the overall study area, with enlargements of (A1) Central Anatolia and (A2) Northeastern Anatolia. Pie charts represent genotype composition at each site. Colors denote regional affiliation: Central Anatolia (red shades), Northeastern Anatolia (blue shades), and shared genotypes (yellow–green shades). (B) TCS genotype network inferred from ITS2 data. Circle size is proportional to genotype frequency, and each connection represents a single mutational step unless otherwise indicated. Colors correspond to geographic origin (Central Anatolia = red; Northeastern Anatolia = blue). (C) Color‐coded matrix of pairwise nucleotide identity among ITS2 haplotypes. [file TBED-2026-5552728-s011.pdf]

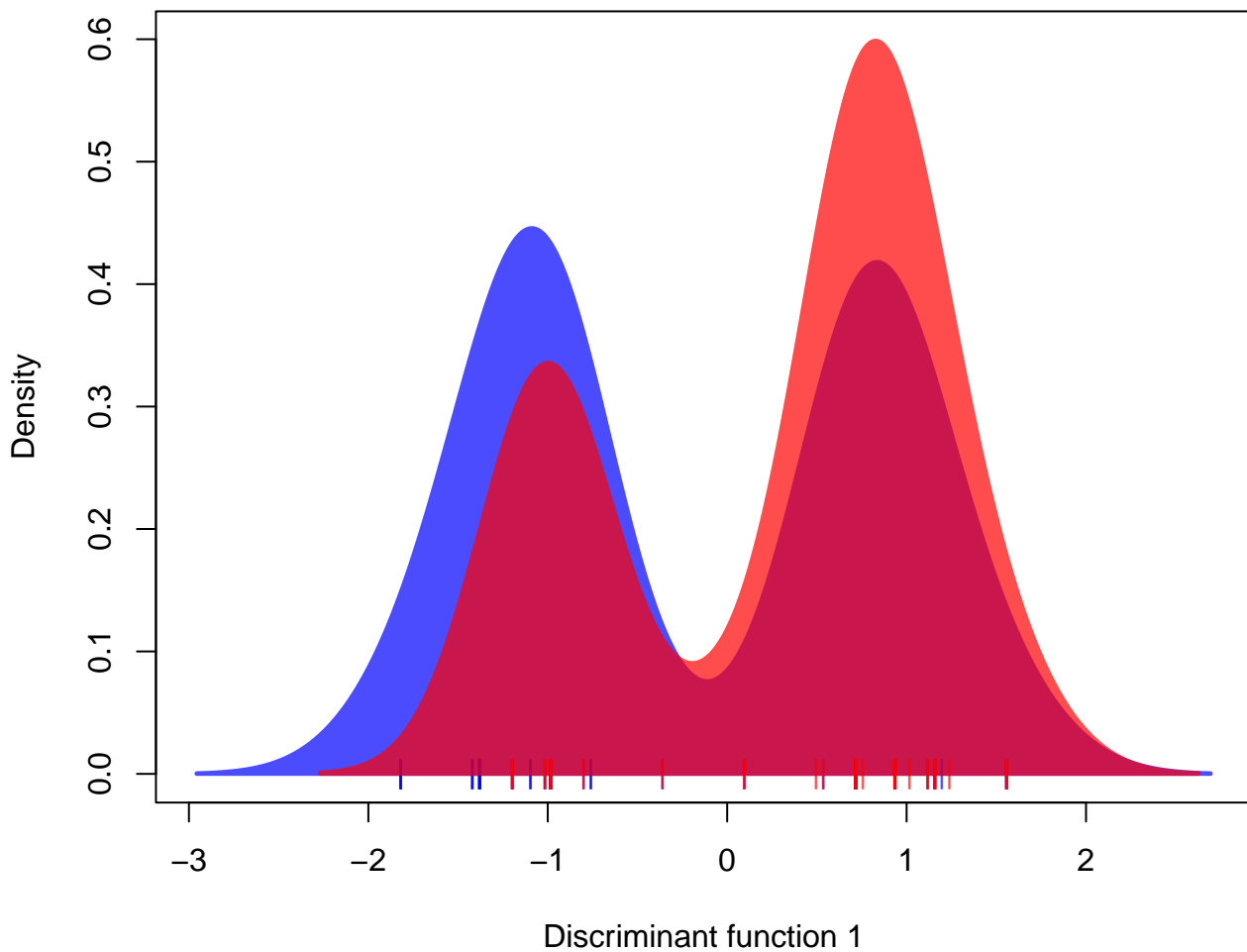

Supplement: Supplementary file 17 — Supporting Information 17 Figure S5: Supervised discriminant analysis of principal components (DAPC) based on nuclear ITS2 sequences of Dermacentor reticulatus. Individuals were assigned a priori to Central Anatolia (CN) and Northeastern Anatolia (NE) populations. The analysis shows extensive overlap between regions, indicating weak nuclear population structure. [file TBED-2026-5552728-s010.pdf]

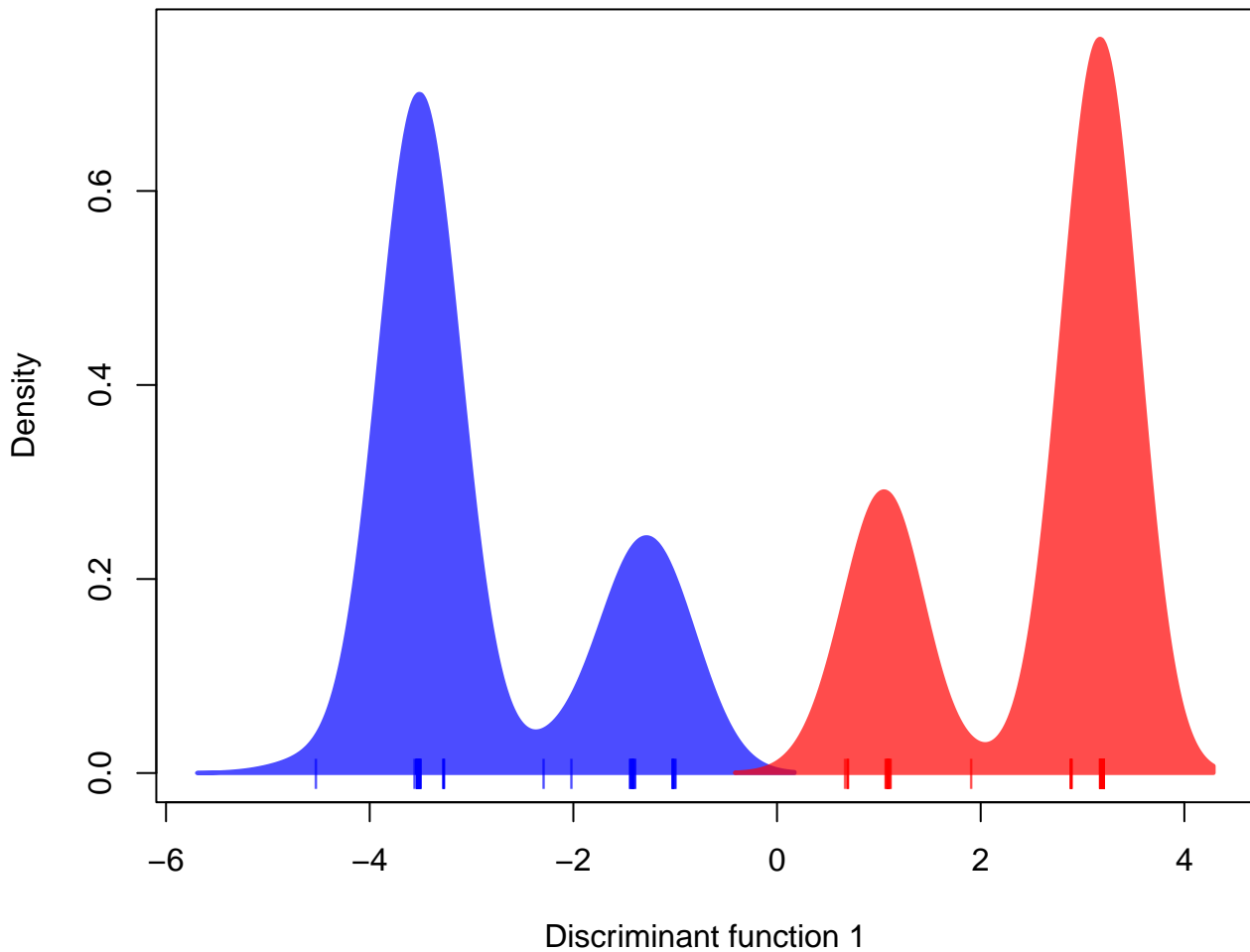

Supplement: Supplementary file 18 — Supporting Information 18 Figure S6: Unsupervised discriminant analysis of principal components (DAPC) based on nuclear ITS2 sequences of Dermacentor reticulatus. Clustering was performed without a priori population assignment (k = 2). The scatter plot shows extensive overlap between inferred clusters, indicating weak nuclear genetic structure in the absence of predefined populations. [file TBED-2026-5552728-s009.pdf]

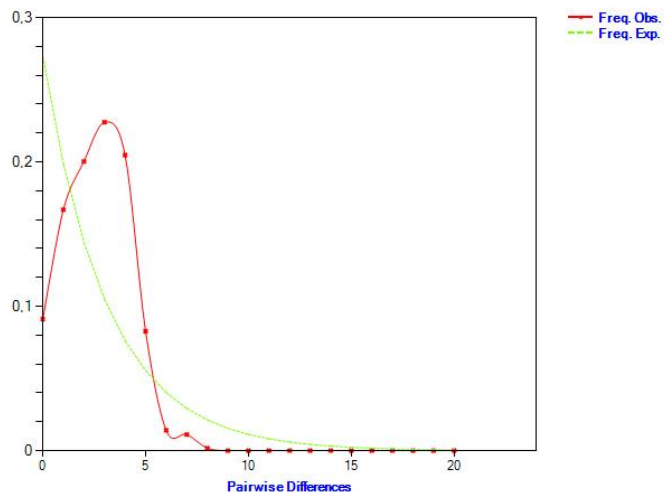

CN

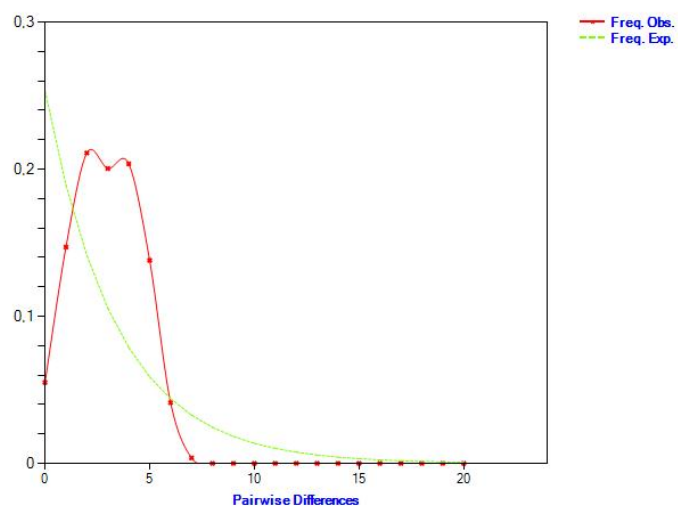

NE

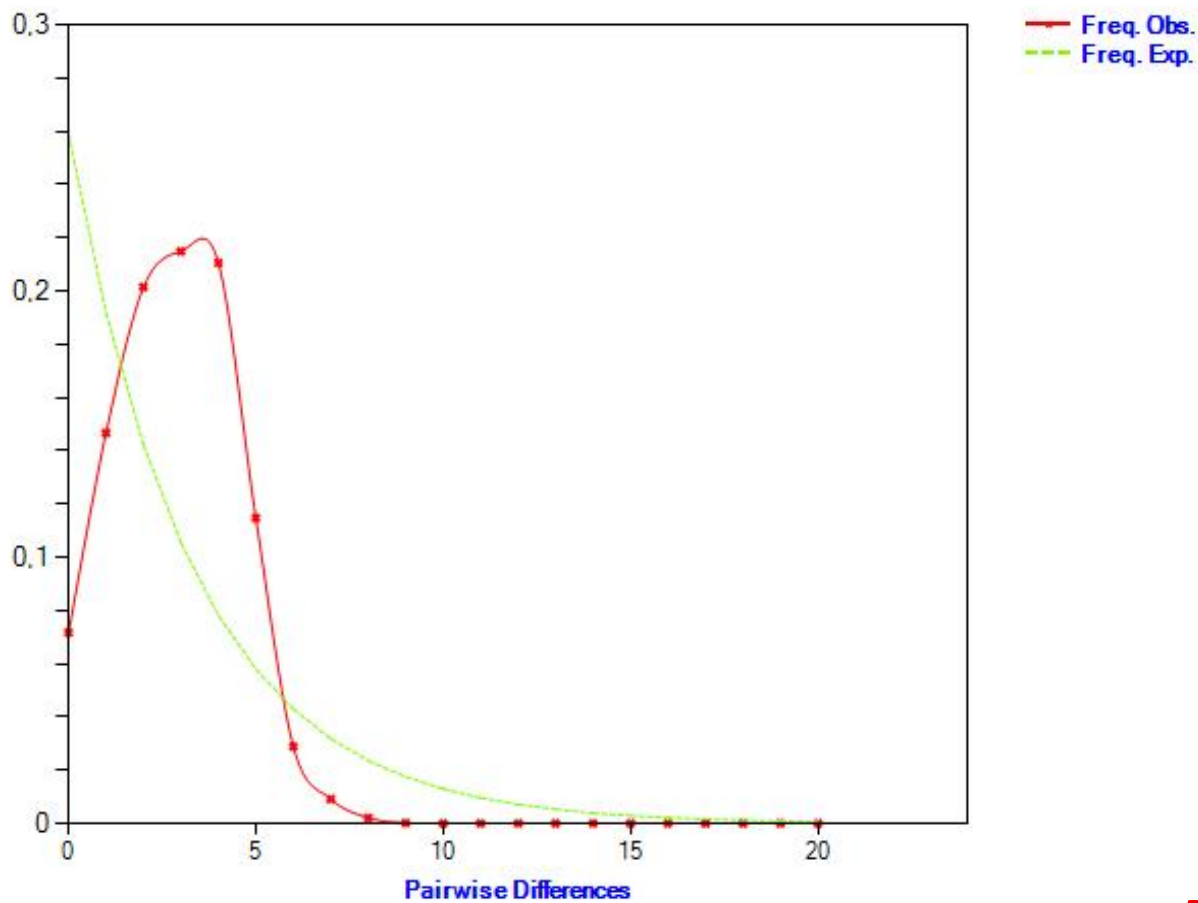

ALL

Supplement: Supplementary file 19 — Supporting Information 19 Figure S7: Mismatch distribution based on concatenated mitochondrial cox1 and nuclear ITS2 sequences of Dermacentor reticulatus. Observed and expected mismatch distributions are shown for the pooled dataset and regional populations, indicating unimodal patterns with low raggedness. [file TBED-2026-5552728-s008.pdf]

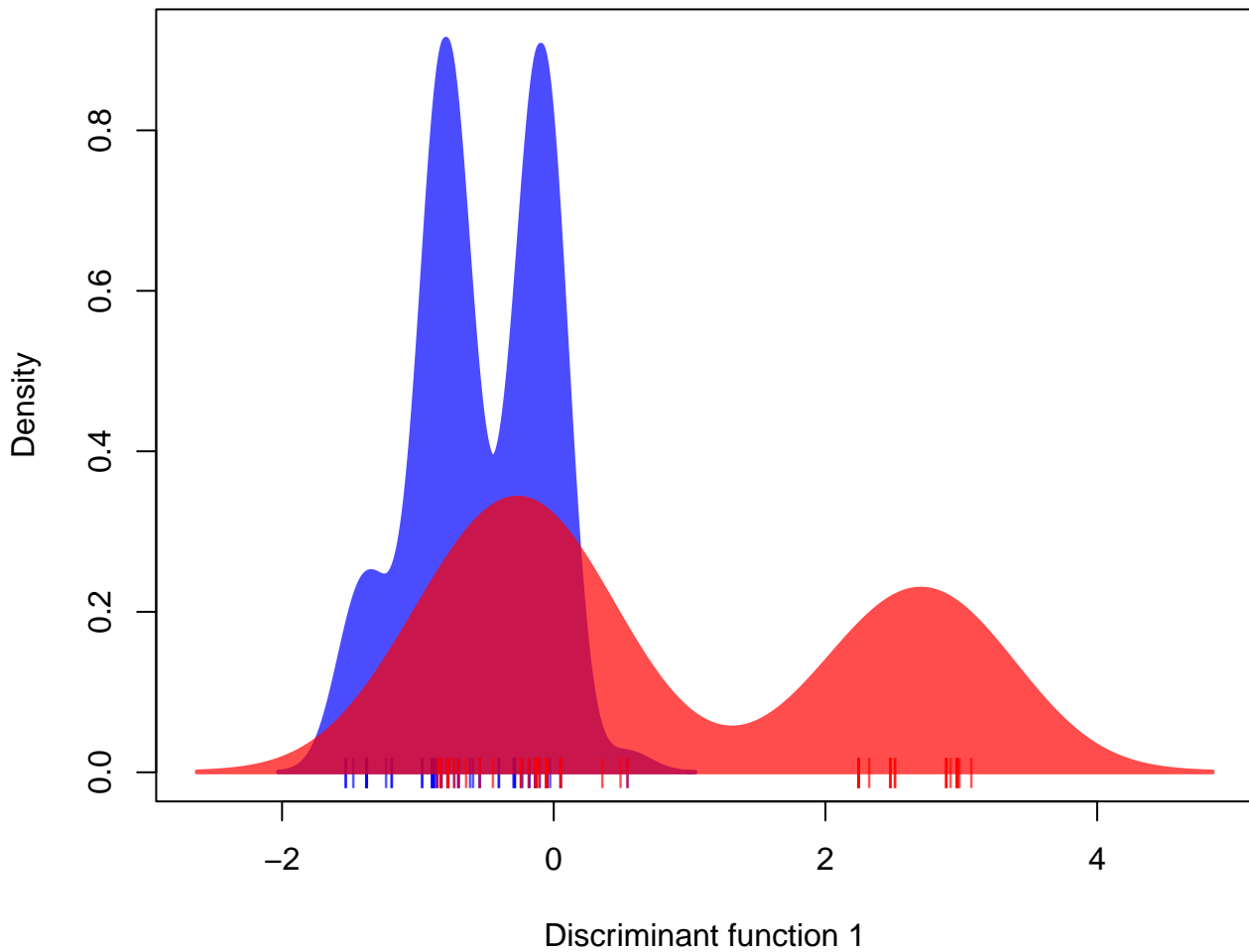

Supplement: Supplementary file 21 — Supporting Information 21 Figure S9: Supervised discriminant analysis of principal components (DAPC) based on concatenated mitochondrial cox1 and nuclear ITS2 sequences of Dermacentor reticulatus. Individuals were assigned a priori to Central Anatolia (CN) and Northeastern Anatolia (NE) populations. The analysis shows partial overlap between regions, indicating weak to moderate multilocus population structure. [file TBED-2026-5552728-s006.pdf]

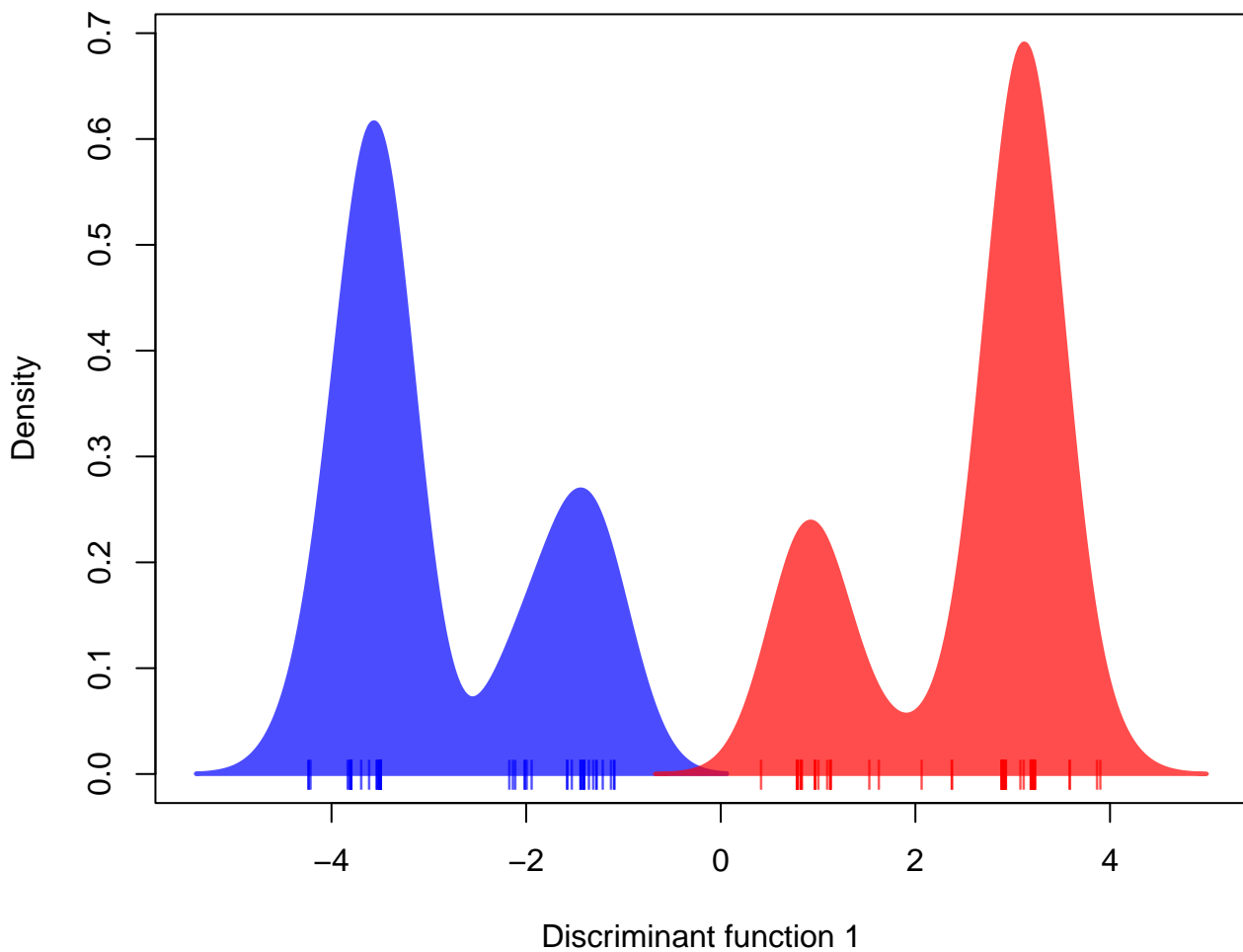

Supplement: Supplementary file 22 — Supporting Information 22 Figure S10: Unsupervised discriminant analysis of principal components (DAPC) based on concatenated mitochondrial cox1 and nuclear ITS2 sequences of Dermacentor reticulatus. Clustering was performed without a priori population assignment (k = 2). The scatter plot shows extensive overlap between inferred clusters, supporting weak multilocus genetic structure. [file TBED-2026-5552728-s005.pdf]

**a**

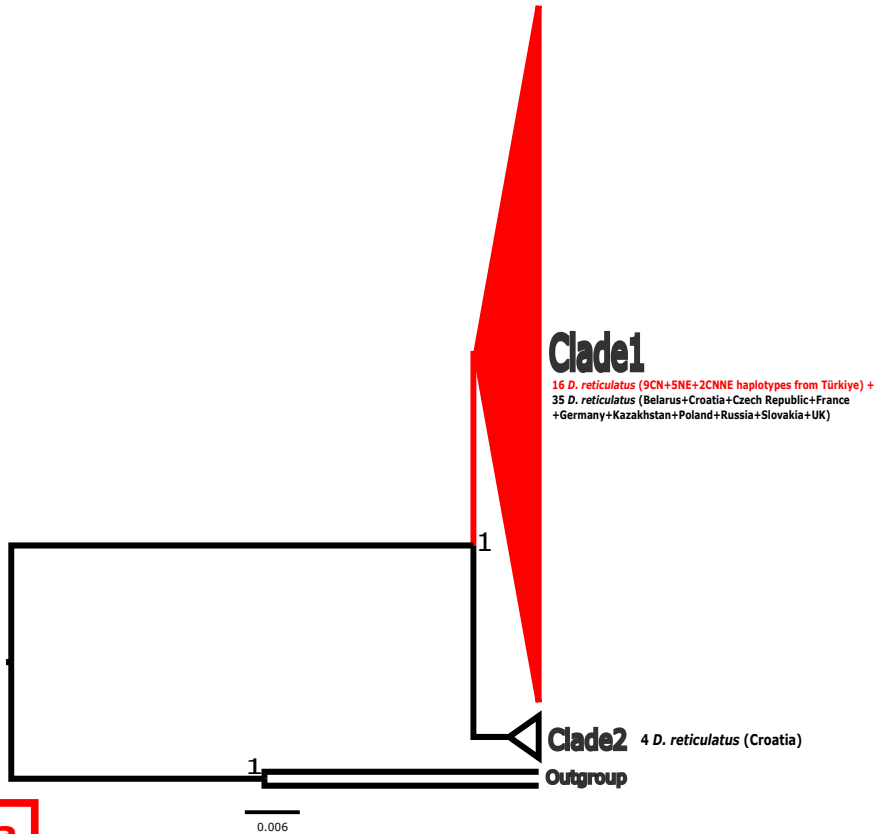

**b**

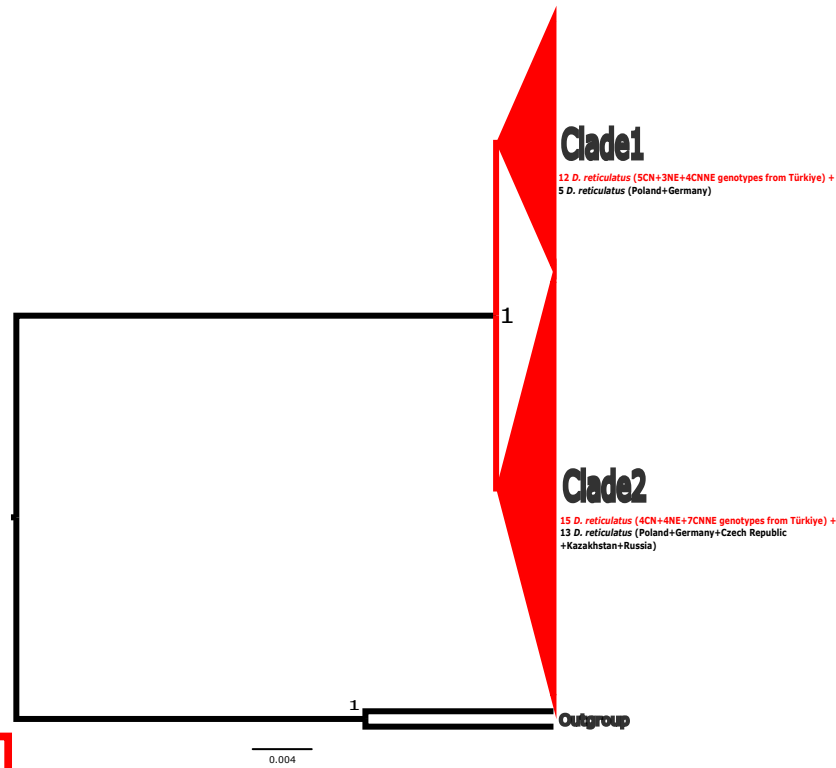

Supplement: Supplementary file 23 — Supporting Information 23 Figure S11: Bayesian phylogenetic placement of mitochondrial cox1 haplotypes and nuclear ITS2 genotypes of Dermacentor reticulatus. Bayesian phylogenetic trees were inferred using BEAST2 based on (a) mitochondrial cox1 haplotypes and (b) nuclear ITS2 genotypes identified in this study together with reference sequences retrieved from GenBank. Branches with posterior probabilities below 0.8 were collapsed. Posterior probability values are shown at major nodes. Dermacentor marginatus and Dermacentor raskemensis sequences were used as outgroups. [file TBED-2026-5552728-s001.pdf]

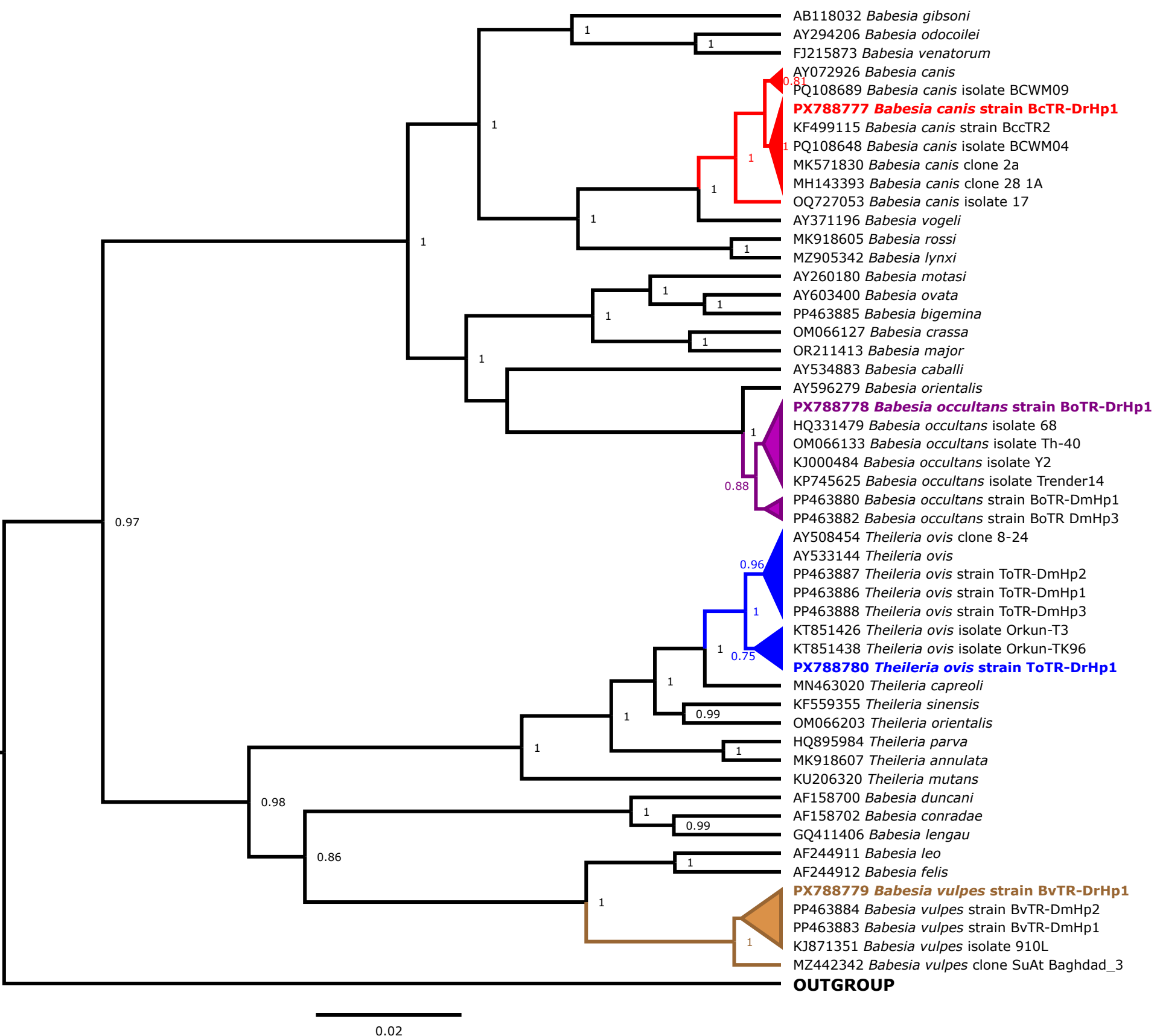

Supplement: Supplementary file 26 — Supporting Information 26 Figure S14: Phylogenetic tree constructed using Bayesian inference based on aligned nucleotide sequences of the 18S rRNA gene of piroplasms, with Cardiosporidium cionae (EU052685) as the outgroup, under the TN93+I+G substitution model. The analysis included 53 sequences and 1,670 positions. Node labels indicate posterior probabilities, with values below 0.75 omitted. Haplotype sequences obtained in this study are highlighted in colors. GenBank® accession numbers are provided before species names. The scale bar represents nucleotide substitutions per site. [file TBED-2026-5552728-s004.pdf]

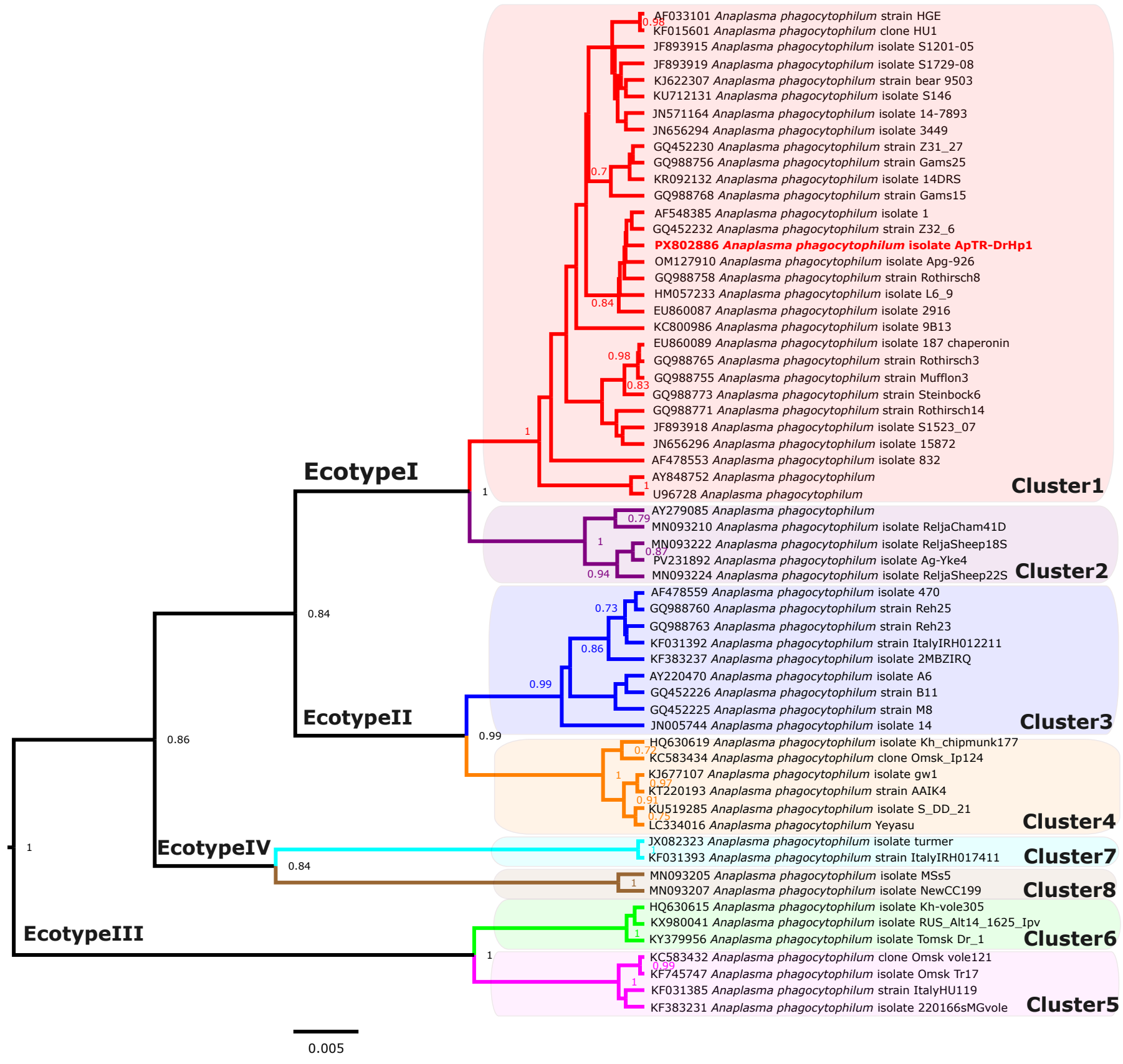

Supplement: Supplementary file 27 — Supporting Information 27 Figure S15: Phylogenetic tree constructed using Bayesian inference based on aligned nucleotide sequences of the groEL gene of Anaplasma phagocytophilum under the HKY+G substitution model. The analysis included 61 sequences and 530 positions. Node labels indicate posterior probabilities, with values below 0.7 omitted. The haplotype sequence obtained in this study and its corresponding clade (Ecotype 1/Cluster 1) are highlighted in red. GenBank® accession numbers are provided before species names. The scale bar represents nucleotide substitutions per site. [file TBED-2026-5552728-s024.pdf]
