# Supplementary material for: Spatial Heterogeneity of Tick‐Borne Pathogens Outpaces Genetic Structuring in Anatolian Dermacentor reticulatus Populations
Source: Transbound Emerg Dis. 2026 Jul 22;2026:5552728. doi: 10.1155/tbed/5552728 (PMC13390018; doi:10.1155/tbed/5552728)

HP1 = Türkiye(CN+NE)+Russia
HP2 = Türkiye(CN)+Russia+Kazakhstan
HP3 = UK+Croatia+Slovaca+Czech Republic+Poland+Germany+Russia
HP4 = Türkiye(NE)
HP5 = Türkiye(CN)
HP6 = Russia
HP7 = Türkiye(NE)
HP8 = Russia
HP9 = Türkiye(NE)
HP10 = Türkiye(CN)
HP11 = Czech Republic+Germany
HP12 = Czech Republic
HP13 = Türkiye(CN)
HP14 = Russia
HP15 = Russia
HP16 = Türkiye(CN)
HP17 = Türkiye(NE)
HP18 = Türkiye(NE)
HP19 = Türkiye(CN)
HP20 = Türkiye(CN)
HP21 = Russia
HP22 = Russia
HP23 = Russia
HP24 = Russia
HP25 = Kazakhstan
HP26 = Czech Republic
HP27 = UK
HP28 = Russia
HP29 = Belarus
HP30 = Belarus
HP31 = Belarus
HP32 = Croatia

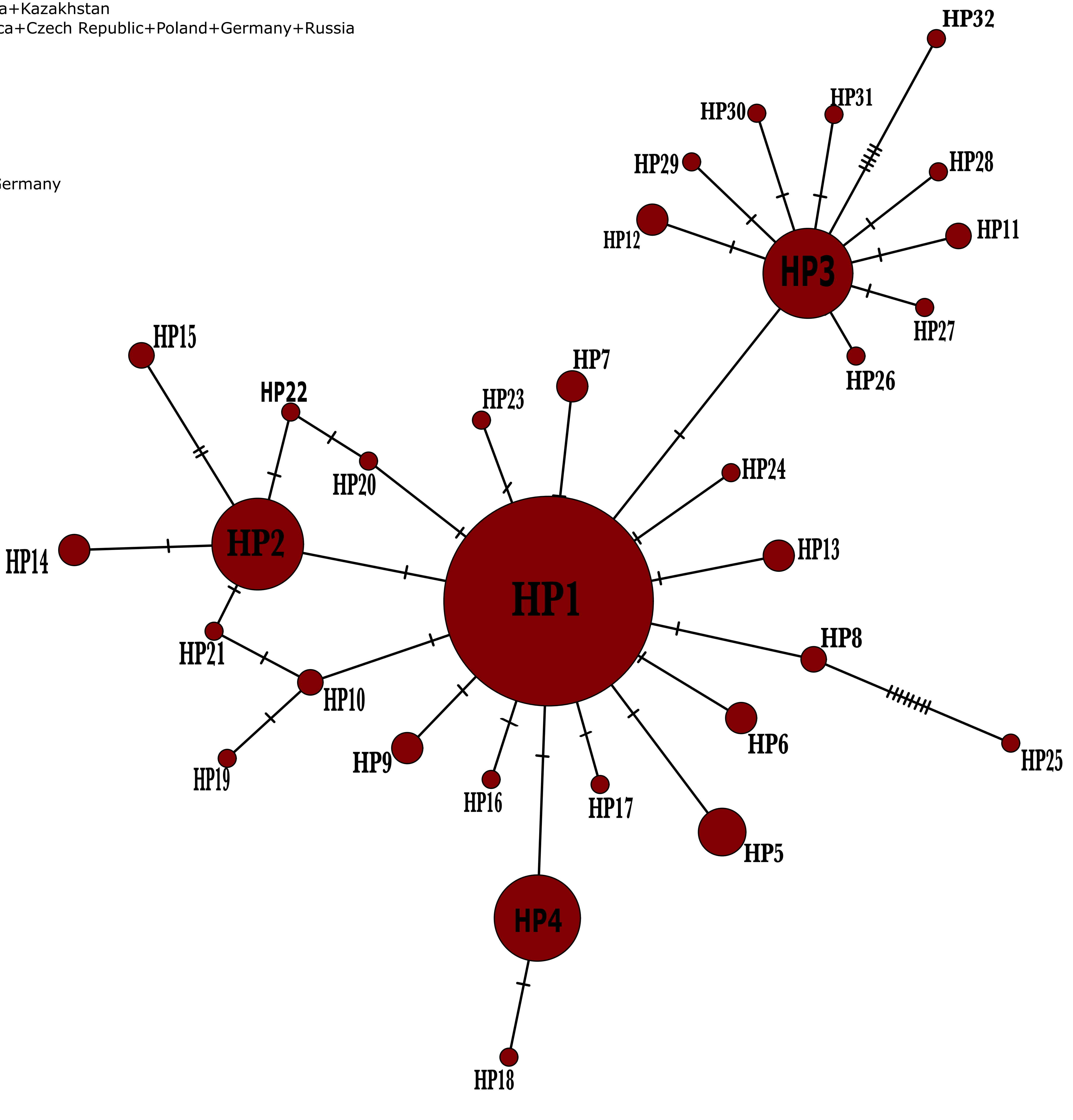

Supplement: Supplementary file 24 — Supporting Information 24 Figure S12: Global haplotype network of Dermacentor reticulatus based on mitochondrial cox1 sequences. The network was constructed using the TCS algorithm and includes 260 sequences (160 from this study and 100 GenBank‐derived sequences with known geographic origin), trimmed to a common length of 606 bp. A total of 32 haplotypes (HP1–HP32) were identified. Circle sizes are proportional to haplotype frequencies and each connecting line represents a single mutational step. Haplotypes detected in this study are highlighted, and their distribution across Central Anatolia (CN) and Northeastern Anatolia (NE) is indicated. The list of countries corresponding to each haplotype is provided alongside the network. [file TBED-2026-5552728-s002.pdf]
