# Supplementary material for: Spatial Heterogeneity of Tick‐Borne Pathogens Outpaces Genetic Structuring in Anatolian Dermacentor reticulatus Populations
Source: Transbound Emerg Dis. 2026 Jul 22;2026:5552728. doi: 10.1155/tbed/5552728 (PMC13390018; doi:10.1155/tbed/5552728)

GN1 = Türkiye(CN+NE)+Russia+Kazakhstan+Czech Republic+Portugal+Poland
GN2 = Türkiye(CN)
GN3 = Türkiye(CN+NE)
GN4 = Türkiye(CN)
GN5 = Türkiye(NE)
GN6 = Poland
GN7 = Türkiye(CN)
GN8 = Türkiye(NE)
GN9 = Germany

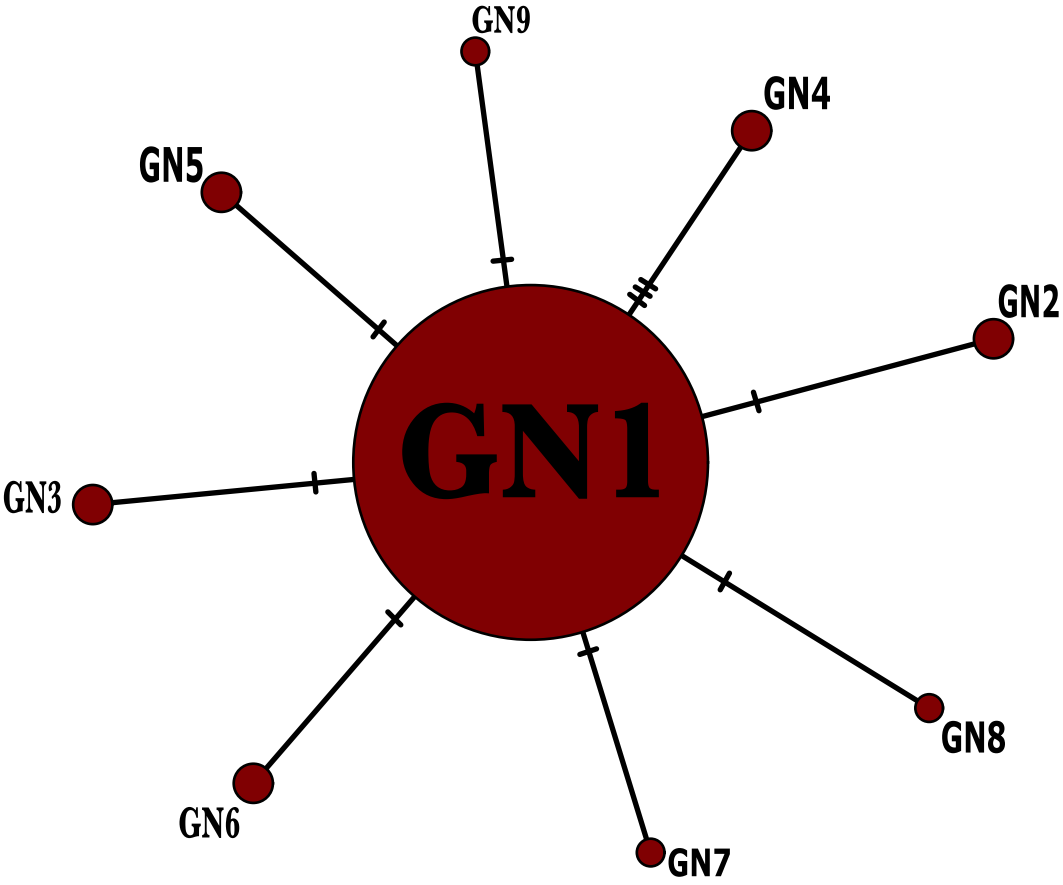

Supplement: Supplementary file 25 — Supporting Information 25 Figure S13: Global genotype network of Dermacentor reticulatus based on nuclear ITS2 sequences. The network was constructed using the TCS algorithm and includes 177 sequences (160 from this study and 17 GenBank‐derived sequences with known geographic origin), trimmed to a common length of 591 bp. A total of 9 genotypes (GN1–GN9) were identified. Circle sizes are proportional to genotype frequencies and each connecting line represents a single mutational step. Genotypes detected in this study are highlighted, and their distribution across Central Anatolia (CN) and Northeastern Anatolia (NE) is indicated. The list of countries corresponding to each genotype is provided alongside the network. [file TBED-2026-5552728-s003.pdf]
