# Supplementary material for: Spatial Heterogeneity of Tick‐Borne Pathogens Outpaces Genetic Structuring in Anatolian Dermacentor reticulatus Populations
Source: Transbound Emerg Dis. 2026 Jul 22;2026:5552728. doi: 10.1155/tbed/5552728 (PMC13390018; doi:10.1155/tbed/5552728)
Supplement: Supplementary file 20 — Supporting Information 20 Figure S8: Multilocus genotype structure of Dermacentor reticulatus (n = 160) from Anatolia based on concatenated mitochondrial cox1 and nuclear ITS2 sequences. (A) Geographic distribution of multilocus genotypes across sampling localities. The upper panel shows the overall study area, with enlargements of (A1) Central Anatolia and (A2) Northeastern Anatolia. Pie charts represent genotype composition at each site. Colors denote regional affiliation: Central Anatolia (red shades), Northeastern Anatolia (blue shades), and shared genotypes (yellow–green shades). (B) TCS genotype network inferred from concatenated dataset. Circle size is proportional to genotype frequency, and each connection represents a single mutational step unless otherwise indicated. Colors correspond to geographic origin (Central Anatolia = red; Northeastern Anatolia = blue). (C) Color‐coded matrix of pairwise nucleotide identity among multilocus haplotypes. [file TBED-2026-5552728-s007.pdf]

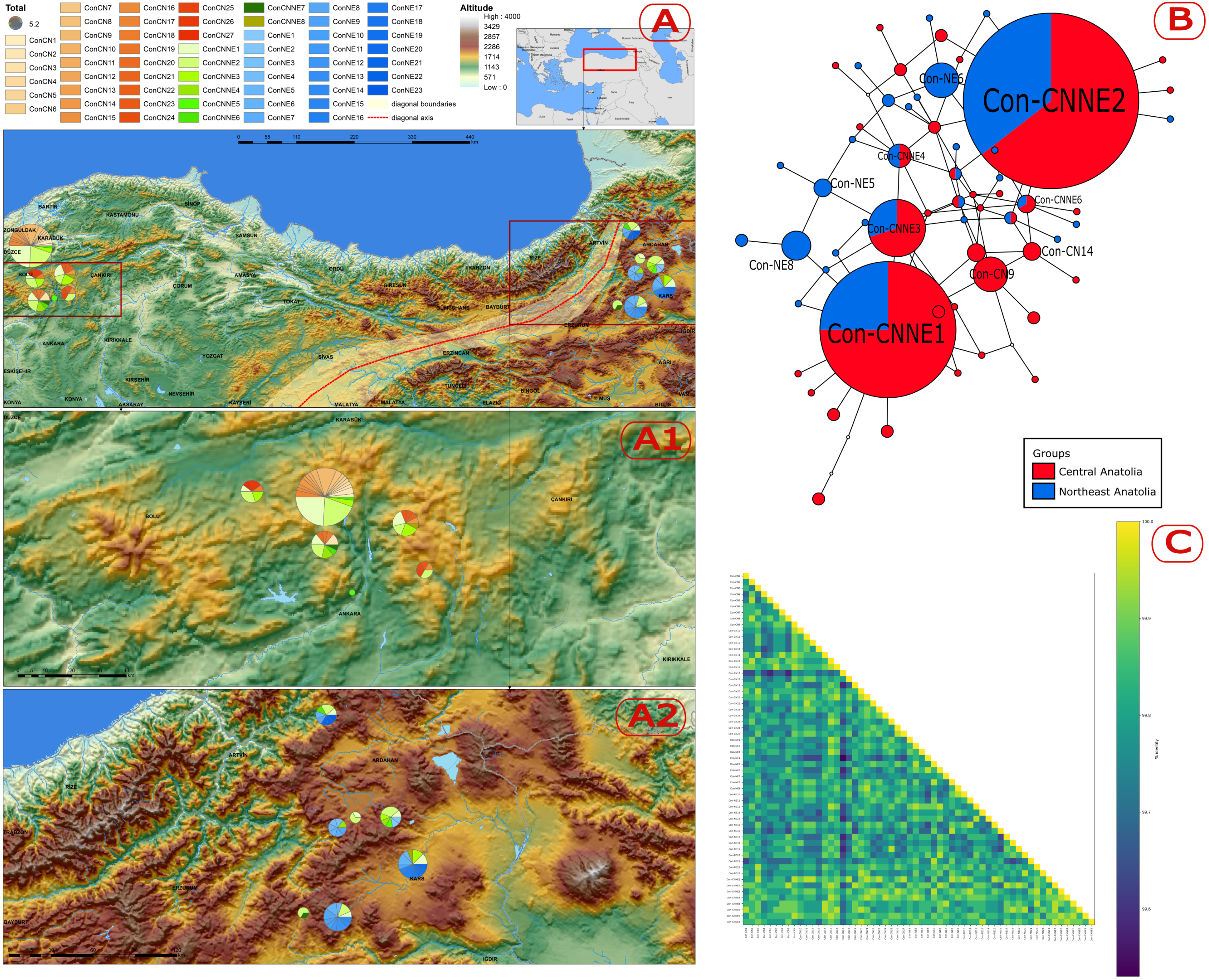
A
Total
5.2
ConCN1
ConCN2
ConCN3
ConCN4
ConCN5
ConCN6
ConCN7
ConCN8
ConCN9
ConCN10
ConCN11
ConCN12
ConCN13
ConCN14
ConCN15
ConCN16
ConCN17
ConCN18
ConCN19
ConCN20
ConCN21
ConCN22
ConCN23
ConCN24
ConCN25
ConCN26
ConCN27
ConCNNE1
ConCNNE2
ConCNNE3
ConCNNE4
ConCNNE5
ConCNNE6
ConCNNE7
ConCNNE8
ConNE1
ConNE2
ConNE3
ConNE4
ConNE5
ConNE6
ConNE7
ConNE8
ConNE9
ConNE10
ConNE11
ConNE12
ConNE13
ConNE14
ConNE15
ConNE16
ConNE17
ConNE18
ConNE19
ConNE20
ConNE21
ConNE22
ConNE23
diagonal boundaries
diagonal axis
Altitude
High : 4000
3429
2857
2286
1714
1143
571
Low : 0
0 55 110 220 330 440 km
BARTIN
KASTAMONU
SINOP
ZONGULDAK
KARABÜK
DÜZCE
BOLU
ÇANKIRI
SAMSUN
ÇORUM
AMASYA
ORDU
TOKAT
GİRESUN
TRABZON
RİZE
ARTVİN
ARDAHAN
KARS
GÜMÜŞHANE
BAYBURT
ERZURUM
IĞDIR
ANKARA
KIRIKKALE
YOZGAT
SİVAS
ERZİNCAN
AĞRI
ESKİŞEHİR
KIRŞEHİR
NEVŞEHİR
KAYSERİ
TUNCELİ
BİNGÖL
MUŞ
VAN
KONYA
AKSARAY
MALATYA
ELAZIĞ
BİTLİS
A1
DÜZCE
KARABÜK
BOLU
ÇANKIRI
ANKARA
KIRIKKALE
0 5 10 20 30 40 km
A2
ARTVİN
RİZE
TRABZON
ARDAHAN
KARS
ERZURUM
BAYBURT
IĞDIR
B
Con-CNNE2
Con-NE6
Con-CNNE4
Con-NE5
Con-CNNE6
Con-CNNE3
Con-CN14
Con-NE8
Con-CN9
Con-CNNE1
Groups
Central Anatolia
Northeast Anatolia
C
100.0
99.9
99.8
99.7
99.6
% identity
